# Supplementary material for: Mathematical modeling of in vitro replication dynamics for multiple highly pathogenic avian influenza clade 2.3.4.4 viruses in chicken and duck cells
Source: Microbiol Spectr. 2026 Oct 8;14(11):e00843-26. doi: 10.1128/spectrum.00843-26 (PMC13729145; doi:10.1128/spectrum.00843-26)
Supplement: Supplementary file 1 [file reviewer-comments.pdf]

ASM | Microbiology Spectrum

Mathematical modelling of in vitro replication dynamics for multiple highly pathogenic avian influenza clade 2.3.4.4 viruses in chicken and duck cells.

Luca Bordes, Peter Hobbelen, Elena Blokker, WH van der Poel, Catherine Beauchemin, Nancy Beerens, and Jose Gonzales

Corresponding Author(s): Luca Bordes, Wageningen Bioveterinary Research

**Review Timeline:**	Submission Date:	March 9, 2026
	Editorial Decision:	May 11, 2026
	Revision Received:	July 10, 2026
	Accepted:	July 26, 2026

Editor: Tao Deng

Reviewer(s): The reviewers have opted to remain anonymous.

Transaction Report:

(Note: With the exception of the correction of typographical or spelling errors that could be a source of ambiguity, letters and reports are not edited. The original formatting of letters and referee reports may not be reflected in this compilation.)

DOI: https://doi.org/10.1128/spectrum.00843-26

Re: Spectrum00843-26 (Mathematical modelling of in vitro replication dynamics for multiple highly pathogenic avian influenza clade 2.3.4.4 viruses in chicken and duck cells.)

Dear Dr. Luca Bordes:

Thank you for the privilege of reviewing your work. Below you will find my comments, instructions from the Spectrum editorial office, and the reviewer comments.

Please return the manuscript within 60 days; if you cannot complete the modification within this time period, please contact me. If you do not wish to modify the manuscript and prefer to submit it to another journal, notify me immediately so that the manuscript may be formally withdrawn from consideration by Spectrum.

Revision Guidelines
To submit your modified manuscript, log into the submission site at https://spectrum.msubmit.net/cgi-bin/main.plex. Go to Author Tasks and click the appropriate manuscript title to begin. The information you entered when you first submitted the paper will be displayed; update this as necessary. Note the following requirements:

• Upload point-by-point responses to the issues raised by the reviewers in a file named "Response to Reviewers," NOT in your cover letter.
• Upload a compare copy of the manuscript (without figures) as a "Marked-Up Manuscript" file.
• Upload a clean .DOC/.DOCX version of the revised manuscript and remove the previous version.
• Each figure must be uploaded as a separate, editable, high-resolution file (TIFF or EPS preferred), and any multipanel figures must be assembled into one file.
• Any supplemental material intended for posting by ASM should be uploaded with their legends separate from the main manuscript. You can combine all supplemental material into one file (preferred) or split it into a maximum of 10 files with all associated legends included.

For complete guidelines on revision requirements, see our Submission and Review Process webpage. Submission of a paper that does not conform to guidelines may delay acceptance of your manuscript.

Data availability: ASM policy requires that data be available to the public upon online posting of the article, so please verify all links to sequence records, if present, and make sure that each number retrieves the full record of the data. If a new accession number is not linked or a link is broken, provide Spectrum production staff with the correct URL for the record. If the accession numbers for new data are not publicly accessible before the expected online posting of the article, publication may be delayed; please contact production staff (Spectrum@asmusa.org) immediately with the expected release date.

Publication Fees: For information on publication fees and which article types are subject to charges, visit our website. If your manuscript is accepted for publication and any fees apply, you will be contacted separately about payment during the production process; please follow the instructions in that e-mail. Arrangements for payment must be made before your article is published.

ASM Membership: Corresponding authors may join or renew ASM membership to obtain discounts on publication fees. Need to upgrade your membership level? Please contact Customer Service at Service@asmusa.org.

The ASM Journals program strives for constant improvement in our submission and publication process. Please tell us how we can improve your experience by taking this quick Author Survey.

Thank you for submitting your paper to Spectrum.

Sincerely,
Tao Deng
Editor
Microbiology Spectrum

Reviewer #1 (Comments for the Author):

In this study, the authors used primary fibroblasts from chicken versus duck embryos to determine in vitro replication dynamics of genetically similar highly pathogenic H5 influenza viruses from chicken, duck and gull and to integrate the data into a mathematical model to assess and compare the viral fitness of the viruses. The mathematical model divides the cell population in susceptible, latent, infectious and dead compartments. The data available so far in this manuscript suggest that this approach

can be quite useful for investigating patterns of replication and pathogenicity in vitro and for making predictions about in vivo infections. At the same time, the preliminary data available in this manuscript indicate that significantly more isolates need to be included in the analyses to demonstrate that the model works reliably and is suitable to identify and predict patterns of pathogenicity or replication dynamics.

Major comments:

1) At least a few more virus isolates from ducks (currently only 2 out of 8 viruses) should be included.

2) In general, it would be important to also include LPAIV in the investigations. The authors themselves raise this point in the discussion of the manuscript but explain that primary fibroblasts do not represent a suitable system for this purpose. This is correct and is due to the lack of expression of trypsin-like cellular proteases that process the viral hemagglutinin and thereby confer infectivity. A very good alternative infection model to compare HPAIV and LPAIV is provided by primary chicken/duck embryo kidney epithelial cells. These can be isolated from embryos almost as easily as CEFs/DEFs and express suitable activating proteases for both LPAIV and HPAIV.

3) The individual virus isolates (swab samples from chicken or duck) were passaged one to two times in embryonated chicken eggs. During this process, mutations in the PB2 protein appear to have been acquired for both duck isolates (Table 1). Influenza viruses mutate very rapidly during passage in chicken eggs, and these may represent initial adaptations from duck to chicken. The authors should comment on the extent to which it is known whether PB2 I30L and V344A represent adaptations to replication in poultry. In general, to avoid such mutations, duck viruses should be propagated in duck eggs - analogous to the propagation of chicken viruses in chicken eggs.

Minor suggestions:
Furthermore, it would be interesting to determine the different replication parameters in endothelial cells of chicken versus duck for the HPAIV strains used here (and additional ones). The endothelial tropism of HPAIV in chickens and ducks represents a key difference in viral pathogenesis.

Reviewer #2 (Comments for the Author):

Efforts to move viral infection systems from animal experiments to tissue culture are valuable for many reasons including cost, rapidity, and ethics. Due in part to their inherent lower variability, cell culture experimental infections with virus can serve as a source for training mathematical models that can predict virus infection traits. Such models exist for human cell lines, and have been utilized to predict or validate phenotypic data observed from animal studies. However, a model has yet to be developed for avian cell culture despite birds being the natural host for influenza A viruses. Bordes et al. measure time to death and peak virus titer for avian isolates of influenza viruses in chicken and duck fibroblasts, and then construct mathematical models to predict traits including cell-intrinsic eclipse period, infectious period, and others. The manuscript is well-written and the experiments are sound. The work could be strengthened by validating the prediction of such traits derived from mathematical models.

Major points:
• Model validation. Empiric testing of model-derived parameters requires validation, at least for one or some of them. Infecting time, generation time, R0.
• Predictive possibility of the model. how would the model predict variations to infection, and how does this match-up with empiric result? For example, introduce neutralizing antibody or antiviral, or try virus reassortants.

Minor points:
• Citation #20 is published in Epidemics.

Reviewers' comments:

Reviewer #1 (Comments for the Author):

In this study, the authors used primary fibroblasts from chicken versus duck embryos to determine in vitro replication dynamics of genetically similar highly pathogenic H5 influenza viruses from chicken, duck and gull and to integrate the data into a mathematical model to assess and compare the viral fitness of the viruses. The mathematical model divides the cell population in susceptible, latent, infectious and dead compartments. The data available so far in this manuscript suggest that this approach can be quite useful for investigating patterns of replication and pathogenicity in vitro and for making predictions about in vivo infections. At the same time, the preliminary data available in this manuscript indicate that significantly more isolates need to be included in the analyses to demonstrate that the model works reliably and is suitable to identify and predict patterns of pathogenicity or replication dynamics.

Answer:

We thank the reviewer for careful reading of the manuscript and for the constructive suggestions to improve the manuscript. The individual points raised by the reviewer are answered below.

Major comments:

1) At least a few more virus isolates from ducks (currently only 2 out of 8 viruses) should be included.

Answer:

Virus selection was based on index cases in the Netherlands which included two viruses which were isolated from Pekin ducks. In this study we investigated the potential usage of mathematical modelling to improve the characterization of viral fitness of HPAI viruses and hypothesize how the model could be used in future studies to investigate phenotypic differences between HPAI viruses. This requires another study design which is beyond the scope of this investigation.

2) In general, it would be important to also include LPAIV in the investigations. The authors themselves raise this point in the discussion of the manuscript but explain that primary fibroblasts do not represent a suitable system for this purpose. This is correct and is due to the lack of expression of trypsin-like cellular proteases that process the viral hemagglutinin and thereby confer infectivity. A very good alternative infection model to compare HPAIV and LPAIV is provided by primary chicken/duck embryo kidney epithelial cells. These can be isolated from embryos almost as easily as CEFs/DEFs and express suitable activating proteases for both LPAIV and HPAIV.

The manuscript has been adapted to highlight the potential usage of chicken/duck embryonic kidney epithelial cells. Lines 512-514: “Alternatively, primary embryo kidney cells, intestinal organoids or airway organoids can be used to expand the model to LPAI as these cells endogenously express trypsin-like proteases”

3) The individual virus isolates (swab samples from chicken or duck) were passaged one to two times in embryonated chicken eggs. During this process, mutations in the PB2 protein appear to have been acquired for both duck isolates (Table 1). Influenza viruses mutate very rapidly during passage in chicken eggs, and these may represent initial adaptations from duck to chicken. The authors should comment on the extent to which it is known whether PB2 I30L and V344A represent adaptations to replication in poultry. In general, to avoid such mutations, duck viruses should be propagated in duck eggs - analogous to the propagation of chicken viruses in chicken eggs.

See lines 152-153: “Only minor differences between the sequences of the swabs and egg passages were detected, and no mutations on positions associated with virulence changes were identified.”

Minor suggestions:

Furthermore, it would be interesting to determine the different replication parameters in endothelial cells of chicken versus duck for the HPAIV strains used here (and additional ones). The endothelial tropism of HPAIV in chickens and ducks represents a key difference in viral pathogenesis.

The manuscript has been adapted in lines 514-515: “For Pekin ducks, in vitro virulence may be better assessed in endothelial cell models, as endothelial tropism is a key determinant of HPAIV pathogenesis”

Reviewer #2 (Comments for the Author):

Efforts to move viral infection systems from animal experiments to tissue culture are valuable for many reasons including cost, rapidity, and ethics. Due in part to their inherent lower variability, cell culture experimental infections with virus can serve as a source for training mathematical models that can predict virus infection traits. Such models exist for human cell lines, and have been utilized to predict or validate phenotypic data observed from animal studies. However, a model has yet to be developed for avian cell culture despite birds being the natural host for influenza A viruses. Bordes et al. measure time to death and peak virus titer for avian isolates of influenza viruses in chicken and duck fibroblasts, and then construct mathematical models to predict traits including cell-intrinsic eclipse period, infectious period, and others. The manuscript is well-written and the experiments are sound. The work could be strengthened by validating the prediction of such traits derived from mathematical models.

Answer:

We thank the reviewer for careful reading of the manuscript and for the constructive suggestions to improve the manuscript. The individual points raised by the reviewer are answered below.

Major points:

• Model validation. Empiric testing of model-derived parameters requires validation, at least for one or some of them. Infecting time, generation time, R0.

We agree with the reviewer that it would be good to validate derived measures like the R0, infecting time and generation time, since we are exploring how these measures can be linked to effects at the animal and population level. For this purpose, at least the rank order of virus strains based on a certain measure should be consistent with reality. To validate this, we would need to compare model-derived measures to experimentally measured values from independent experiments. Conducting these experiments is not easy, since you need to be able to introduce a predetermined number of infectious cells into a susceptible population and track the secondary infections by these cells. This is beyond the scope of this investigation. We added a section in lines 521-534 to emphasize the importance of validation of these summary measures and provided suggestions how to approach this in future studies.

• Predictive possibility of the model. how would the model predict variations to infection, and how does this match-up with empiric result? For example, introduce neutralizing antibody or antiviral, or try virus reassortants.

In our view, model validation (the first major point) and predictive possibility are related, since validation involves a comparison of predicted and observed values. Our reply to the first major point of the reviewer therefore also applies to this point. From a practical point of view, our model is fit-for-purpose if it can correctly predict the rank order of virus strains based on summary measures such as the R0, infecting time and generation time. To validate this, we would need to compare model-derived estimates of these measures to experimentally measured values from independent experiments. As explained above, this is not a trivial exercise and is part of our future research plans. We agree with the reviewer that the predictive power of the model could be further tested by predicting the outcome of experiments involving different more complicated dynamics due to the presence of e.g. neutralizing antibodies and antivirals. Even though we do not intend to use our model in this way, it would further boost confidence in the accuracy of model-derived parameters such as the R0, infecting time and generation time. We

added a section to the discussion to emphasize the importance of validation of these summary measures and our future plans for this. Lines 521-534

Minor points:

- Citation #20 is published in Epidemics.

We thank the reviewer for noting this. The reference has been revised accordingly.

Re: Spectrum00843-26R1 (Mathematical modelling of in vitro replication dynamics for multiple highly pathogenic avian influenza clade 2.3.4.4 viruses in chicken and duck cells.)

Dear Dr. Luca Bordes:

Your manuscript has been accepted, and I am forwarding it to the ASM production staff for publication. Your paper will first be checked to make sure all elements meet the technical requirements. ASM staff will contact you if anything needs to be revised before copyediting and production can begin. Otherwise, you will be notified when your proofs are ready to be viewed.

Data Availability: ASM policy requires that data be available to the public upon online posting of the article, so please verify all links to sequence records, if present, and make sure that each number retrieves the full record of the data. If a new accession number is not linked or a link is broken, provide production staff with the correct URL for the record. If the accession numbers for new data are not publicly accessible before the expected online posting of the article, publication may be delayed; please contact ASM production staff immediately with the expected release date.

Publication Fees: For information on publication fees and which article types have charges, please visit our website. We have partnered with Copyright Clearance Center (CCC) to collect author charges. If fees apply to your paper, you will receive a message from no-reply@copyright.com with further instructions. For questions related to paying charges through RightsLink, please contact CCC at ASM_Support@copyright.com or toll free at +1-877-622-5543. CCC makes every attempt to respond to all emails within 24 hours.

ASM Membership: Corresponding authors may join or renew ASM membership to obtain discounts on publication fees. Need to upgrade your membership level? Please contact Customer Service at Service@asmusa.org.

Embargo Policy: A press release may be issued as soon as the manuscript is posted on the Spectrum Latest Articles webpage. The corresponding author will receive an email with the subject line "ASM Journals Author Services Notification" when the article is available online.

Cover Image Submissions: If you would like to submit a potential Cover Image, please email a file and a short legend to spectrum@asmusa.org. Please note that we can only consider images that (i) the authors created or own and (ii) have not been previously published. By submitting, you agree that the image can be used under the same terms as the published article. Image File requirements: TIF/EPS, 7.5 inches wide by 8.25 inches tall (at least 2,250 pixels wide by 2,475 pixels tall), minimum 300 dpi resolution (600 dpi preferred), RGB, and no figure elements, e.g., arrows or panel labels. The legend should be a short description of the image, 1-2 sentences recommended. Please download and use this interactive template in Adobe the https://journals.asm.org/pb-assets/html/asm-cover-sizing-tool.html to ensure that your proposed cover image meets our size requirements (https://journals.asm.org/pb-assets/pdf-text-excel-files/ASM-Interactive-Sizing-Cover-Template-1715689791.pdf).

The ASM Journals program strives for constant improvement in our submission and publication process. Please tell us how we can improve your experience by taking this quick Author Survey.

Thank you for submitting your paper to Spectrum.

Sincerely,
Tao Deng
Editor
Microbiology Spectrum

Reviewer #2 (Comments for the Author):

The expanded section on study limitations adequately addressed my comments.
