# Supplementary material for: Mathematical modeling of in vitro replication dynamics for multiple highly pathogenic avian influenza clade 2.3.4.4 viruses in chicken and duck cells
Source: Microbiol Spectr. 2026 Oct 8;14(11):e00843-26. doi: 10.1128/spectrum.00843-26 (PMC13729145; doi:10.1128/spectrum.00843-26)

Supplementary Information

Contents

1. Definitions, dimensions and initial values or prior distributions for state variables and parameters in the mathematical model
   - Tables S1 and S2
2. Genetic analysis and potential host shift adaptations
   - Tables S3 and S4
3. Replication dynamics without mathematical modelling
   - Figure S1
4. Summary of the posterior distributions of model parameters for each combination of virus strain and host cell
   - Figure S2.
5. Fine-tuning of the ABC-SMC algorithm
   - Figures S3 and S4
6. Assessment of the convergence of posterior distributions and parameter identifiability for each combination of virus-strain and host-cell
   - 6.1. H5N1-AB on CEF cells | Figures S5 to S7
   - 6.2. H5N1-AB on DEF cells | Figures S8 to S10
   - 6.3. H5N1-AC on CEF cells | Figures S11 to S13
   - 6.4. H5N1-AC on DEF cells | Figures S14 to S16
   - 6.5. H5N1-BB on CEF cells | Figures S17 to S19
   - 6.6. H5N1-BB on DEF cells | Figures S20 to S22
   - 6.7. H5N1-C on CEF cells | Figures S23 to S25
   - 6.8. H5N1-C on DEF cells | Figures S26 to S28
   - 6.9. H5N6-2017 on CEF cells | Figures S29 to S31
   - 6.10. H5N6-2017 on DEF cells | Figures S32 to S34
   - 6.11. H5N8-2014 on CEF cells | Figures S35 to S37
   - 6.12. H5N8-2014 on DEF cells | Figures S38 to S40
   - 6.13. H5N8-2016 on CEF cells | Figures S41 to S43
   - 6.14. H5N8-2016 on DEF cells | Figures S44 to S46
   - 6.15. H5N8-2020 on CEF cells | Figures S47to S49
   - 6.16. H5N8-2020 on DEF cells | Figures S50 to S52

1. Definitions, dimensions and initial values or prior distributions for state variables and parameters in the mathematical model

Table S1. The definitions, dimensions and initial values of state variables in the model.

State variable	Definition	Dimension	Initial value
S	Concentration of susceptible cells	cells/ml	175,000
E	Concentration of cells in eclipse phase	cells/ml	0
D	Concentration of dead cells	cells/ml	0
I	Concentration of infectious cells	cells/ml	0
V	Concentration of infectious virus particles	TCID50/ml	175

Table S2. The definitions, dimensions and posterior distributions of model parameters.

Parameter	Definition	Dimension	Prior distribution[1,2]
β	Transmission parameter	ml/(TCID50*h)	$U_c(-9,1)$ on $\log_{10}$ scale
τ_E	Eclipse period cells	h	$U_c(0,24)$
τ_I	Infectious period cells	h	$U_c(0.01,24)$
n	Shape parameter of the gamma distribution for the lengths of the eclipse and infectious periods	-	$U_d(1,30)$
p	Virus replication rate by infectious cells	TCID50/(cell*h)	$U_c(-2,3)$ on $\log_{10}$ scale
α	Infectious virus particles lost from the supernatant due to the infection of a cell.	TCID50/cell	$U_c(-2,2)$ on $\log_{10}$ scale
τ_V	Lifetime of an infectious virus particle	h	$U_c(-1,2)$ on $\log_{10}$ scale

[1]) Symbols U_c and U_d represent the continuous and discrete uniform distributions, respectively.

[2]) The prior distribution of some parameters was specified on a $\log_{10}$ instead of linear scale in order to increase the probability of sampling parameter values over a range spanning several orders of magnitude.

2. Genetic analysis and potential host shift adaptations

Table S3. Virulence factors that differ between the eight HPAI viruses. Coloured cells indicates the presence of the marker in the first column. Increased replication in mammalian cells (red), increased virulence in chickens (yellow), decreased virulence in mice (green), decreased replication in mammalian cells (blue) and disruption of the second sialic acid binding site (light green) is indicated by the colours.

Marker	Effect	H5N8-2014	H5N8-2016	H5N6-2017	H5N8-2020	H5N1-2020-C	H5N1-2021-AB	H5N1-2021-AC	H5N1-2022-BB
NS-1:P42S	Increased replication in mammalian cells	red							
NS-1:C138F,NS-1:K55E,NS-1:K66E	Increased replication in mammalian cells	red	red	red		red	red	red	red
NP:M105V	increased virulence in chickens		yellow	yellow	yellow	yellow		yellow	yellow
PB2:I292V	Increased replication in mammalian cells		red		red				
PB2:K389R	Increased replication in mammalian cells	red	red	red		red	red	red	red
PB2:L89V,PB2:G309D	Increased replication in mammalian cells	red	red	red	red	red	red		red
PB2:L89V,PB2:G309D,PB2:T339K,PB2:R477G,PB2:I495V,PB2:K627E,PB2:A676T	Increased replication in mammalian cells		red	red	red	red	red		red
PB1-F2:N66S	Increased replication in mammalian cells	red				red	red	red	red
PA:K158R	Increased replication in mammalian cells			red					
PA:Q400P	Decreased virulence in mice	green						green	
NP:Y52N	Decreased replication in mammalian cells								blue
NA-1:A369I	Distruption of the second sialic acid binding site (2SBS)								light green
NA-1:K432E	Distruption of the second sialic acid binding site (2SBS)	light green							

Table S4. Phylogenetic grouping of the selected HPAI viruses based on each genome segment. Different colors indicate sequences with p-distance ≤ 0.015 and is identified for each genome segment separately.

Abbreviation	**PB2**	**PB1**	**PA**	**HA**	**NP**	**NA**	**MP**	**NS**
H5N8-2014								
H5N8-2016								
H5N6-2017								
H5N8-2020								
H5N1-2020-C								
H5N1-2021-AB								
H5N1-2021-AC								
H5N1-2022-BB								

3. Replication dynamics without mathematical modelling

Figure S1. Chicken embryo fibroblasts stained with DAPI. Complete removal of the monolayer due to cytopathogenicity is observed at 34hpi except for minor positive staining for H5N1-2022-BB.

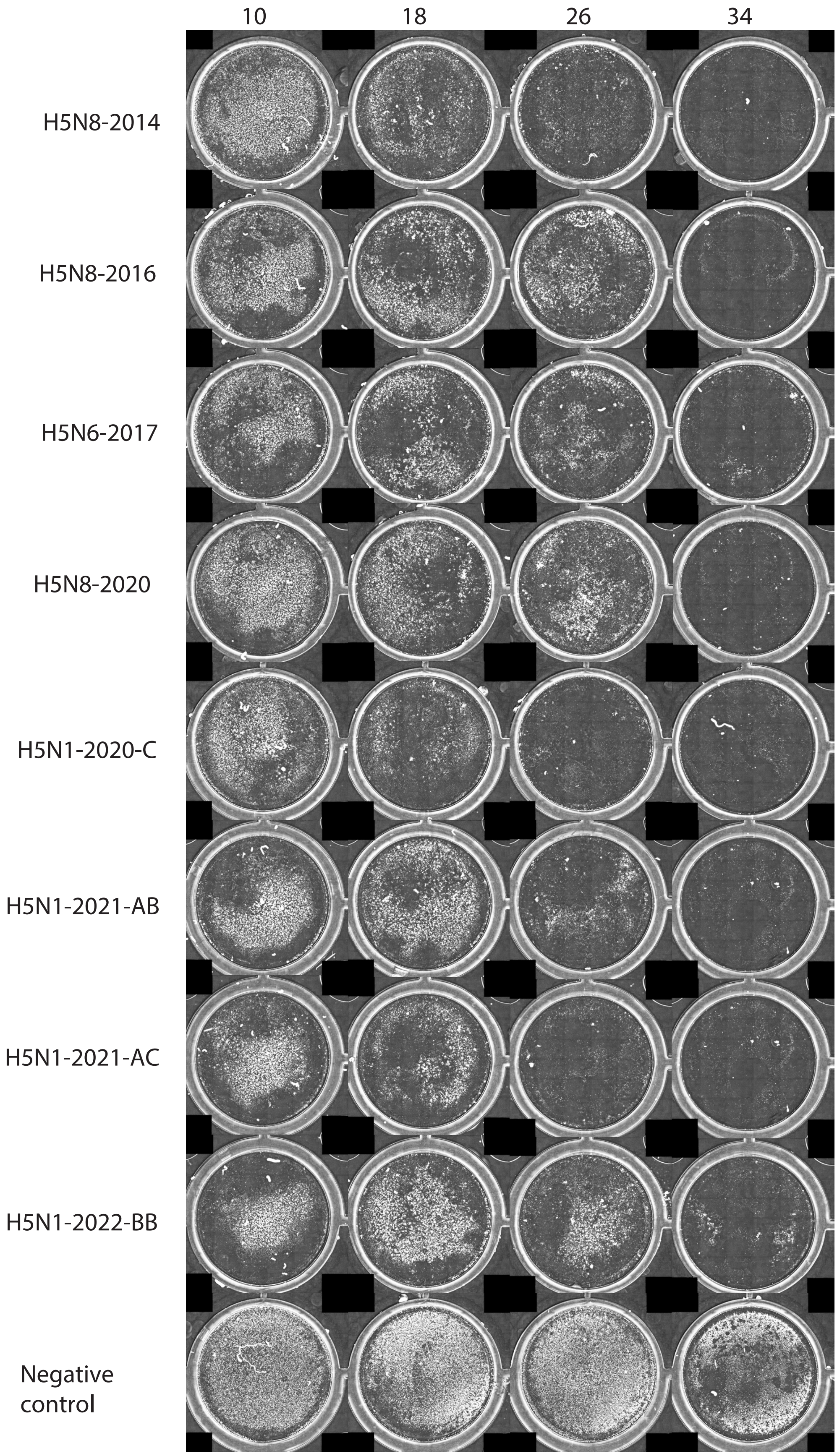

4. Summary of the posterior distributions of model parameters for each combination of virus strain and host cell

Figure S2. Additional model output parameters. The medians are displayed by the blue and red dots.

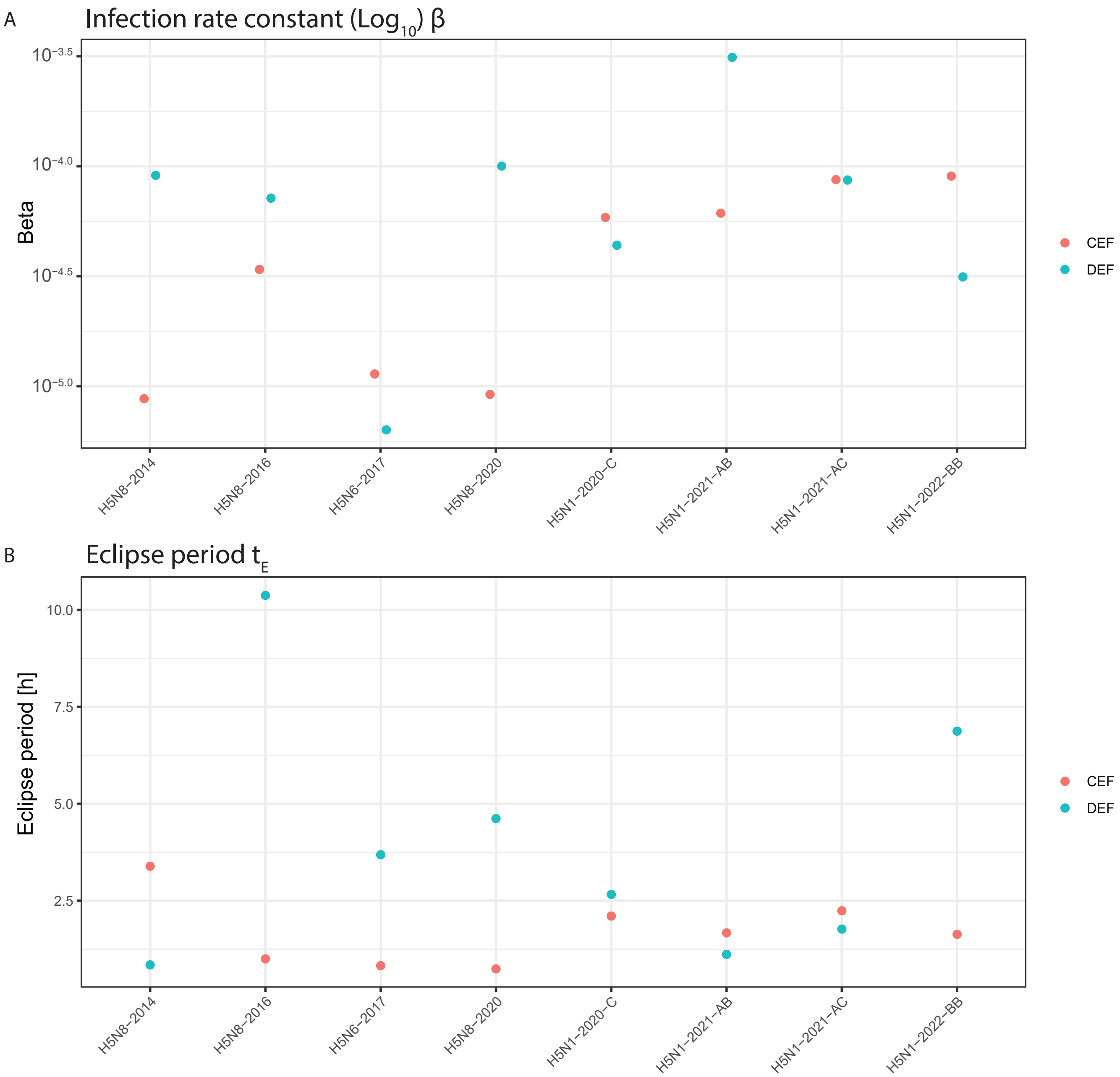

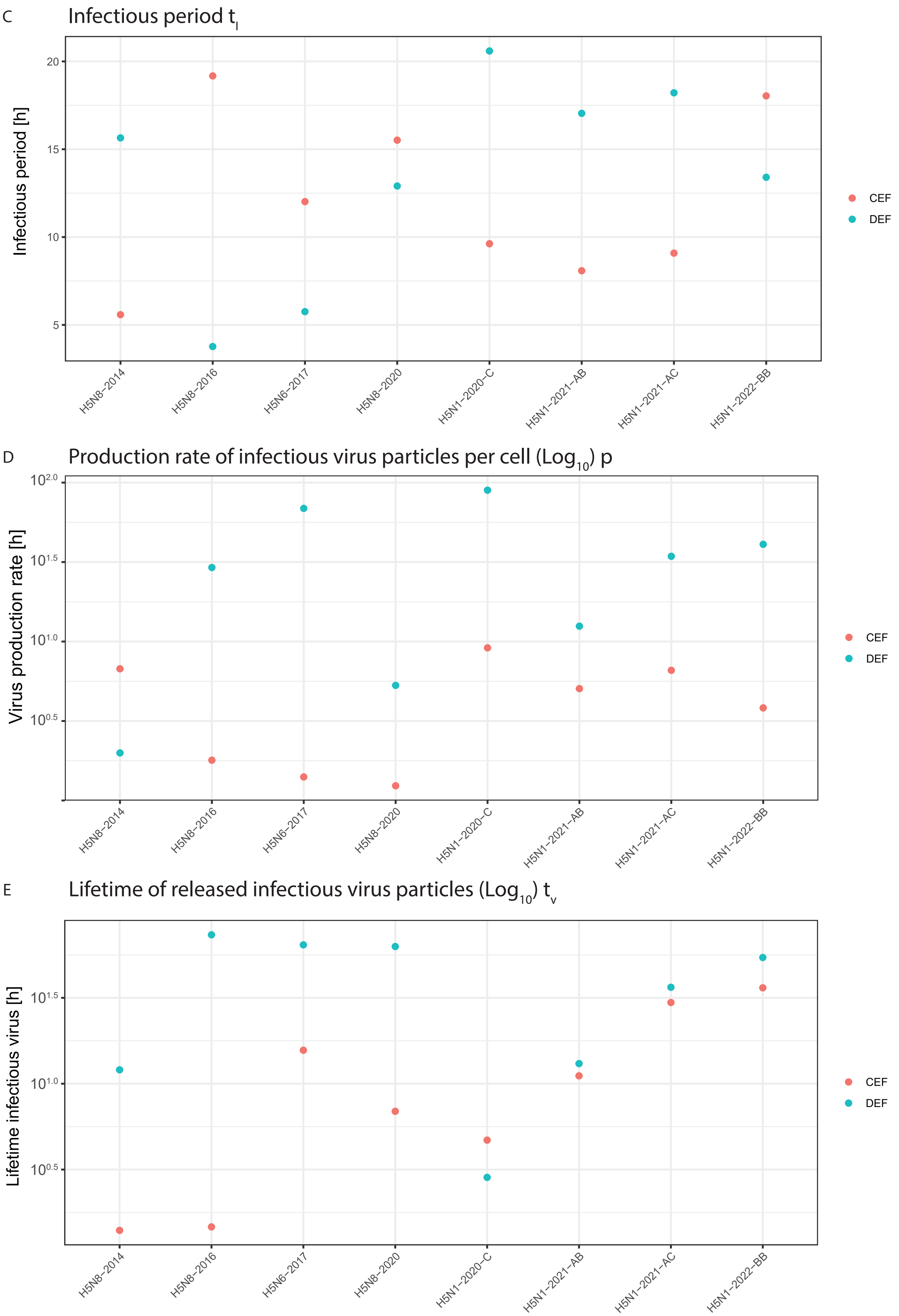
C
Infectious period t_I
Infectious period [h]
20
15
10
5
H5N8-2014
H5N8-2016
H5N6-2017
H5N8-2020
H5N1-2020-C
H5N1-2021-AB
H5N1-2021-AC
H5N1-2022-BB
CEF
DEF
D
Production rate of infectious virus particles per cell (Log10) p
Virus production rate [h]
10^2.0
10^1.5
10^1.0
10^0.5
H5N8-2014
H5N8-2016
H5N6-2017
H5N8-2020
H5N1-2020-C
H5N1-2021-AB
H5N1-2021-AC
H5N1-2022-BB
CEF
DEF
E
Lifetime of released infectious virus particles (Log10) t_v
Lifetime infectious virus [h]
10^1.5
10^1.0
10^0.5
H5N8-2014
H5N8-2016
H5N6-2017
H5N8-2020
H5N1-2020-C
H5N1-2021-AB
H5N1-2021-AC
H5N1-2022-BB
CEF
DEF

5. Fine-tuning of the ABC-SMC algorithm

Figure S3. The median and 95% credible interval (CI) of the posterior distribution of model parameters fitted to data for strain H5N1-AB on CEF cells for increasing values of the particle population size.

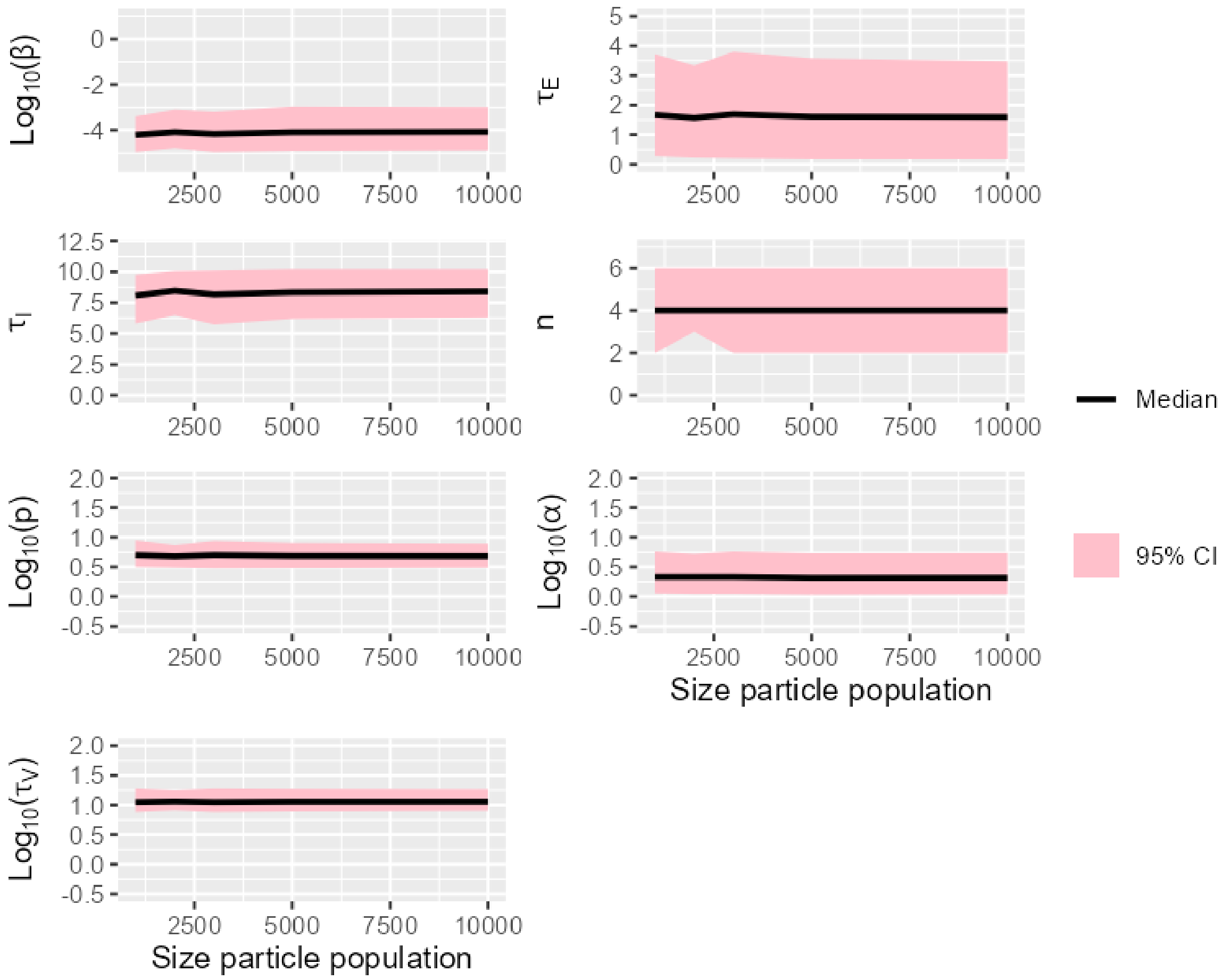

Figure S4. The median and 95% credible interval (CI) of the posterior distributions of model parameters fitted to data for strain H5N1-AB on CEF cells for increasing values of parameter θ, which influences the width of the perturbation kernel for continous parameters.

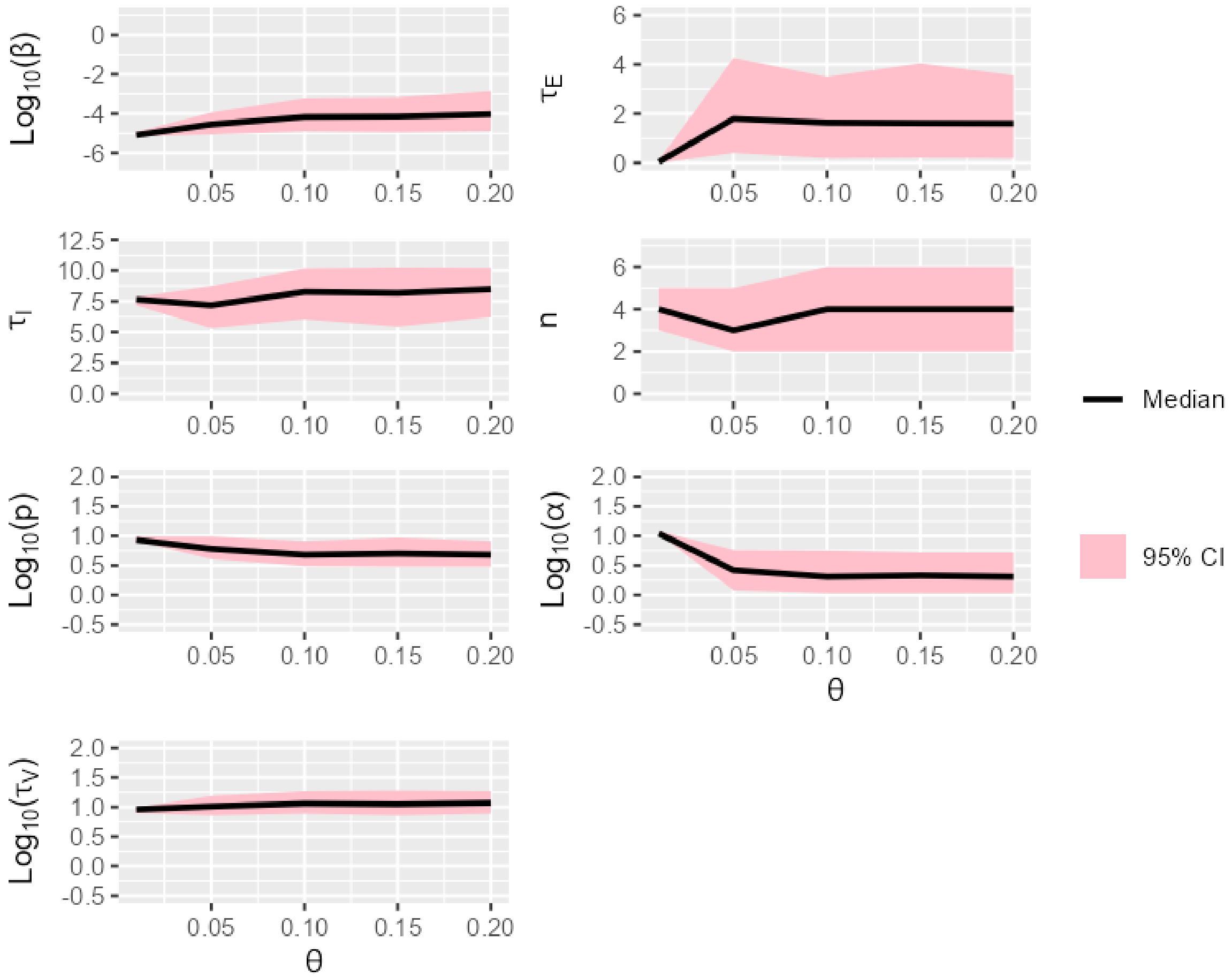

6. Assessment of the convergence of posterior distributions and parameter identifiability for each combination of virus-strain and host-cell

6.1 H5N1-AB on CEF cells

Figure S5. The shifts in the distributions of model parameters during the ABC-SMC analysis of data from strain H5N1-AB on CEF cells, when moving from the first particle population (containing accepted particles sampled from the prior distribution) to the posterior distribution via intermediate particle populations.

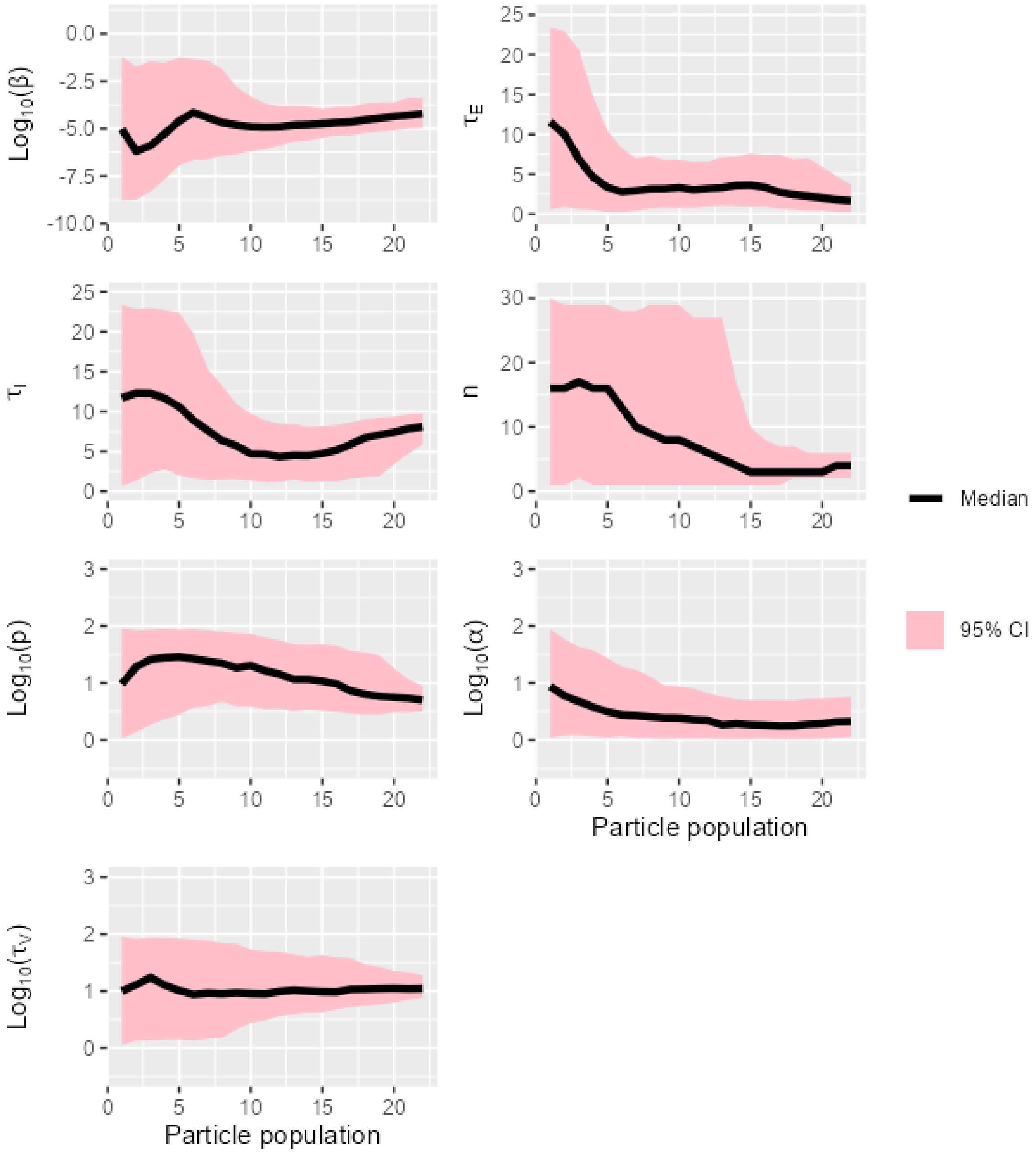

Figure S6. The overlap between prior and posterior distributions of model parameters for strain H5N1-AB on CEF cells.

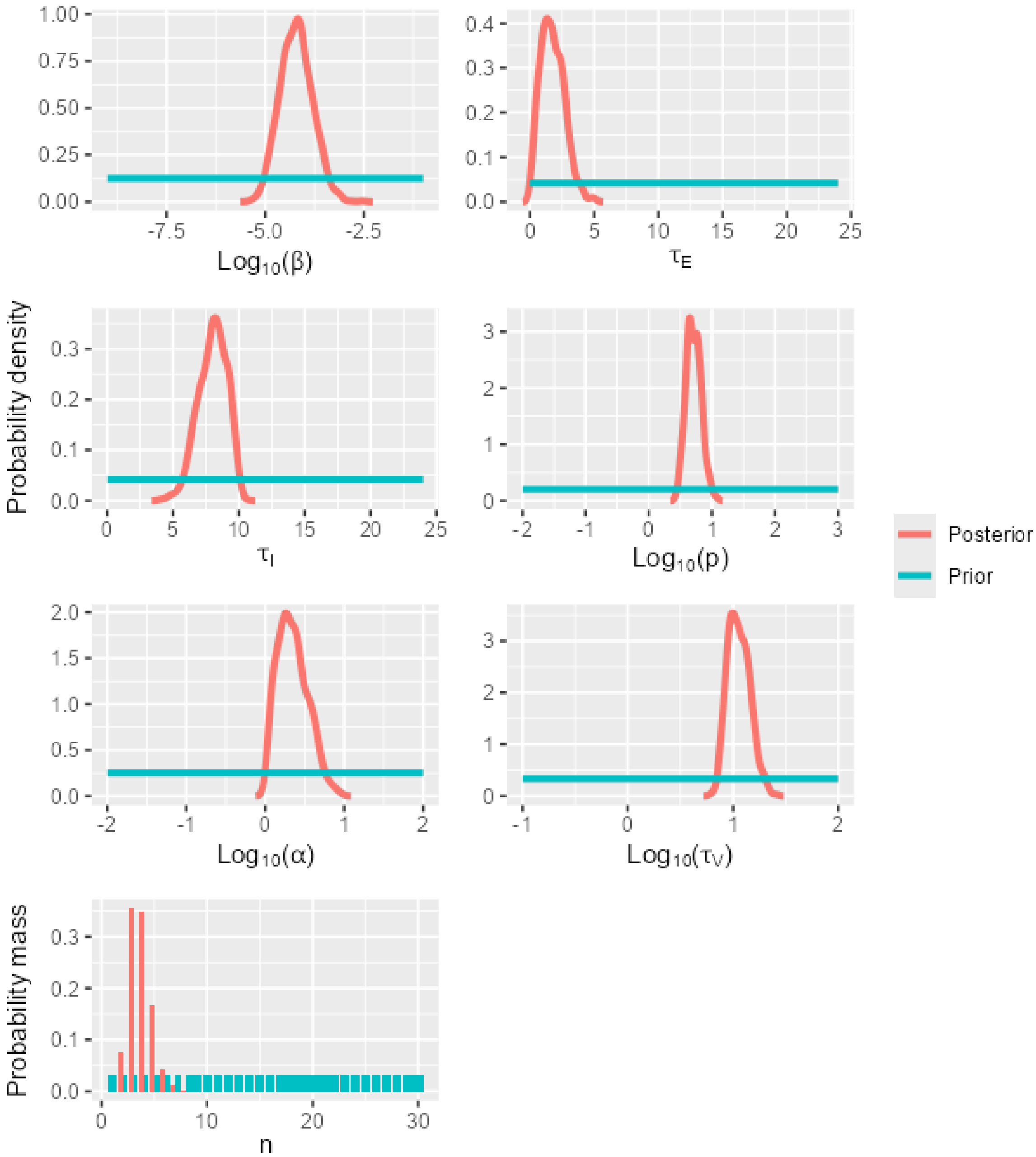

Figure S7. A matrix showing Kendall's rank correlation coefficient and the significance of the correlation (0.05 level, indicated by *) for pairs of model parameters calibrated using data for strain H5N1-AB on CEF cells.

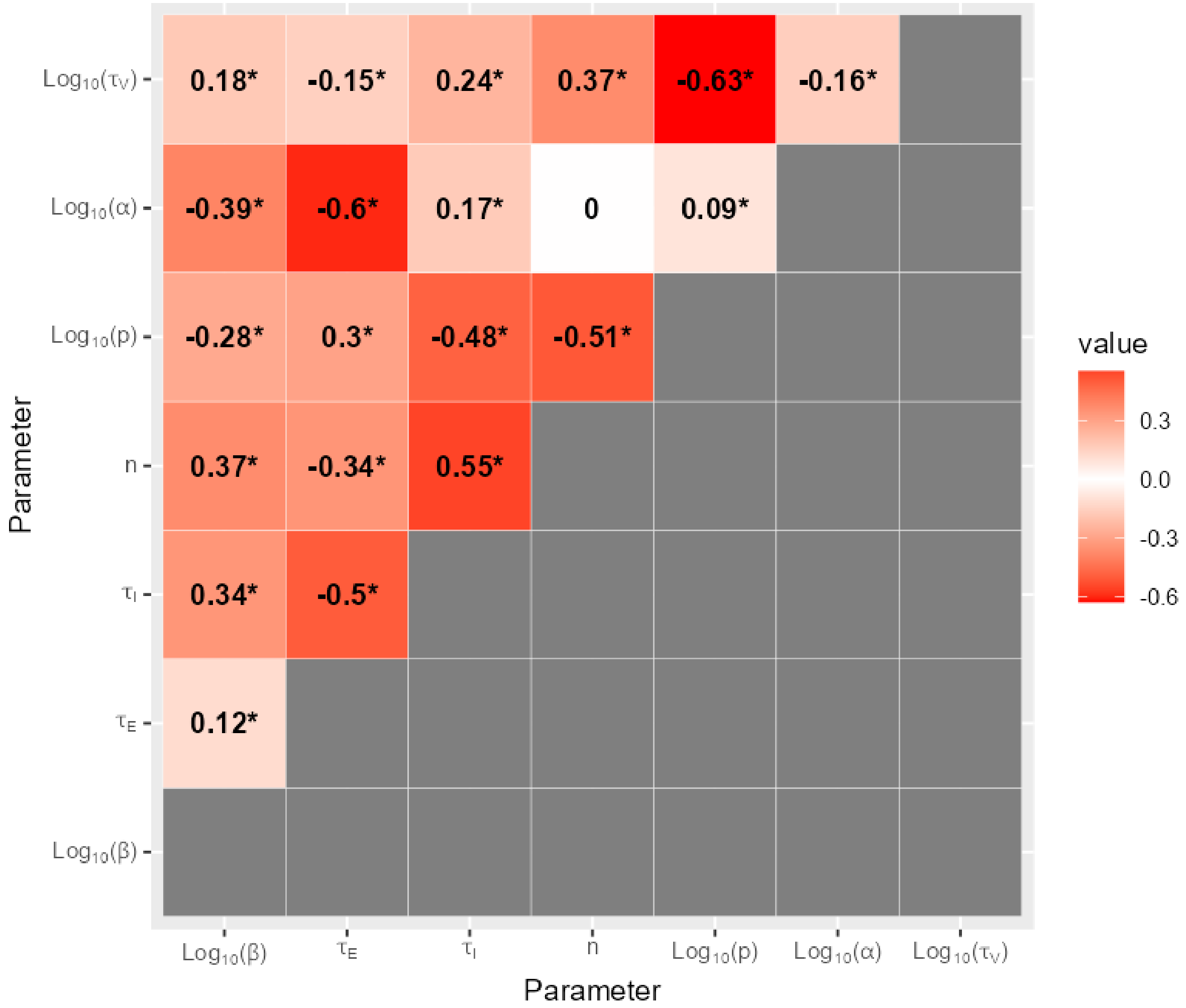

6.2 H5N1-AB on DEF cells

Figure S8. The shifts in the distributions of model parameters during the ABC-SMC analysis of data from strain H5N1-AB on DEF cells, when moving from the first particle population (containing accepted particles sampled from the prior distribution) to the posterior distribution via intermediate particle populations.

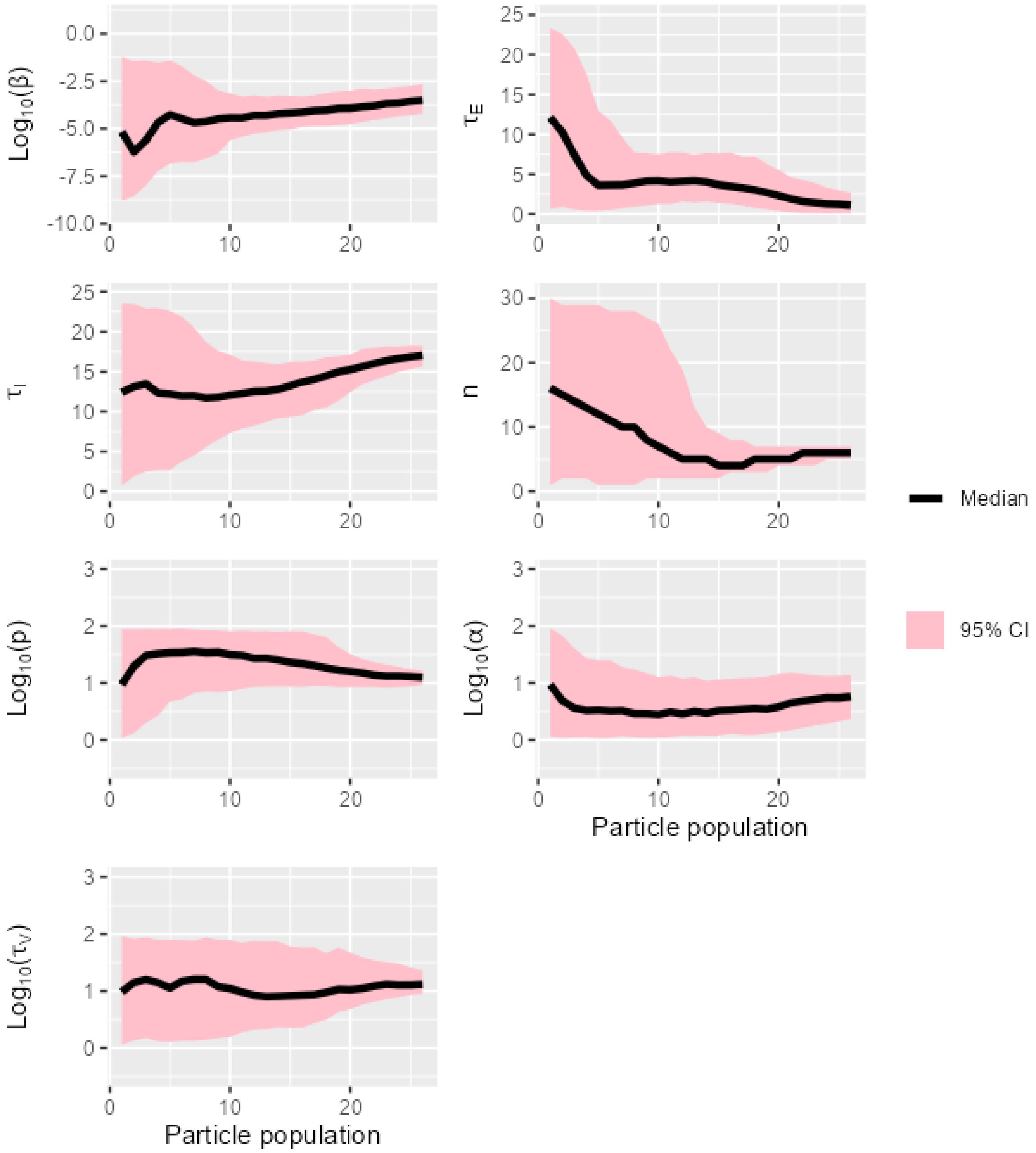

Figure S9. The overlap between prior and posterior distributions of model parameters for strain H5N1-AB on DEF cells.

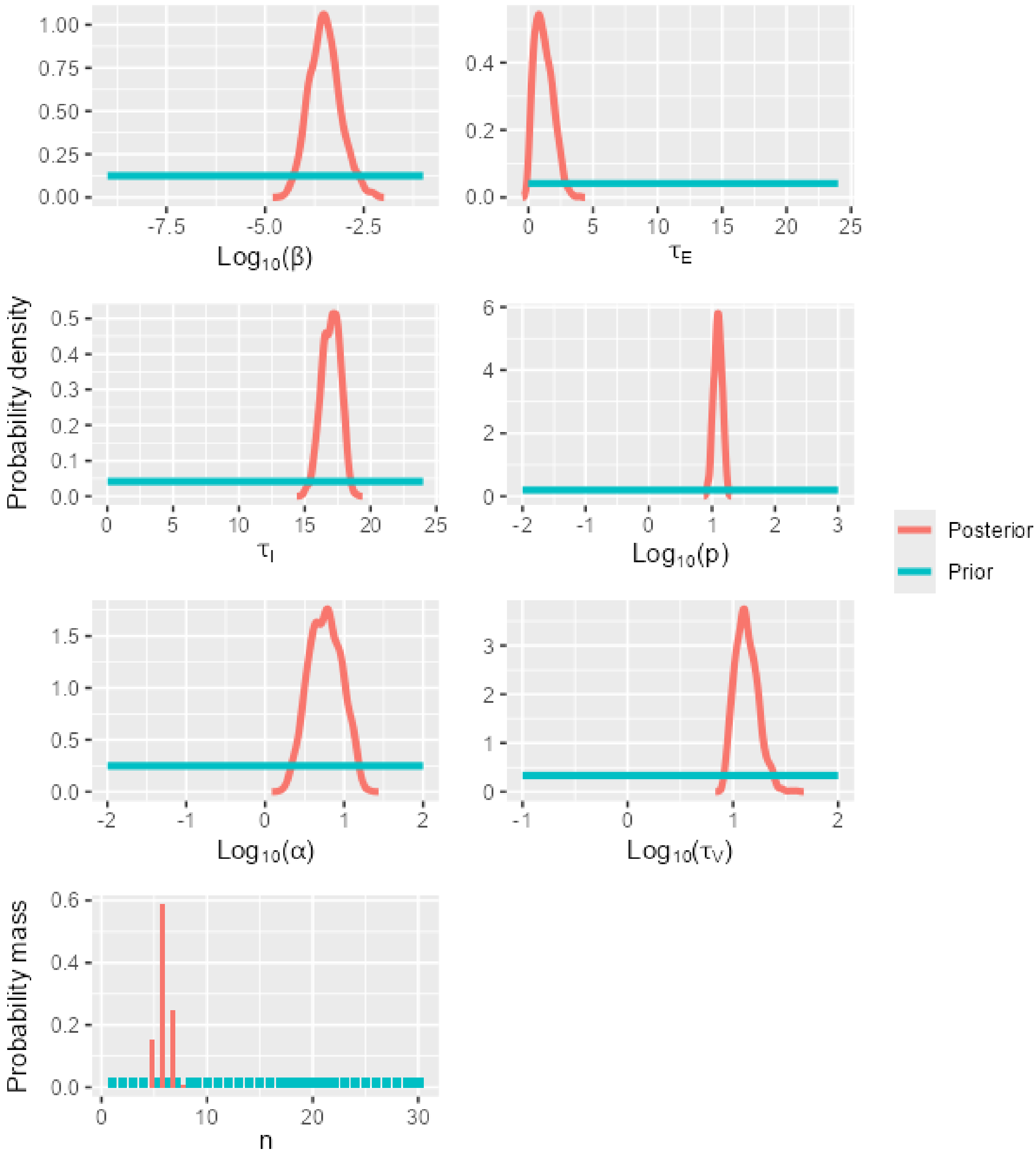

Figure S10. A matrix showing Kendall's rank correlation coefficient and the significance of the correlation (0.05 level, indicated by *) for pairs of model parameters calibrated using data for strain H5N1-AB on DEF cells.

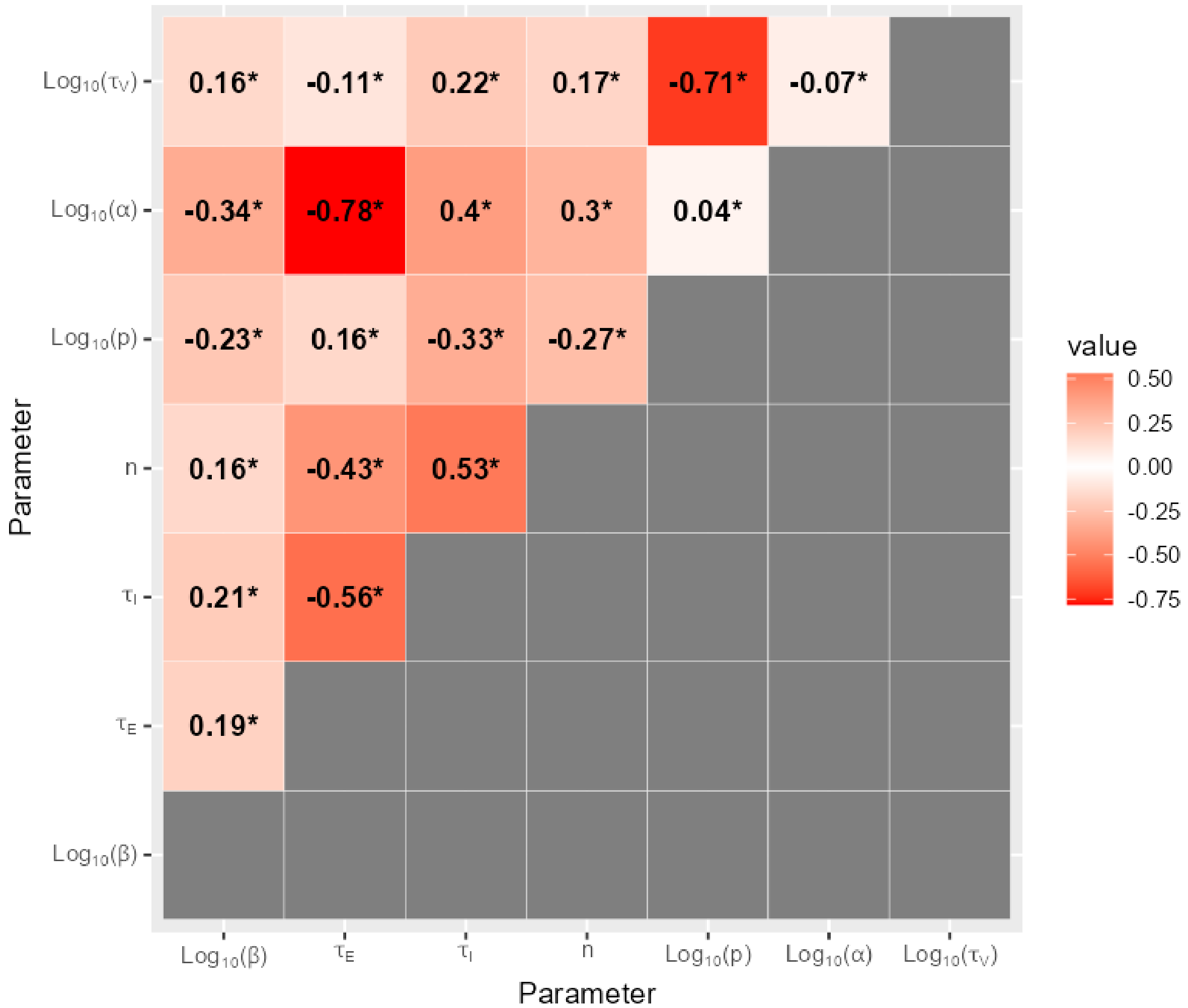

6.3 H5N1-AC on CEF cells

Figure S11. The shifts in the distributions of model parameters during the ABC-SMC analysis of data from strain H5N1-AC on CEF cells, when moving from the first particle population (containing accepted particles sampled from the prior distribution) to the posterior distribution via intermediate particle populations.

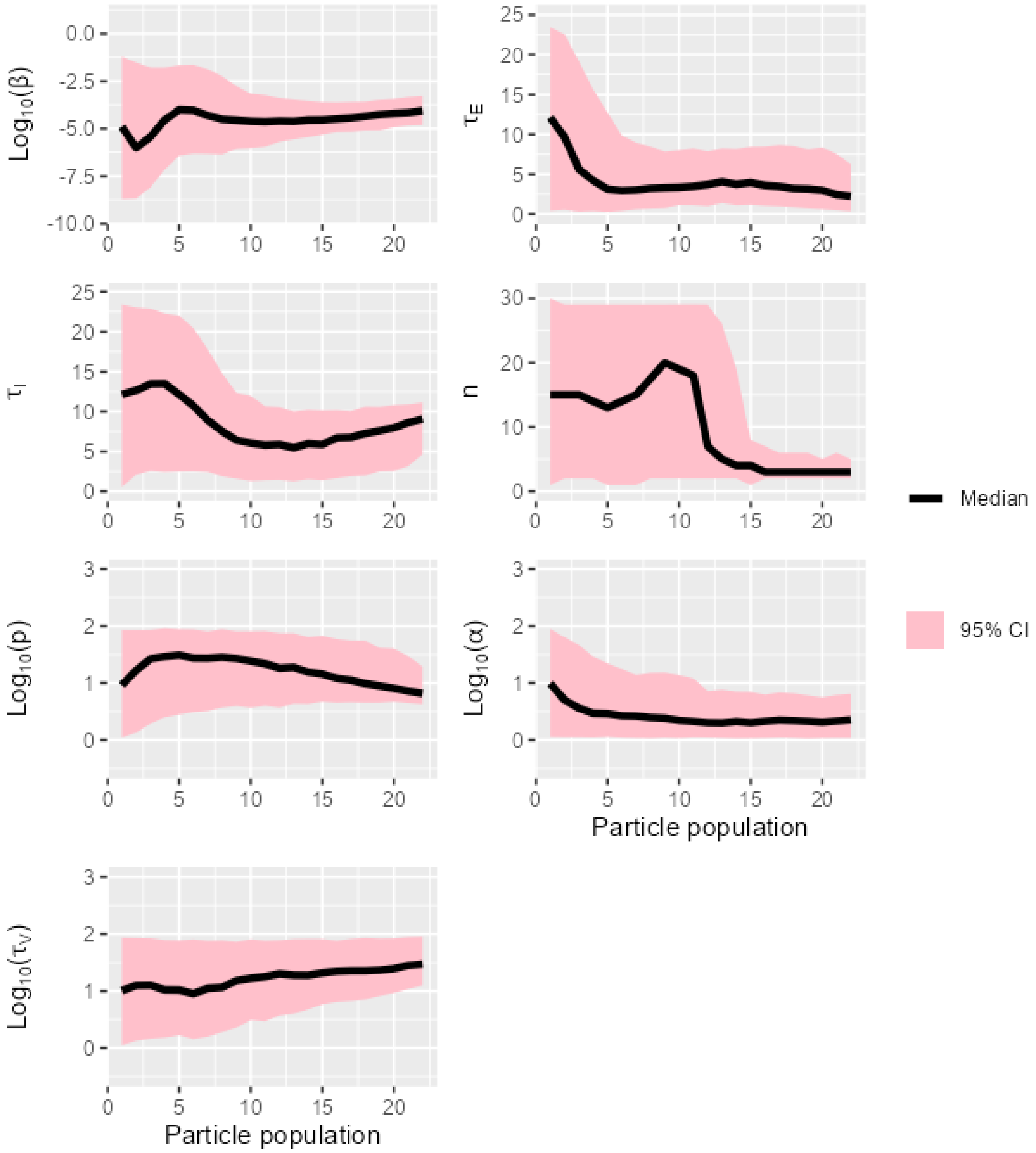

Figure S12. The overlap between prior and posterior distributions of model parameters for strain H5N1-AC on CEF cells.

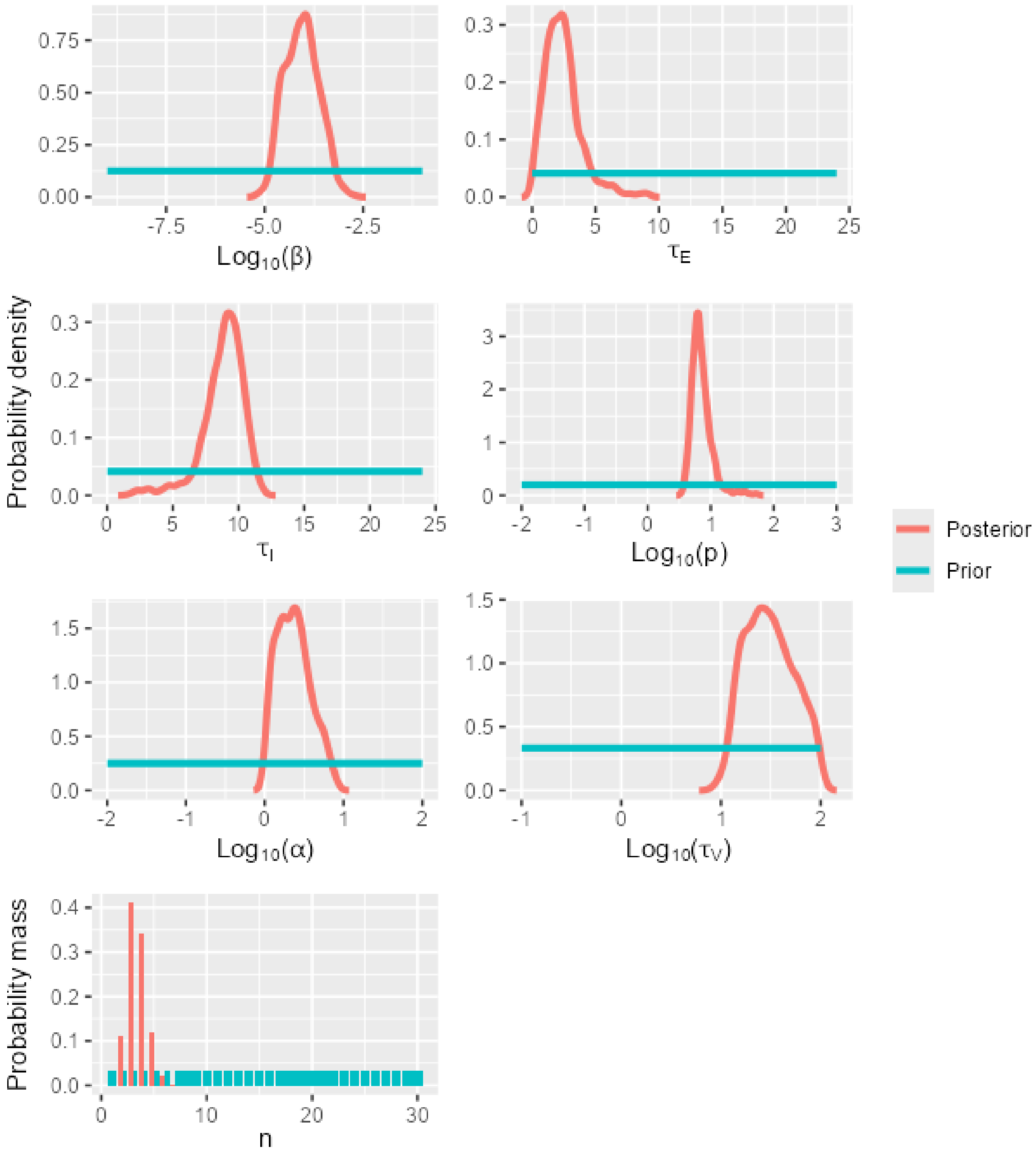

Figure S13. A matrix showing Kendall's rank correlation coefficient and the significance of the correlation (0.05 level, indicated by *) for pairs of model parameters calibrated using data for strain H5N1-AC on CEF cells.

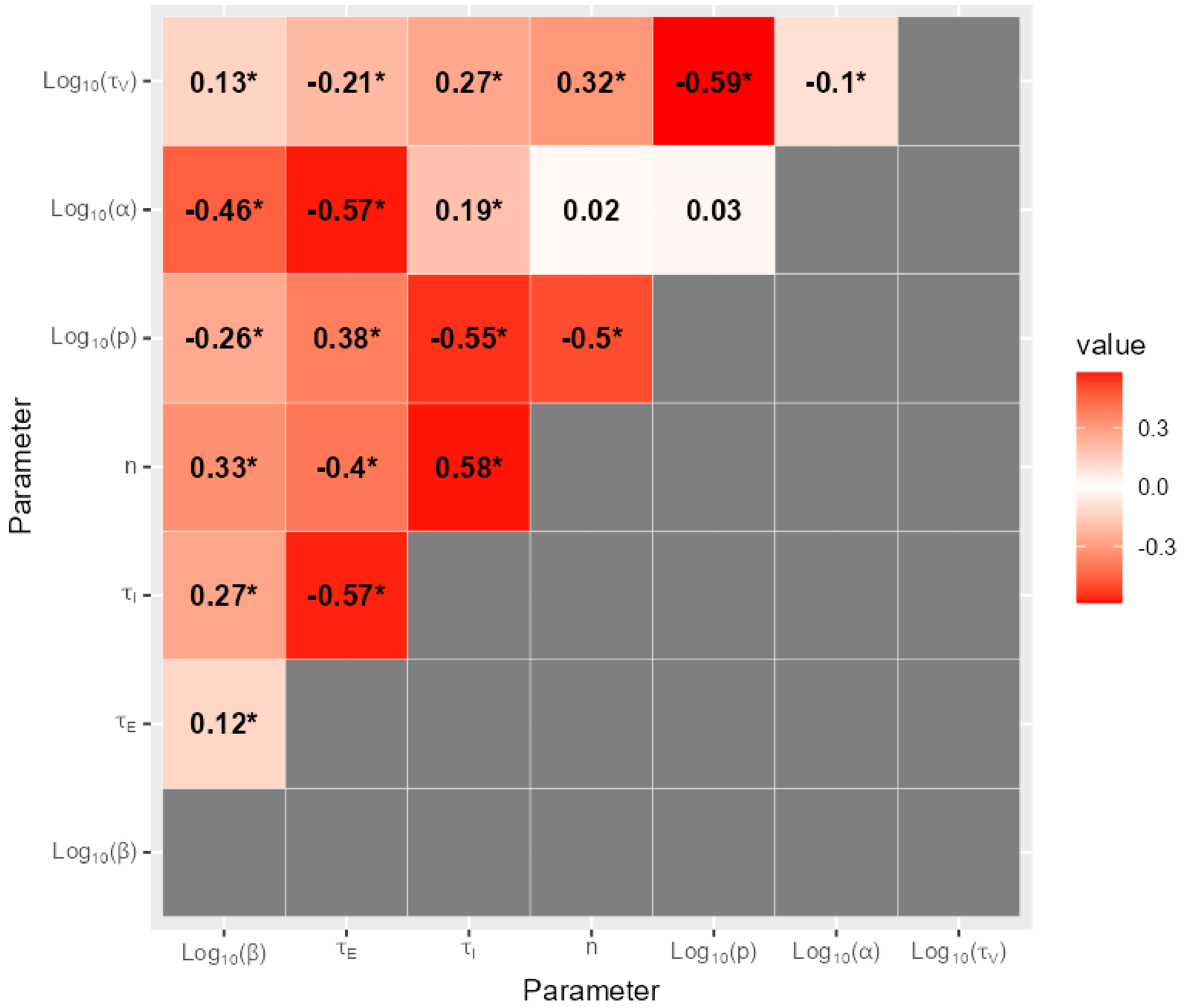

6.4 H5N1-AC on DEF cells

Figure S14. The shifts in the distributions of model parameters during the ABC-SMC analysis of data from strain H5N1-AC on DEF cells, when moving from the first particle population (containing accepted particles sampled from the prior distribution) to the posterior distribution via intermediate particle populations.

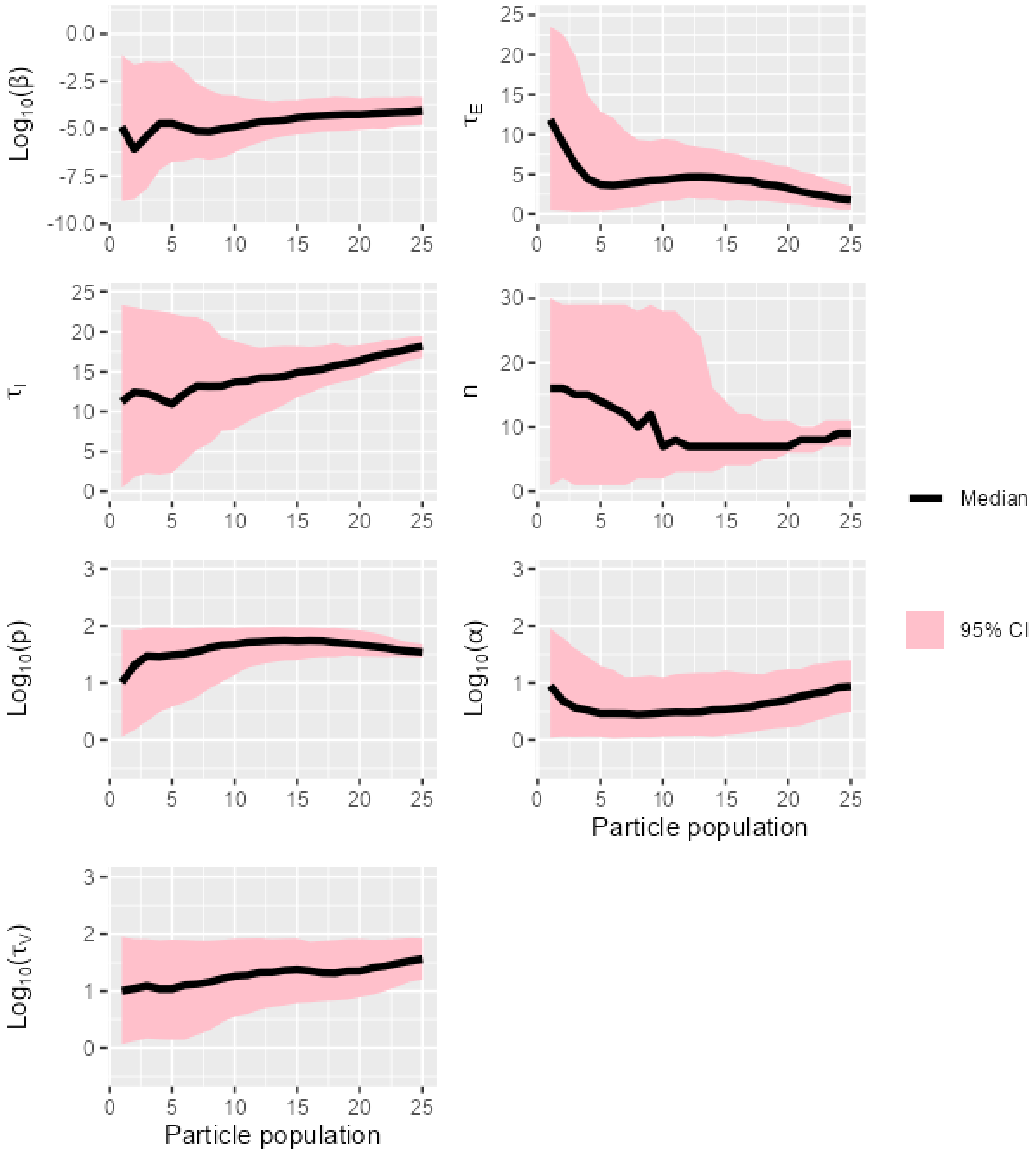

Figure S15. The overlap between prior and posterior distributions of model parameters for strain H5N1-AC on DEF cells.

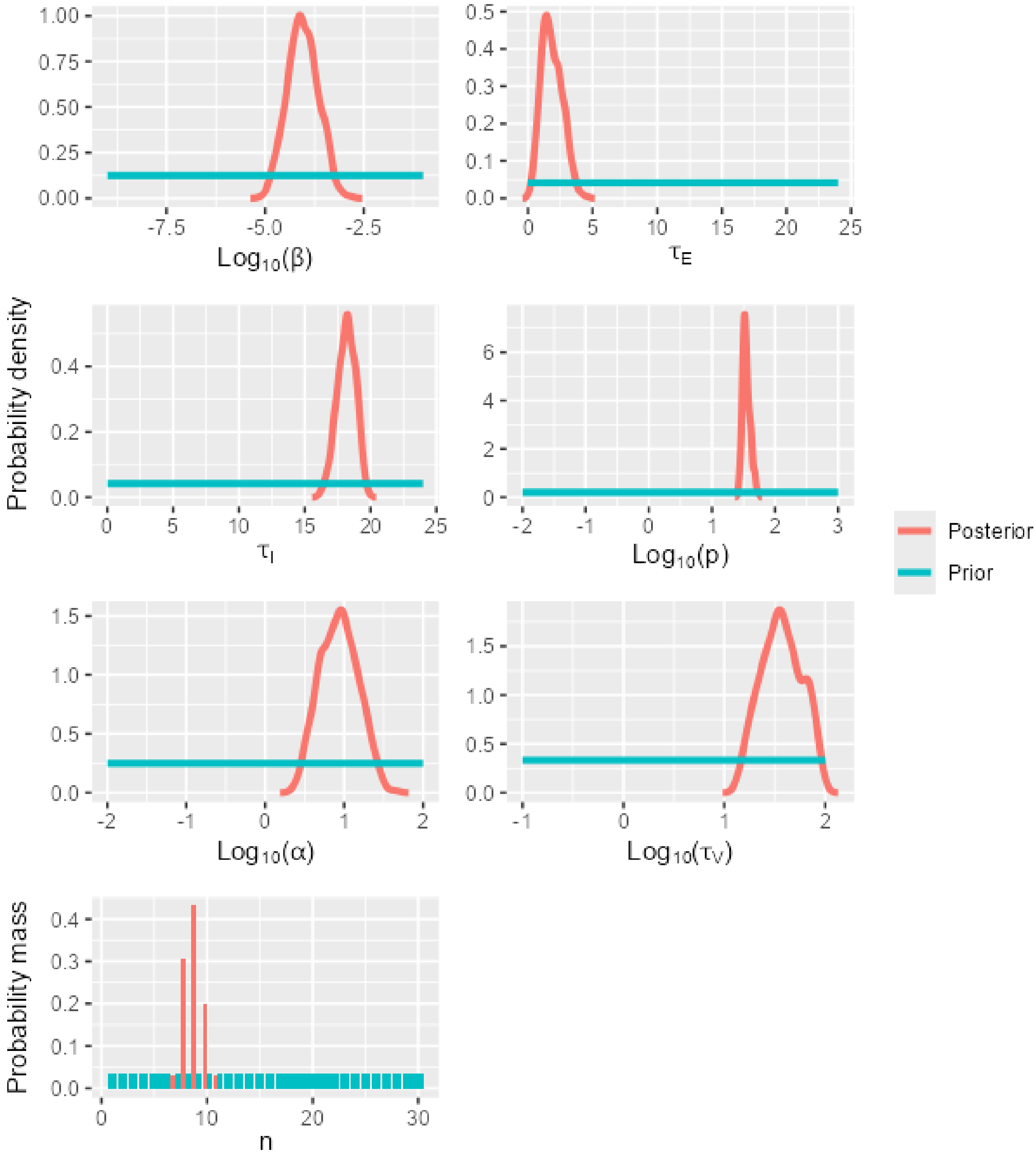

Figure S16. A matrix showing Kendall's rank correlation coefficient and the significance of the correlation (0.05 level, indicated by *) for pairs of model parameters calibrated using data for strain H5N1-AC on DEF cells.

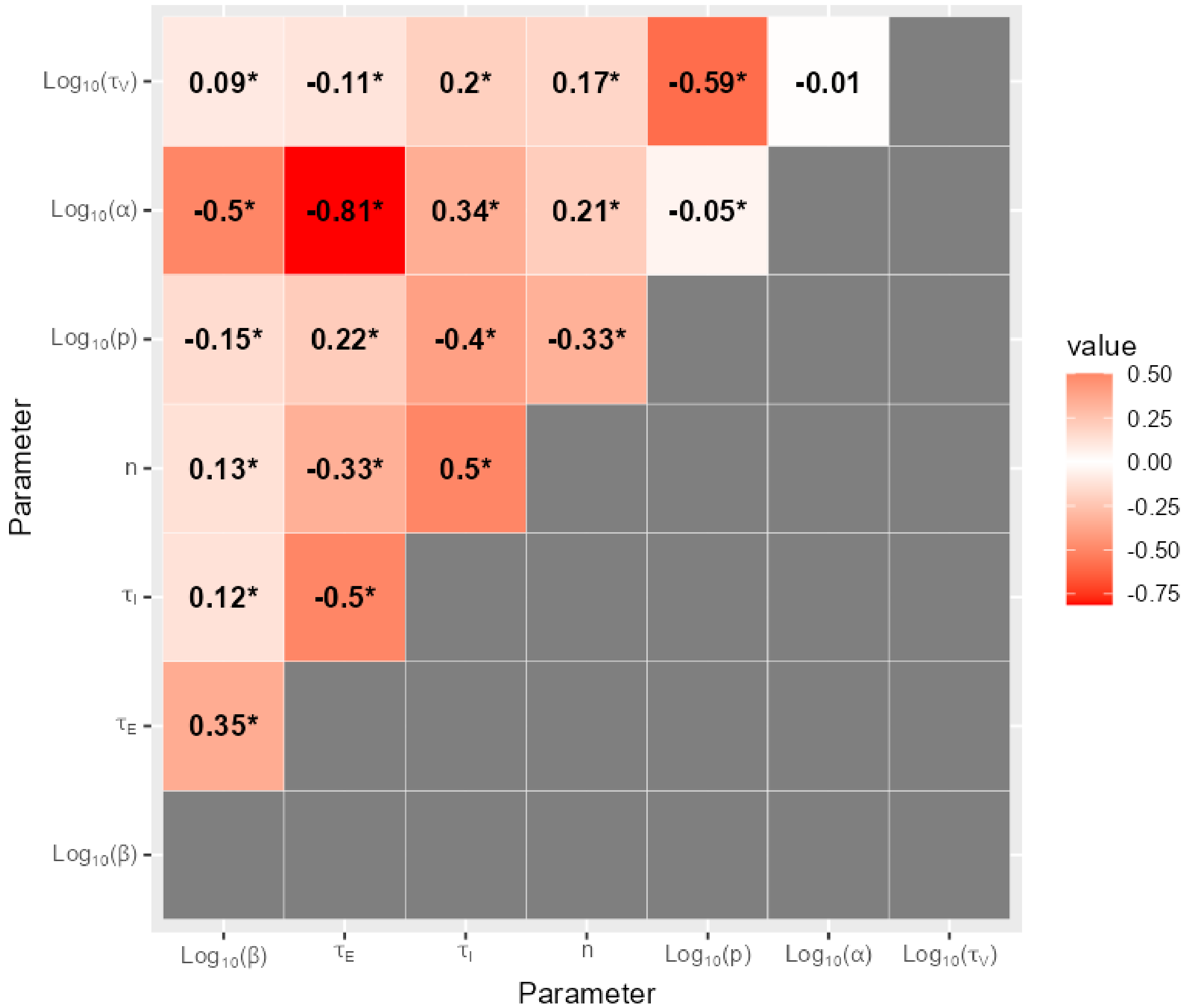

6.5 H5N1-BB on CEF cells

Figure S17. The shifts in the distributions of model parameters during the ABC-SMC analysis of data from strain H5N1-BB on CEF cells, when moving from the first particle population (containing accepted particles sampled from the prior distribution) to the posterior distribution via intermediate particle populations.

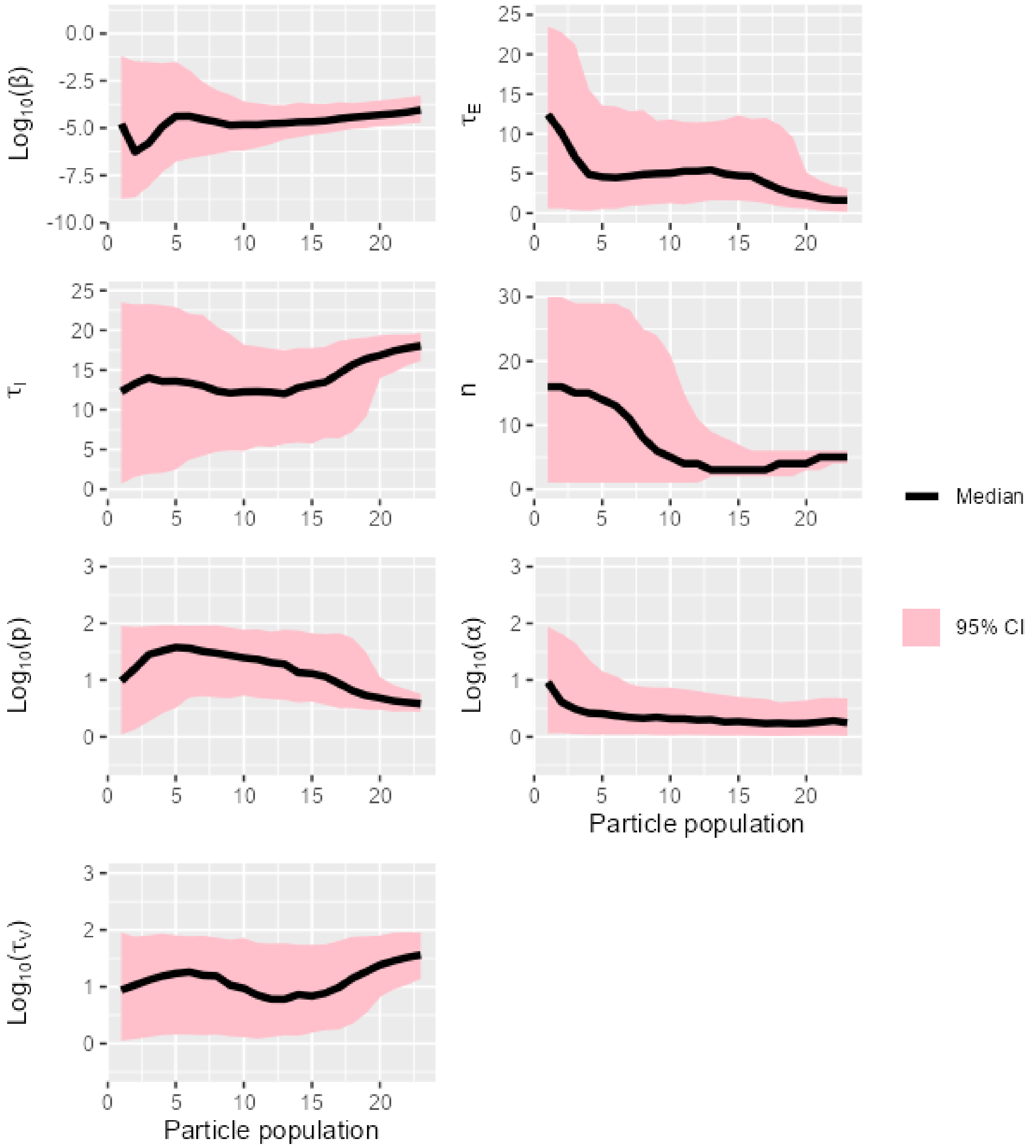

Figure S18. The overlap between prior and posterior distributions of model parameters for strain H5N1-BB on CEF cells.

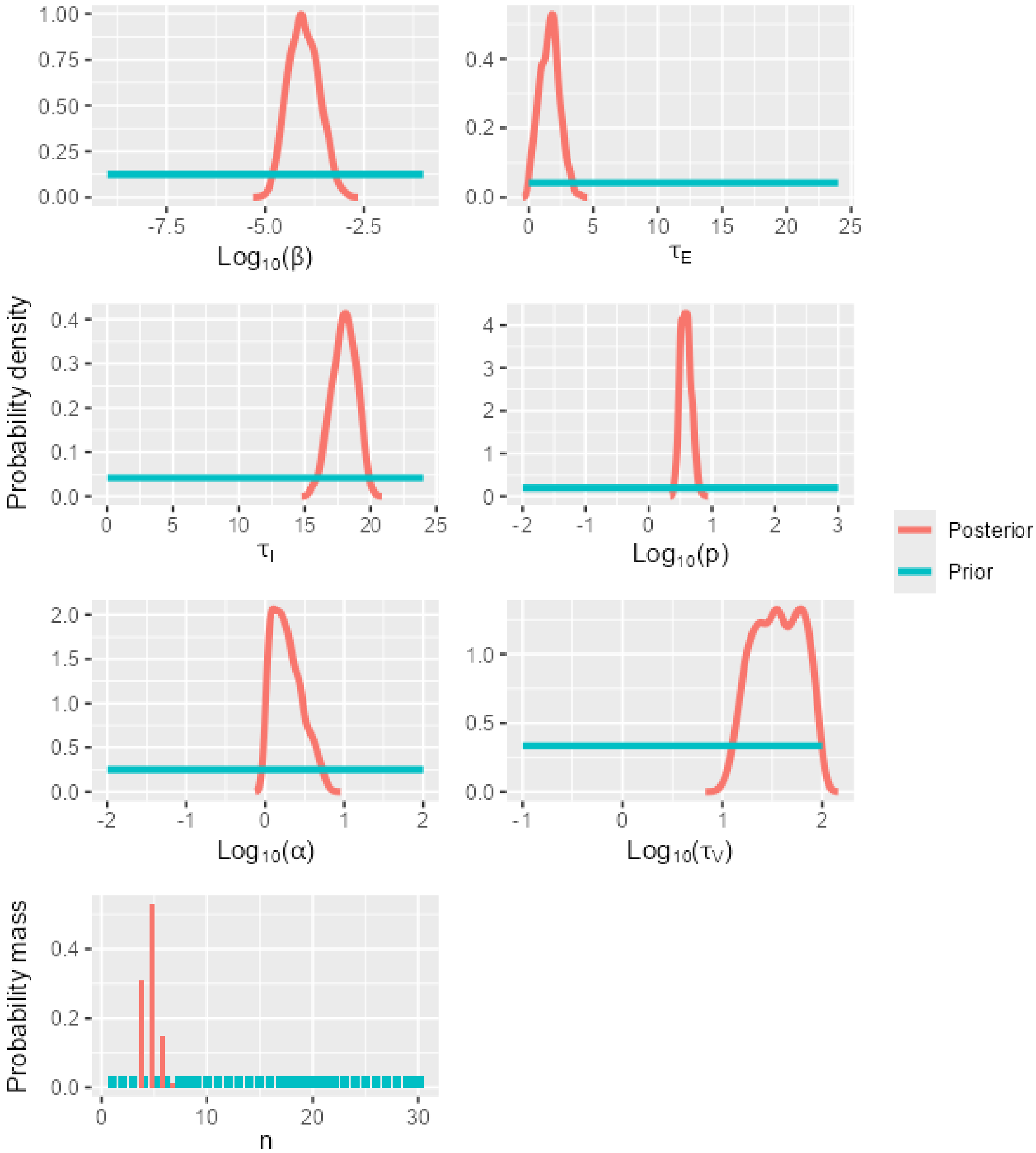

Figure S19. A matrix showing Kendall's rank correlation coefficient and the significance of the correlation (0.05 level, indicated by *) for pairs of model parameters calibrated using data for strain H5N1-BB on CEF cells.

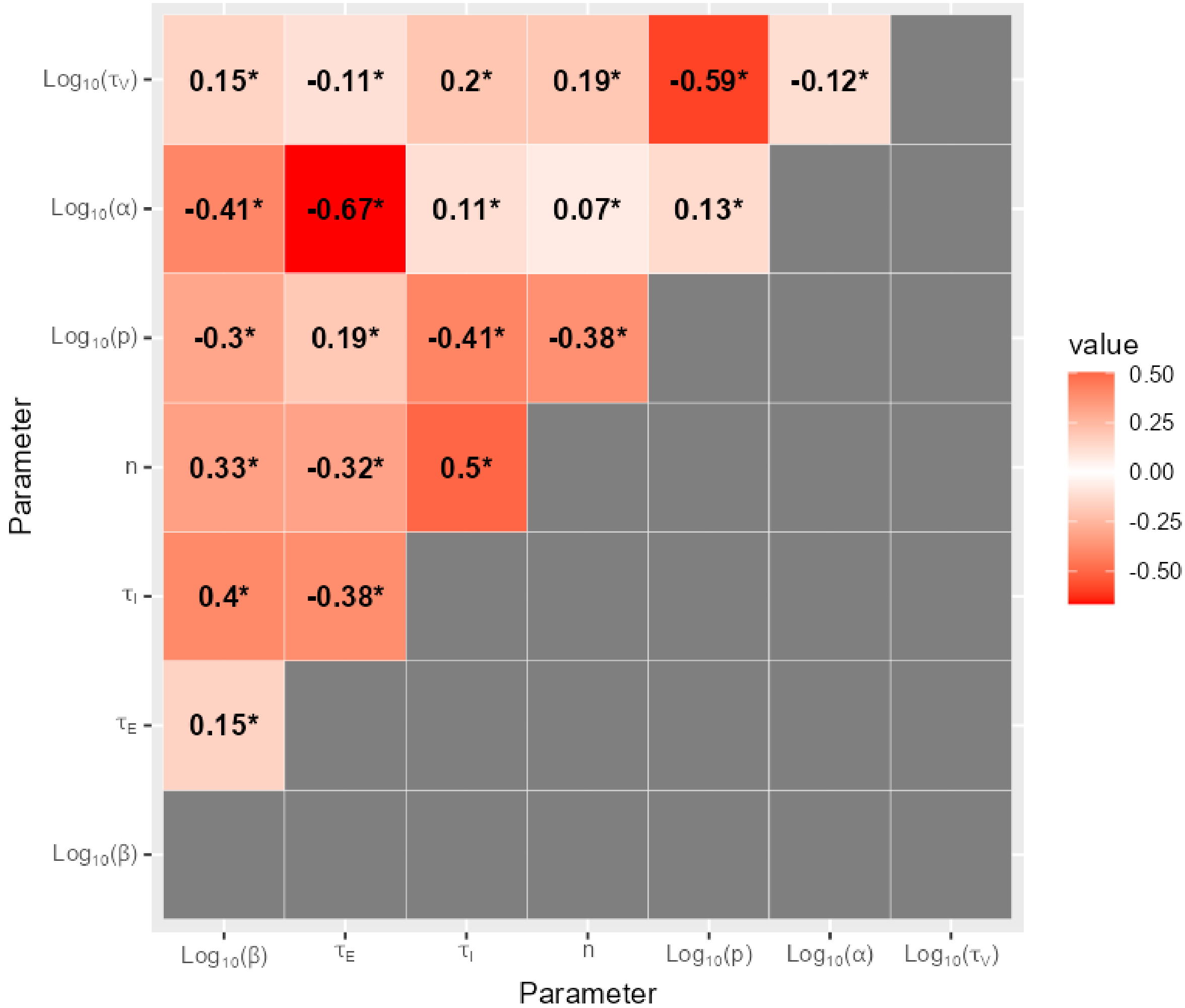

6.6 H5N1-BB on DEF cells

Figure S20. The shifts in the distributions of model parameters during the ABC-SMC analysis of data from strain H5N1-BB on DEF cells, when moving from the first particle population (containing accepted particles sampled from the prior distribution) to the posterior distribution via intermediate particle populations.

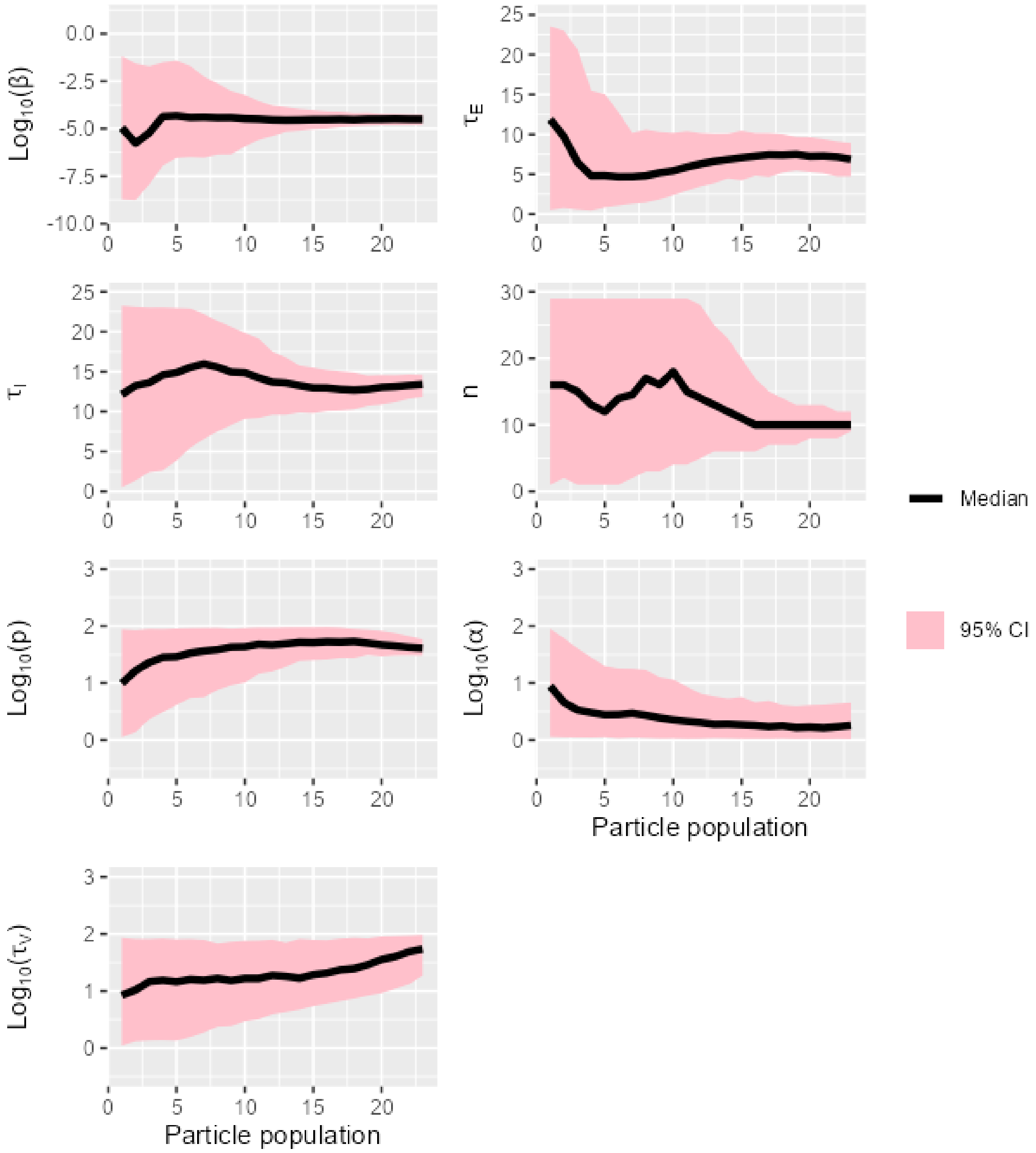

Figure S21. The overlap between prior and posterior distributions of model parameters for strain H5N1-BB on DEF cells.

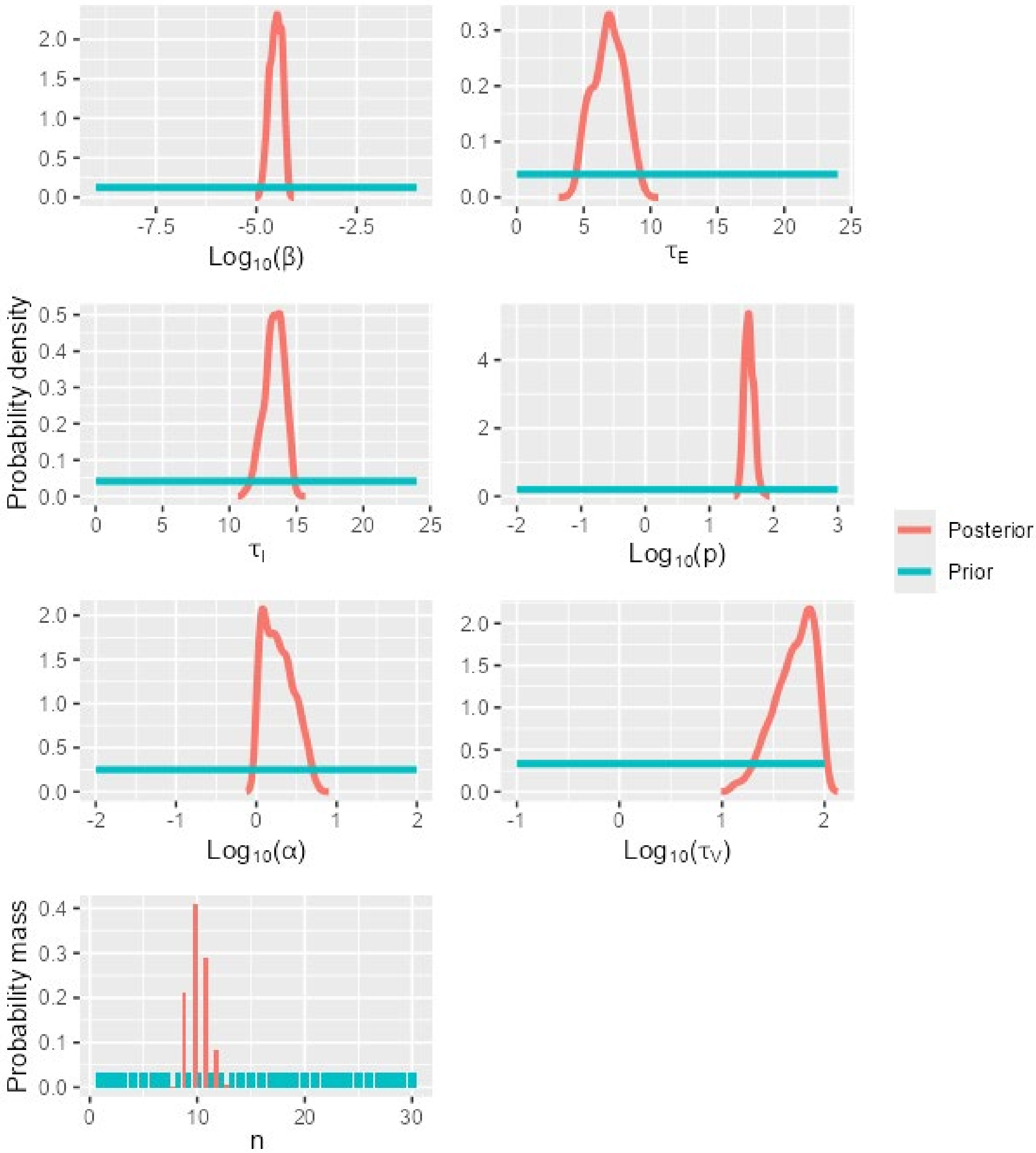

Figure S22. A matrix showing Kendall's rank correlation coefficient and the significance of the correlation (0.05 level, indicated by *) for pairs of model parameters calibrated using data for strain H5N1-BB on DEF cells.

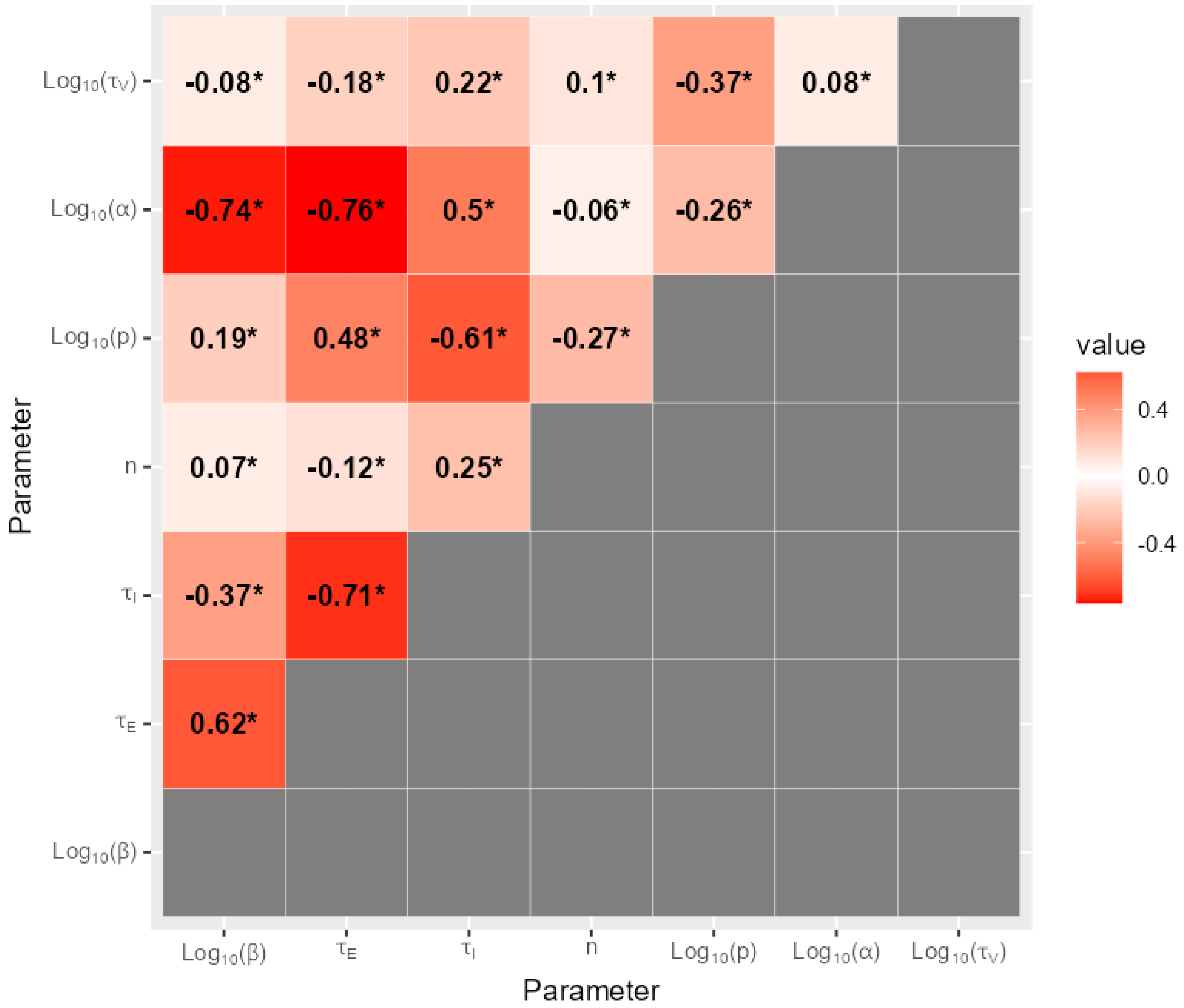

6.7 H5N1-C on CEF cells

Figure S23. The shifts in the distributions of model parameters during the ABC-SMC analysis of data from strain H5N1-C on CEF cells, when moving from the first particle population (containing accepted particles sampled from the prior distribution) to the posterior distribution via intermediate particle populations.

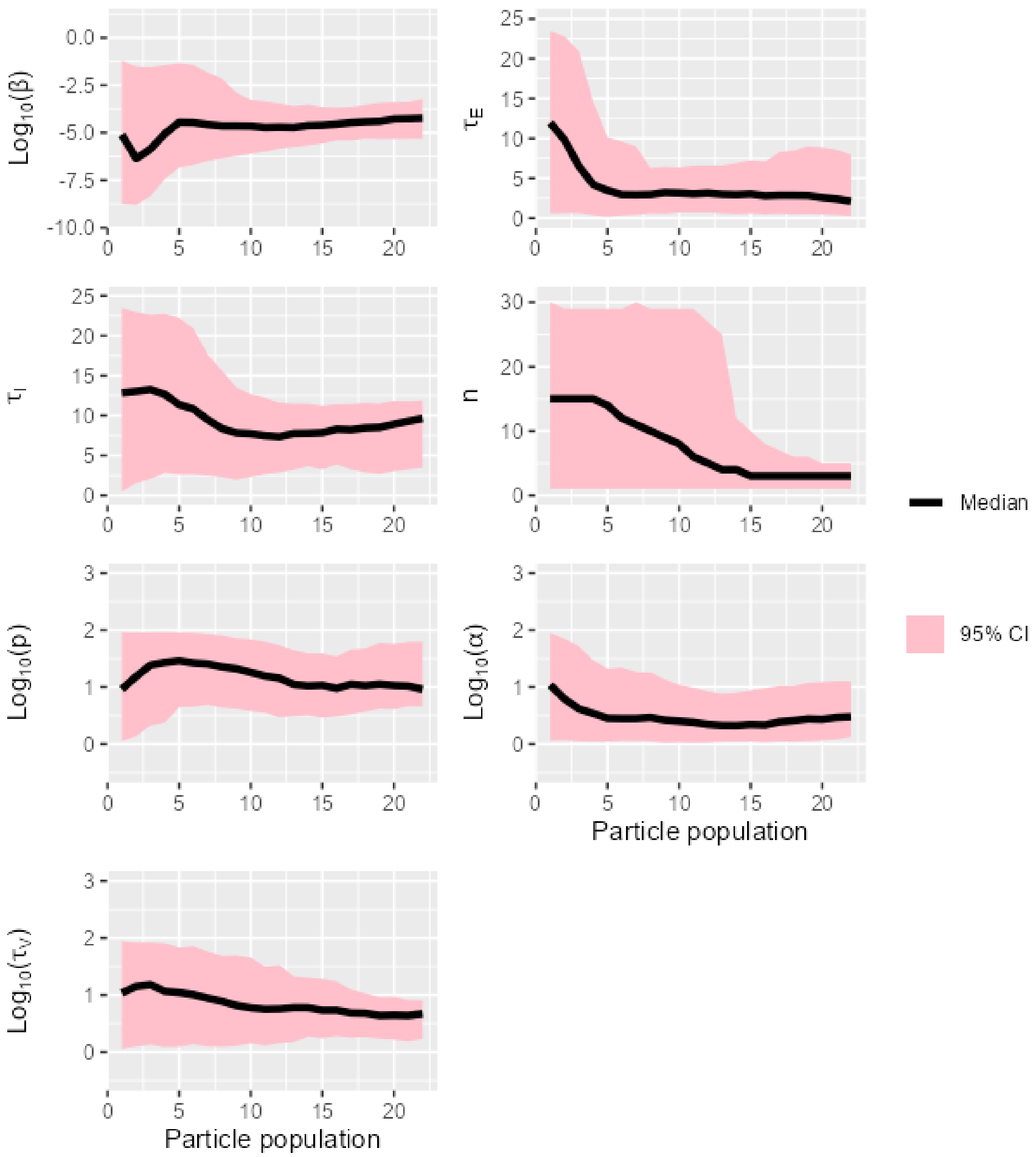

Figure S24. The overlap between prior and posterior distributions of model parameters for strain H5N1-C on CEF cells.

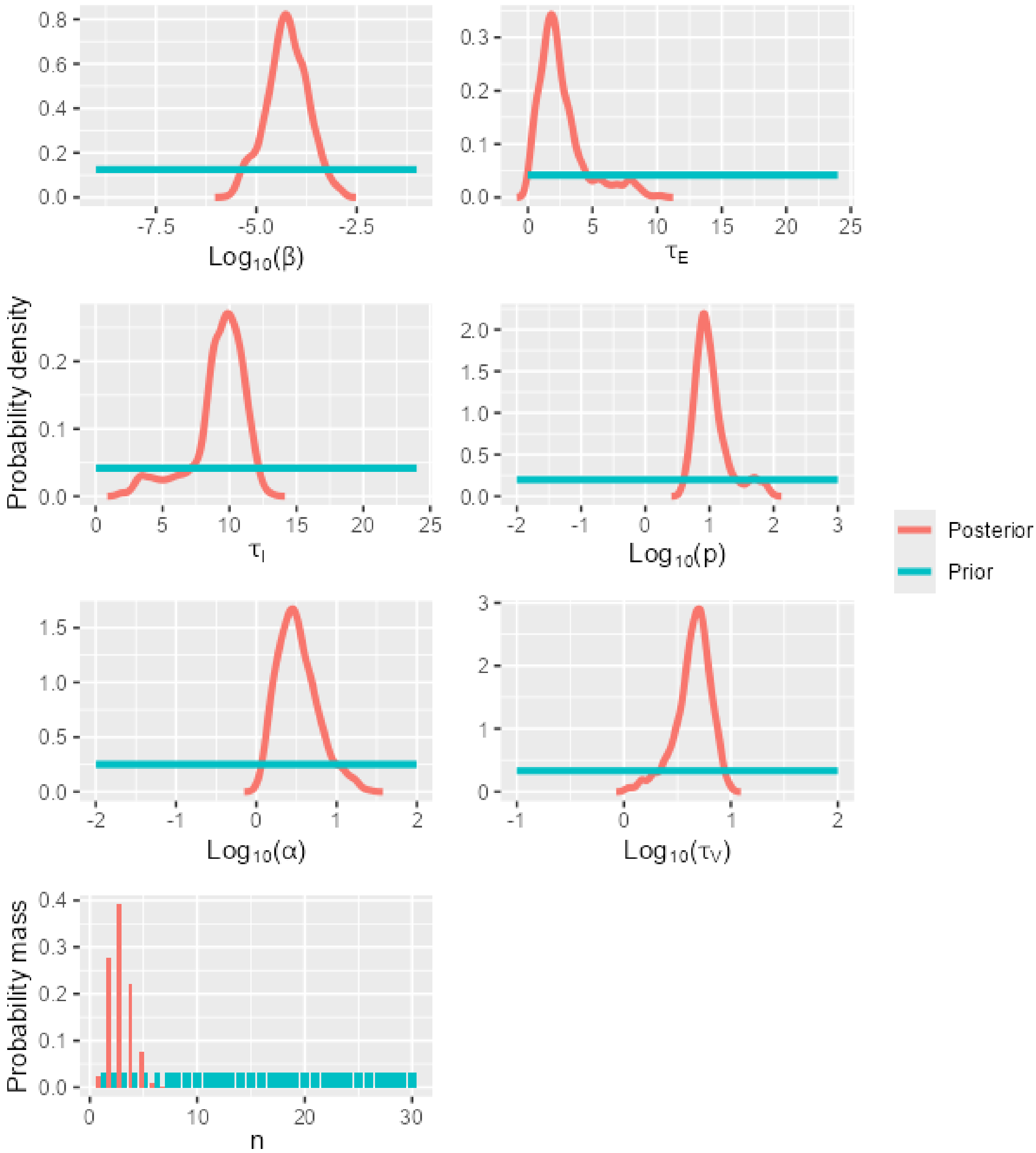

Figure S25. A matrix showing Kendall's rank correlation coefficient and the significance of the correlation (0.05 level, indicated by *) for pairs of model parameters calibrated using data for strain H5N1-C on CEF cells.

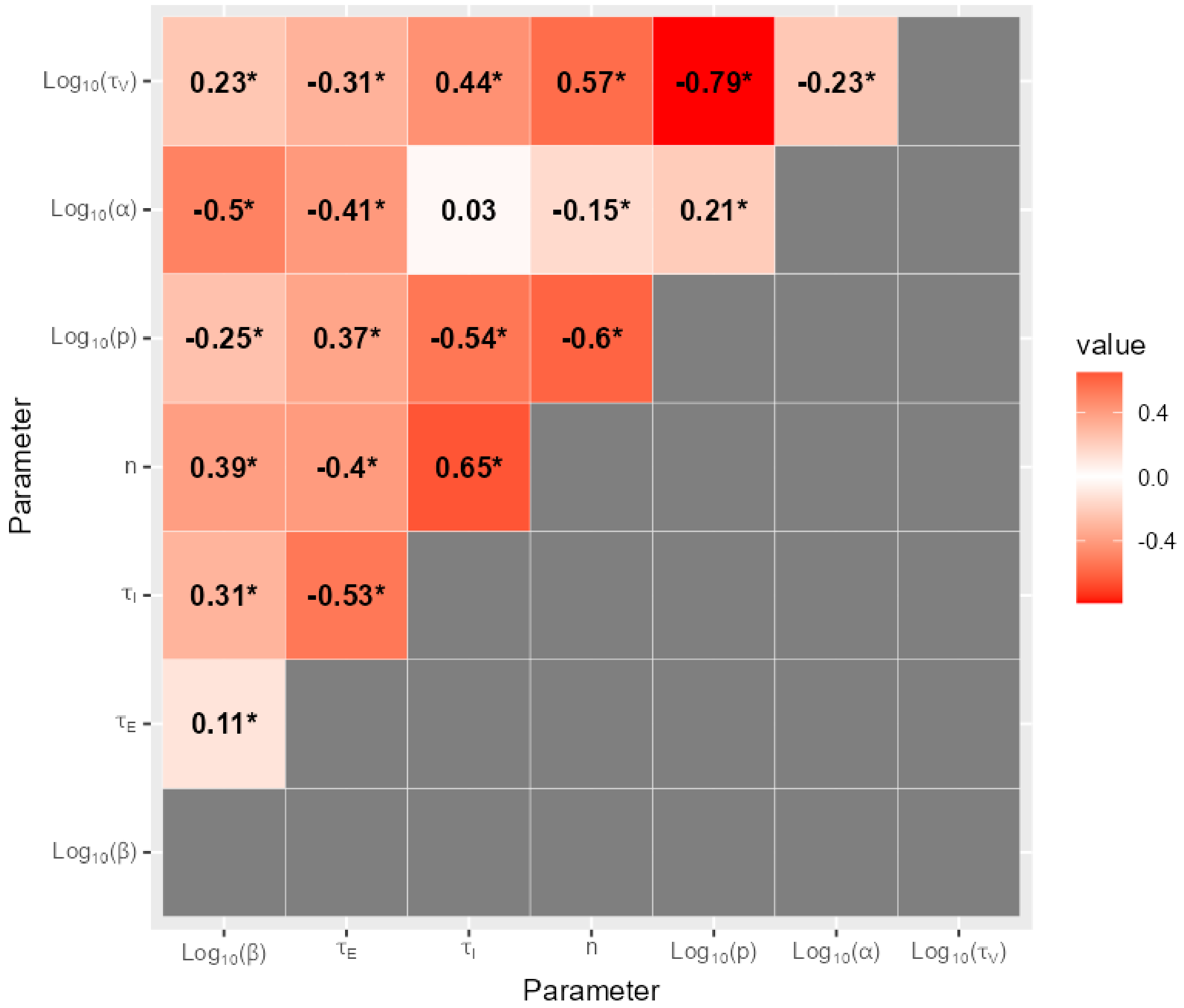

6.8 H5N1-C on DEF cells

Figure S26. The shifts in the distributions of model parameters during the ABC-SMC analysis of data from strain H5N1-C on DEF cells, when moving from the first particle population (containing accepted particles sampled from the prior distribution) to the posterior distribution via intermediate particle populations.

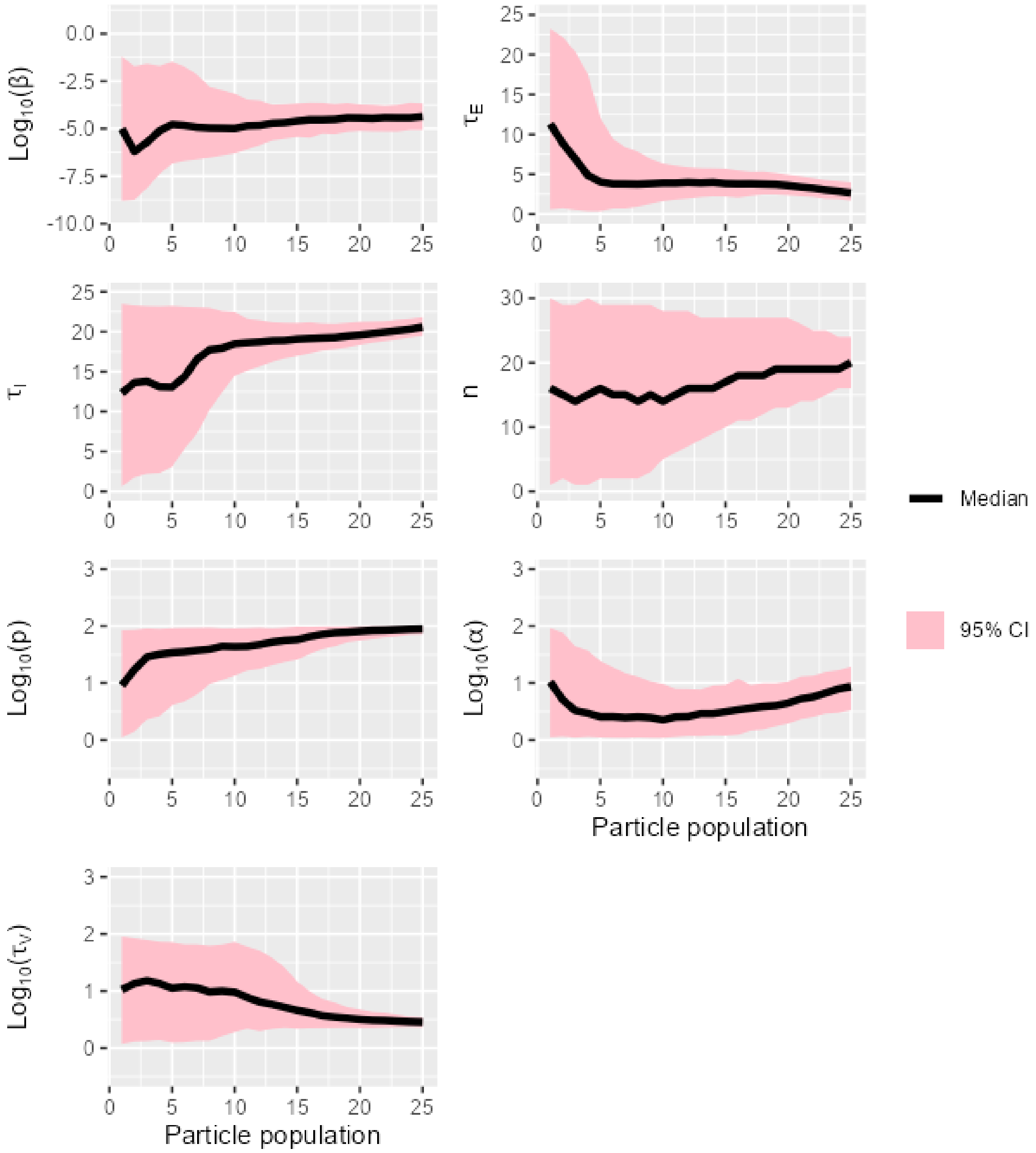

Figure S27. The overlap between prior and posterior distributions of model parameters for strain H5N1-C on DEF cells.

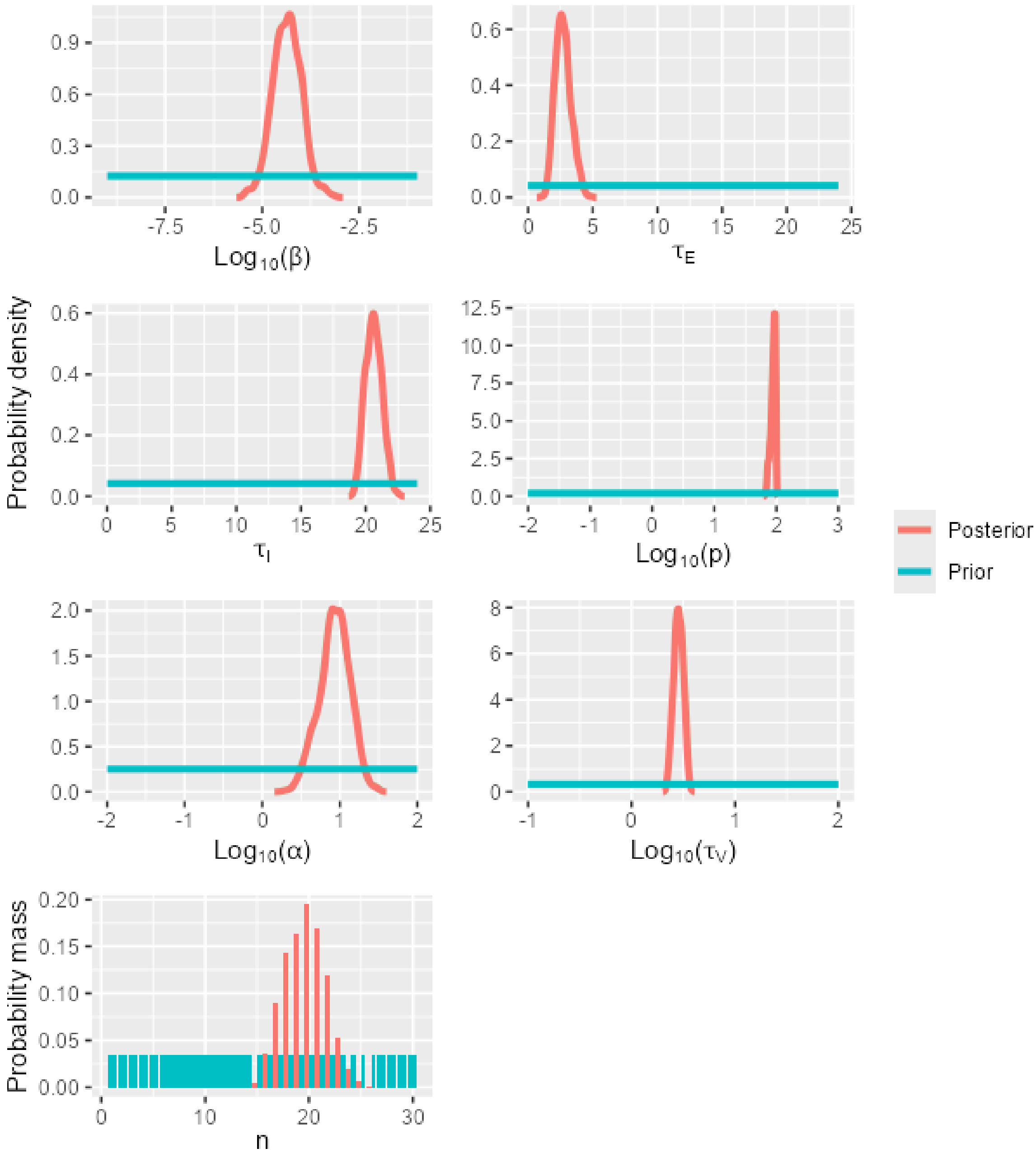

Figure S28. A matrix showing Kendall's rank correlation coefficient and the significance of the correlation (0.05 level, indicated by *) for pairs of model parameters calibrated using data for strain H5N1-C on DEF cells.

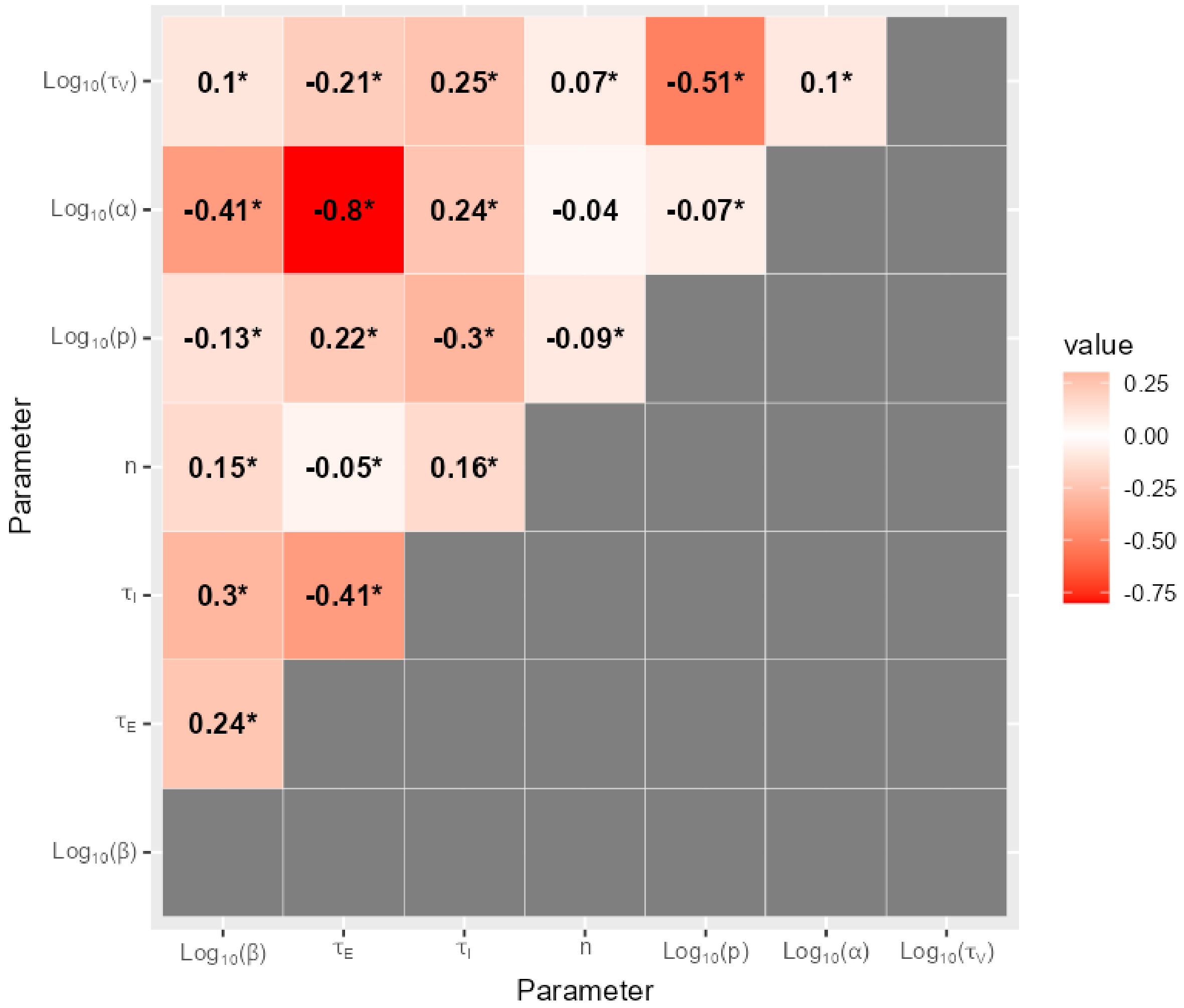

6.9 H5N6-2017 on CEF cells

Figure S29. The shifts in the distributions of model parameters during the ABC-SMC analysis of data from strain H5N6-2017 on CEF cells, when moving from the first particle population (containing accepted particles sampled from the prior distribution) to the posterior distribution via intermediate particle populations.

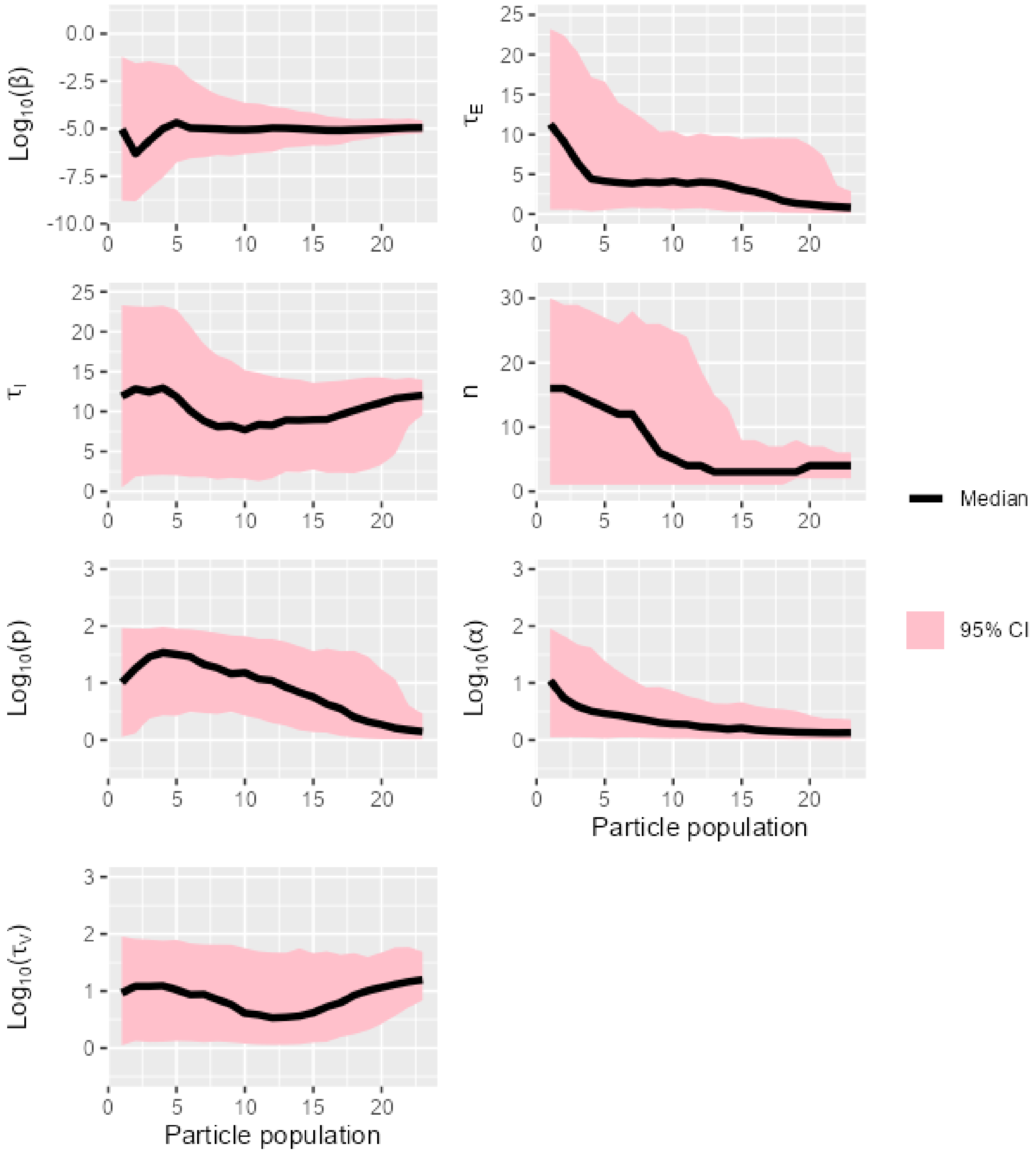

Figure S30. The overlap between prior and posterior distributions of model parameters for strain H5N6-2017 on CEF cells.

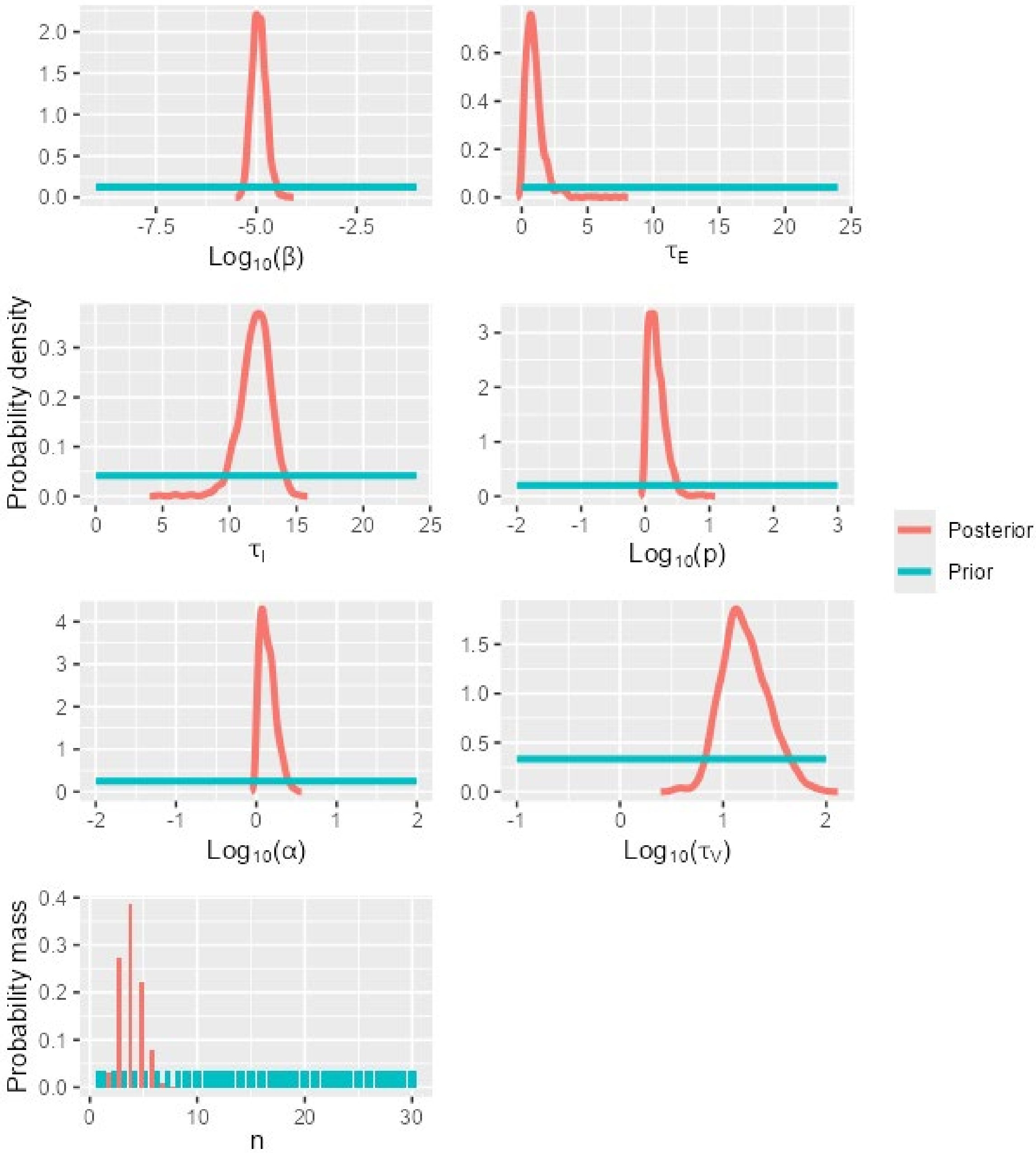

Figure S31. A matrix showing Kendall's rank correlation coefficient and the significance of the correlation (0.05 level, indicated by *) for pairs of model parameters calibrated using data for strain H5N6-2017 on CEF cells.

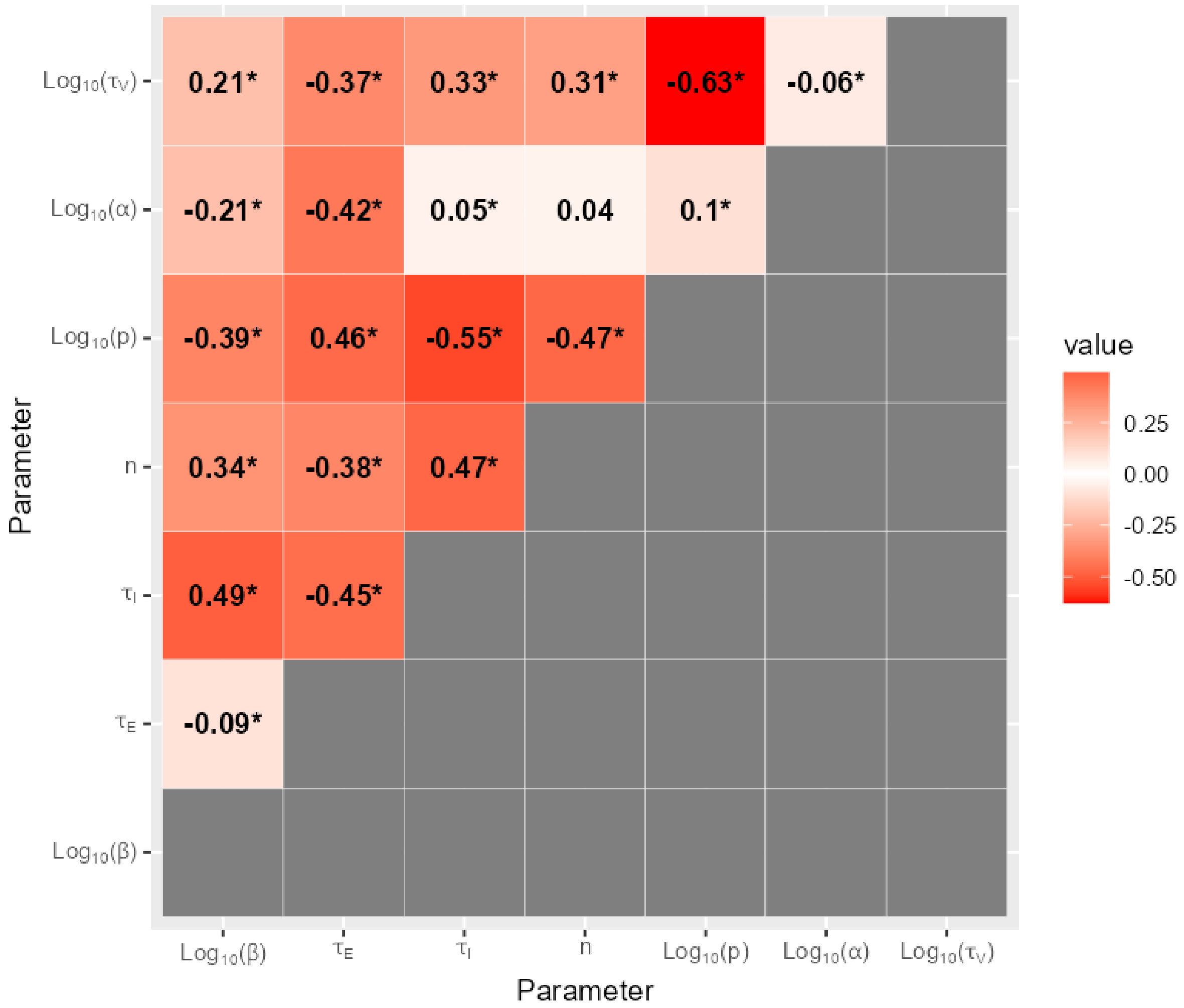

6.10 H5N6-2017 on DEF cells

Figure S32. The shifts in the distributions of model parameters during the ABC-SMC analysis of data from strain H5N6-2017 on DEF cells, when moving from the first particle population (containing accepted particles sampled from the prior distribution) to the posterior distribution via intermediate particle populations.

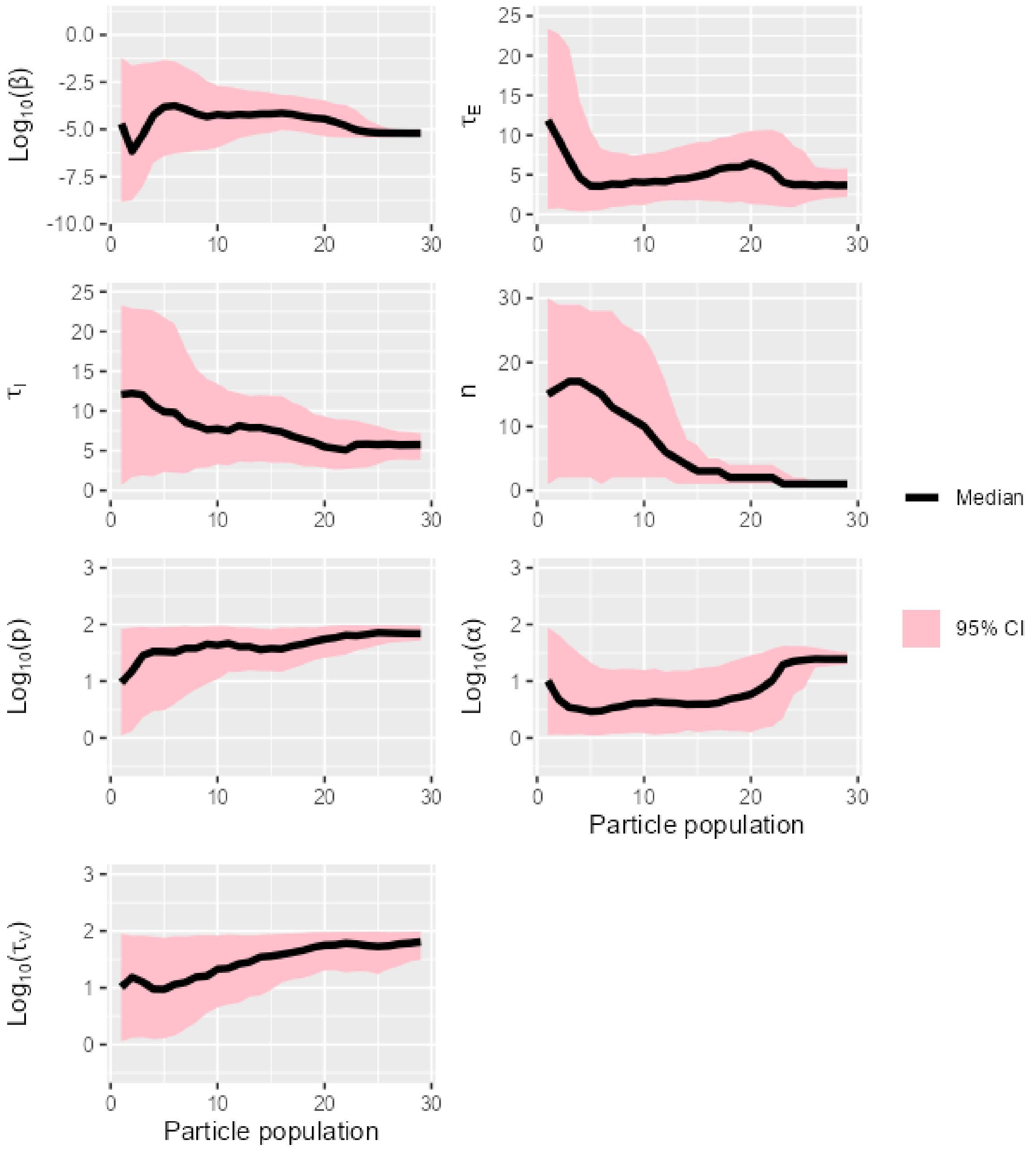

Figure S33. The overlap between prior and posterior distributions of model parameters for strain H5N6-2017 on DEF cells.

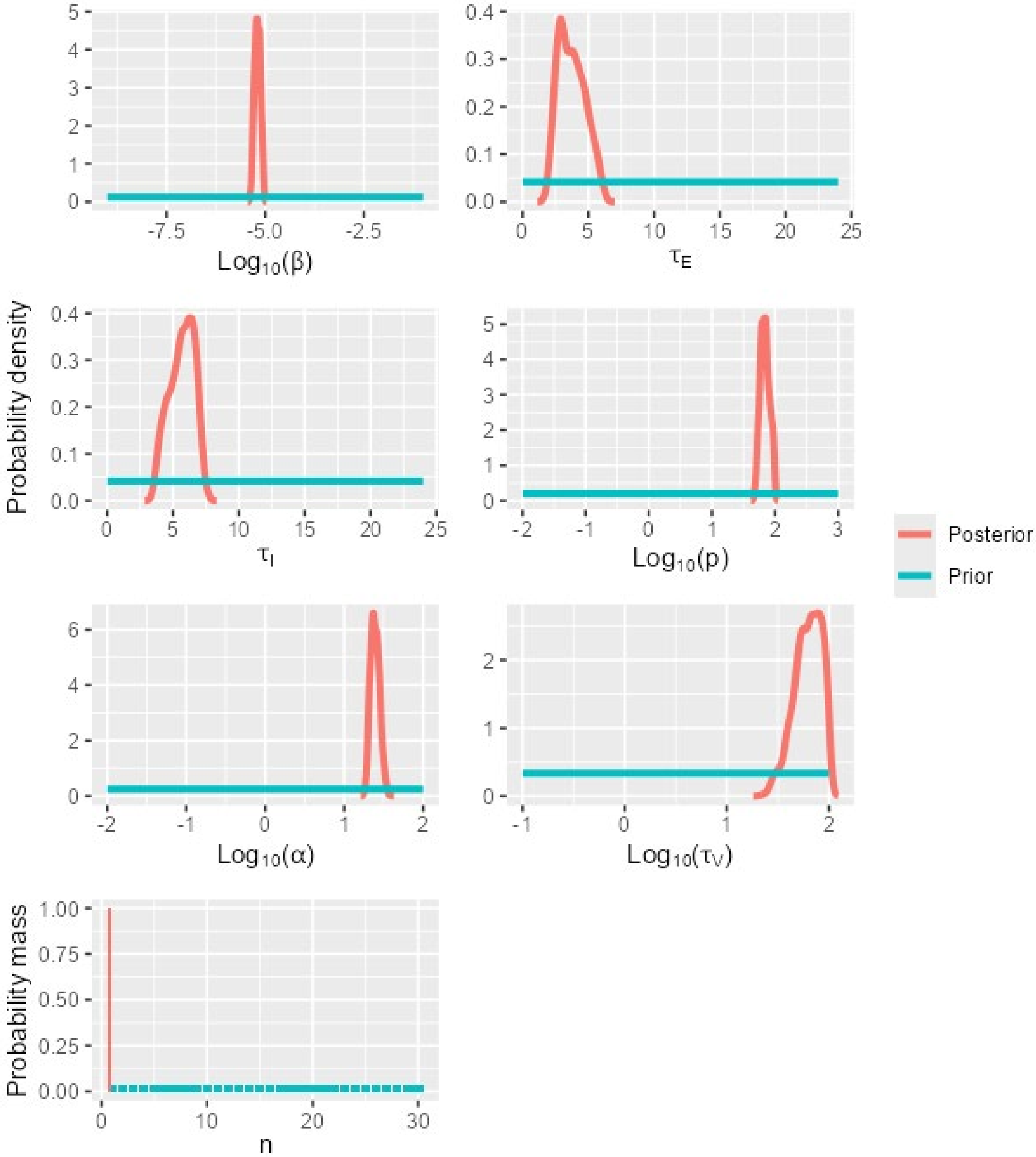

Figure S34. A matrix showing Kendall's rank correlation coefficient and the significance of the correlation (0.05 level, indicated by *) for pairs of model parameters calibrated using data for strain H5N6-2017 on DEF cells.

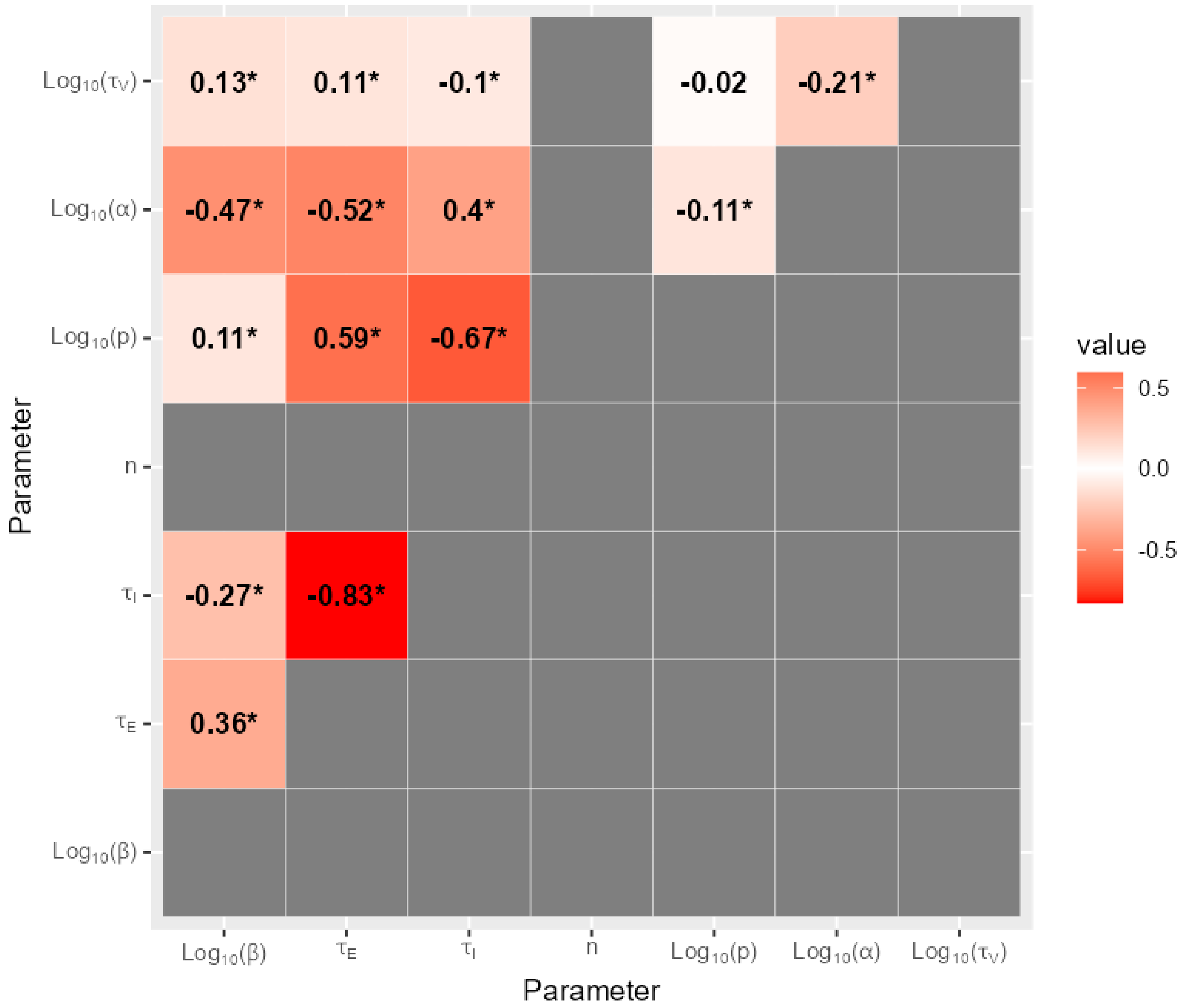

6.11 H5N8-2014 on CEF cells

Figure S35. The shifts in the distributions of model parameters during the ABC-SMC analysis of data from strain H5N8-2014 on CEF cells, when moving from the first particle population (containing accepted particles sampled from the prior distribution) to the posterior distribution via intermediate particle populations.

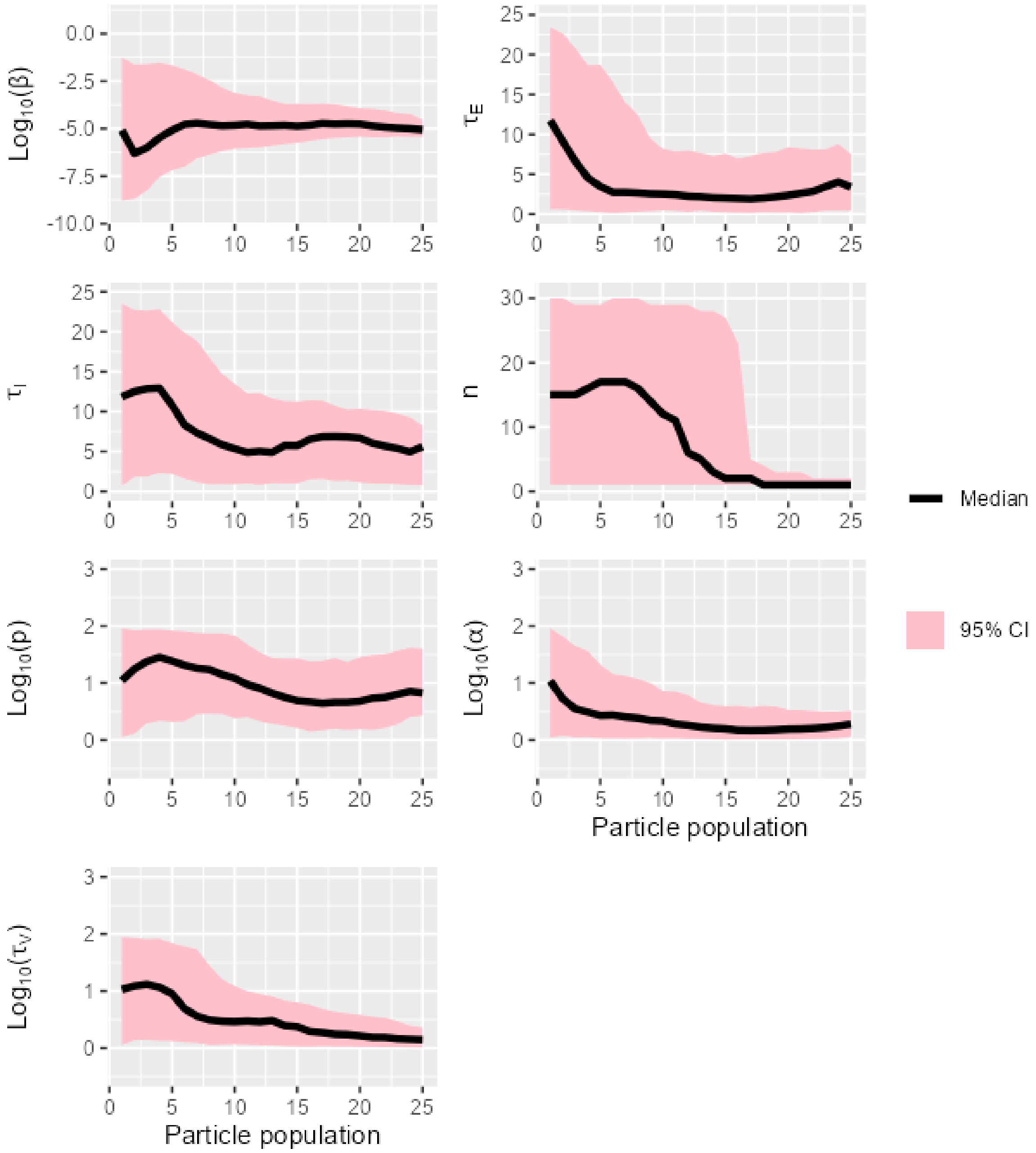

Figure S36. The overlap between prior and posterior distributions of model parameters for strain H5N8-2014 on CEF cells.

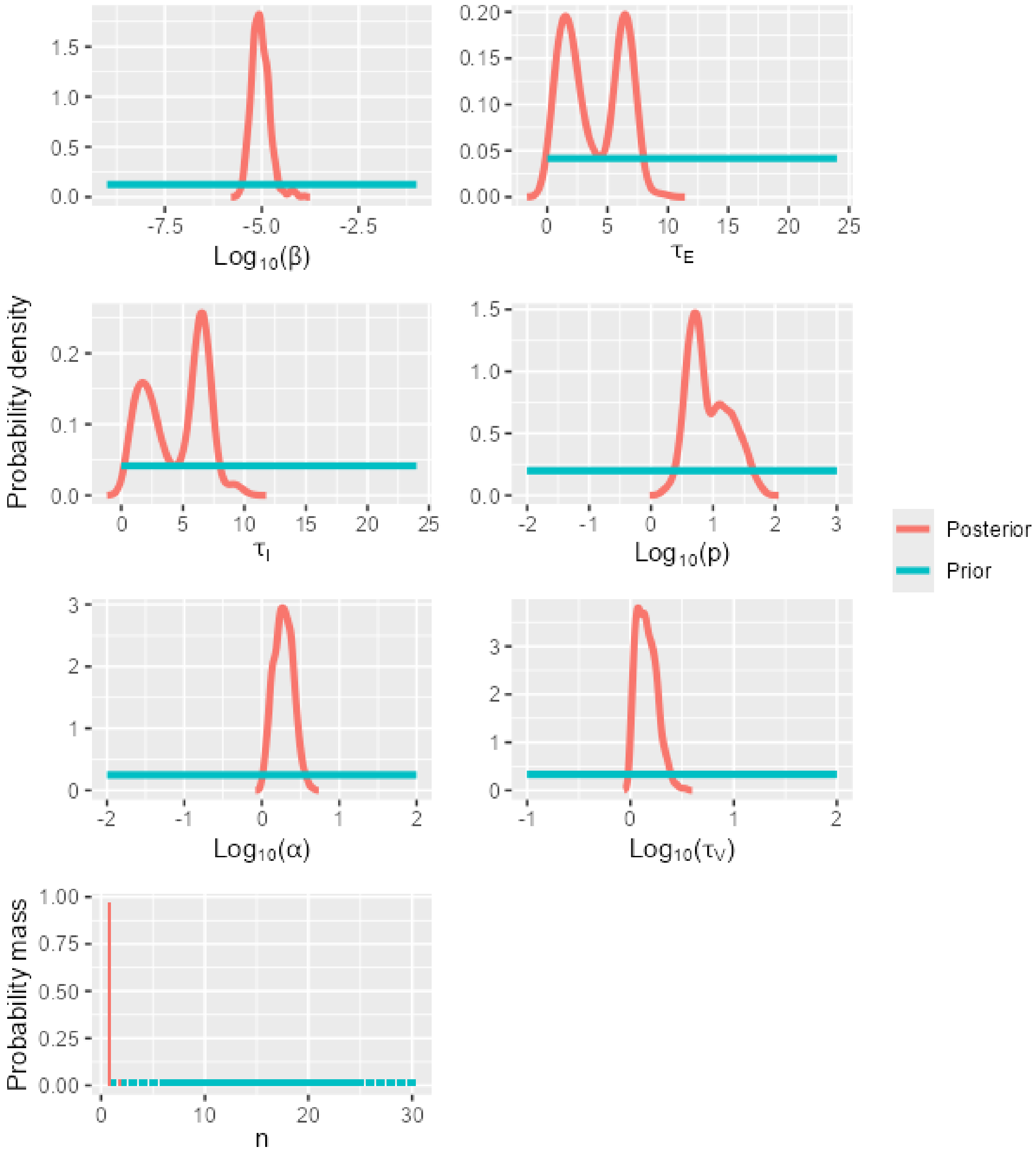

Figure S37. A matrix showing Kendall's rank correlation coefficient and the significance of the correlation (0.05 level, indicated by *) for pairs of model parameters calibrated using data for strain H5N8-2016 on CEF cells.

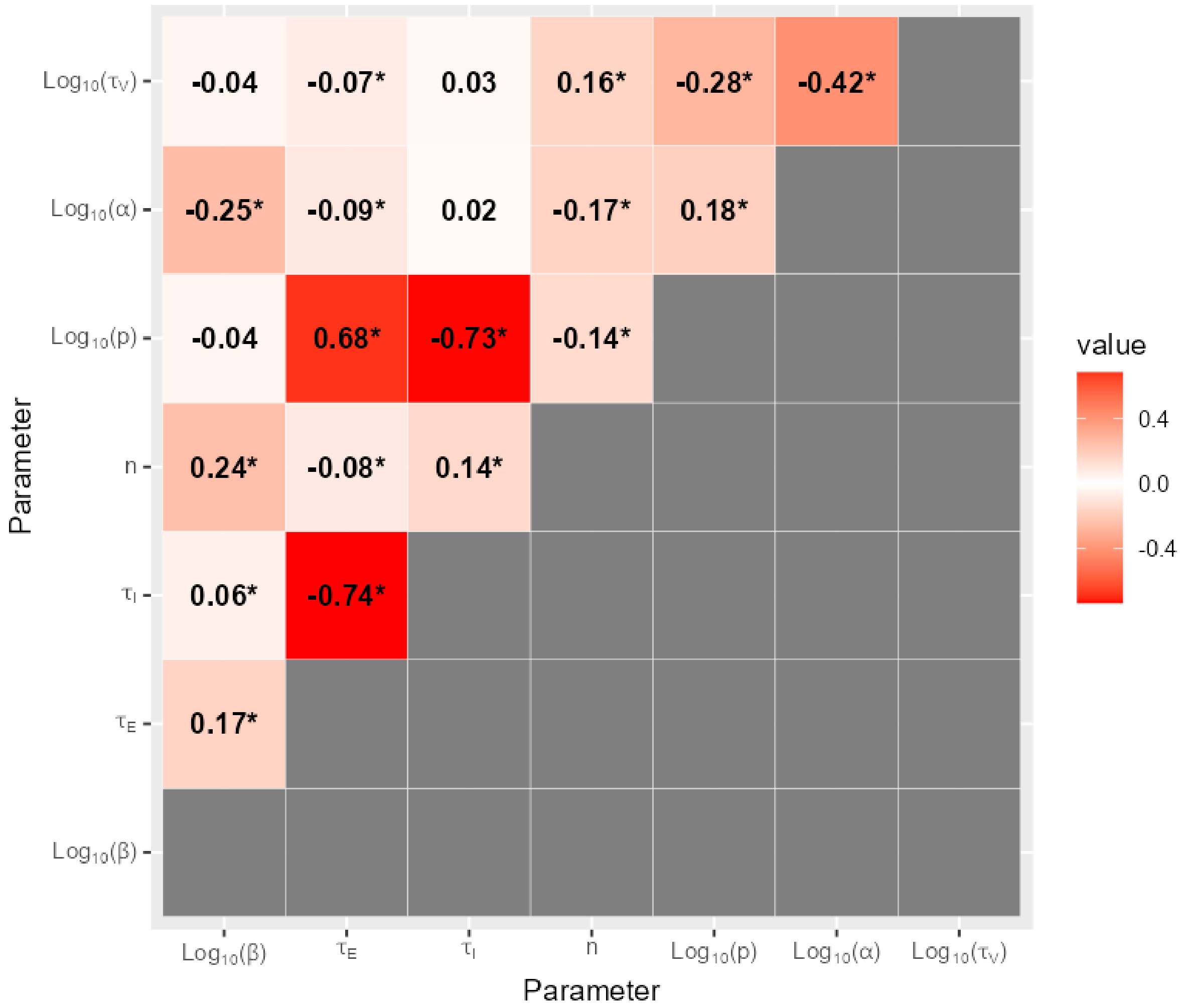

6.12 H5N8-2014 on DEF cells

Figure S38. The shifts in the distributions of model parameters during the ABC-SMC analysis of data from strain H5N8-2014 on DEF cells, when moving from the first particle population (containing accepted particles sampled from the prior distribution) to the posterior distribution via intermediate particle populations.

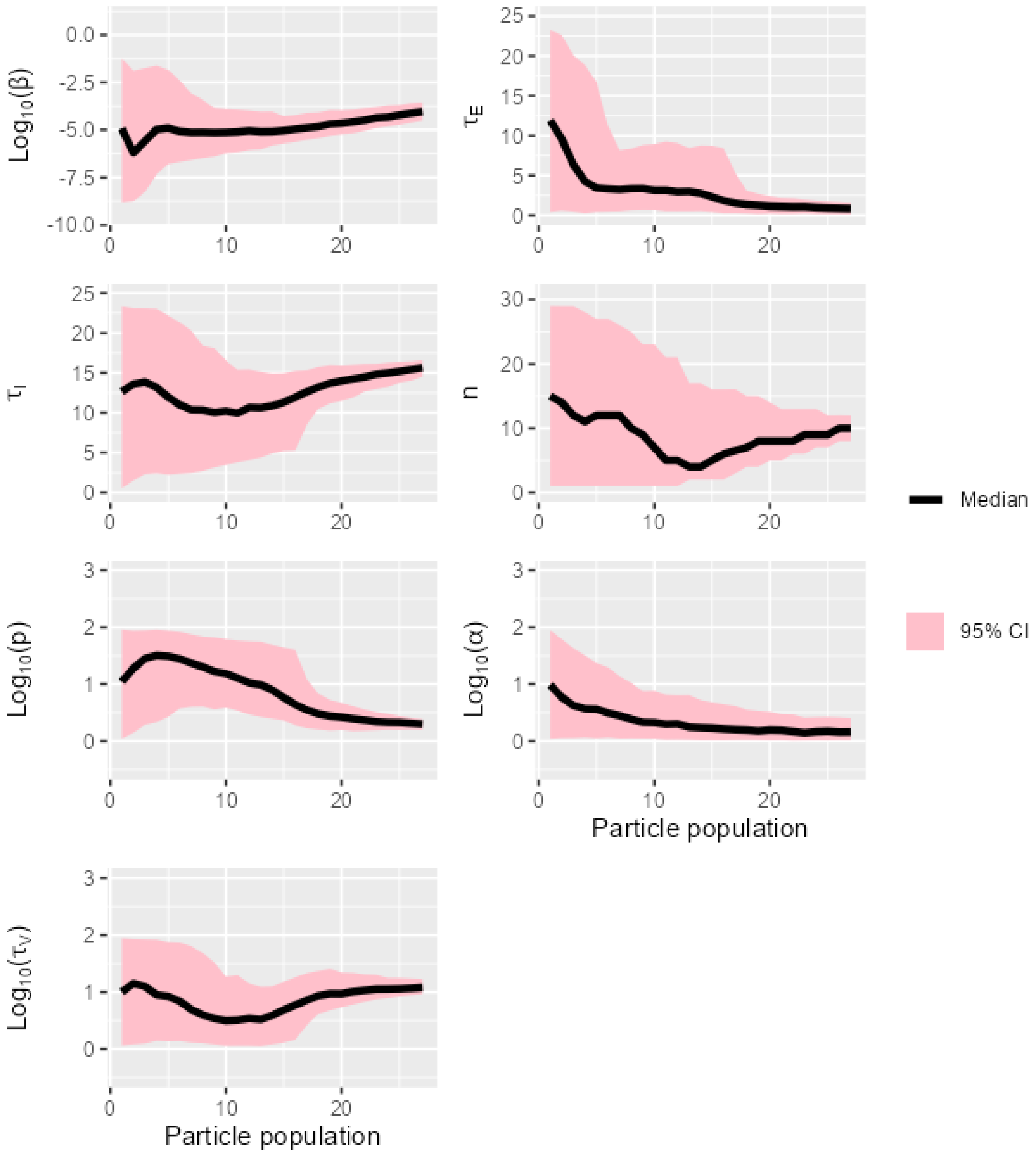

Figure S39. The overlap between prior and posterior distributions of model parameters for strain H5N8-2014 on DEF cells.

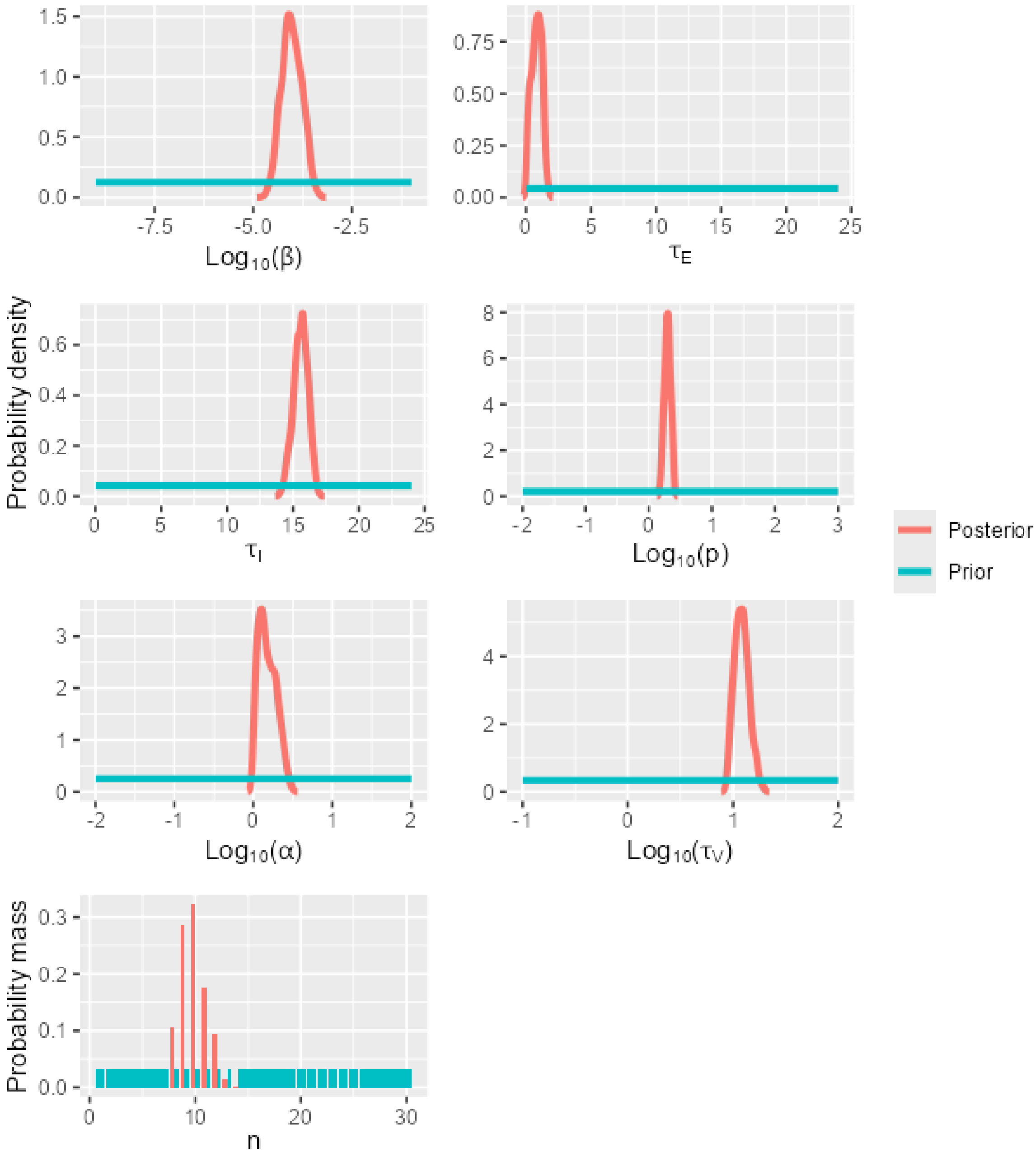

Figure S40. A matrix showing Kendall's rank correlation coefficient and the significance of the correlation (0.05 level, indicated by *) for pairs of model parameters calibrated using data for strain H5N8-2014 on DEF cells.

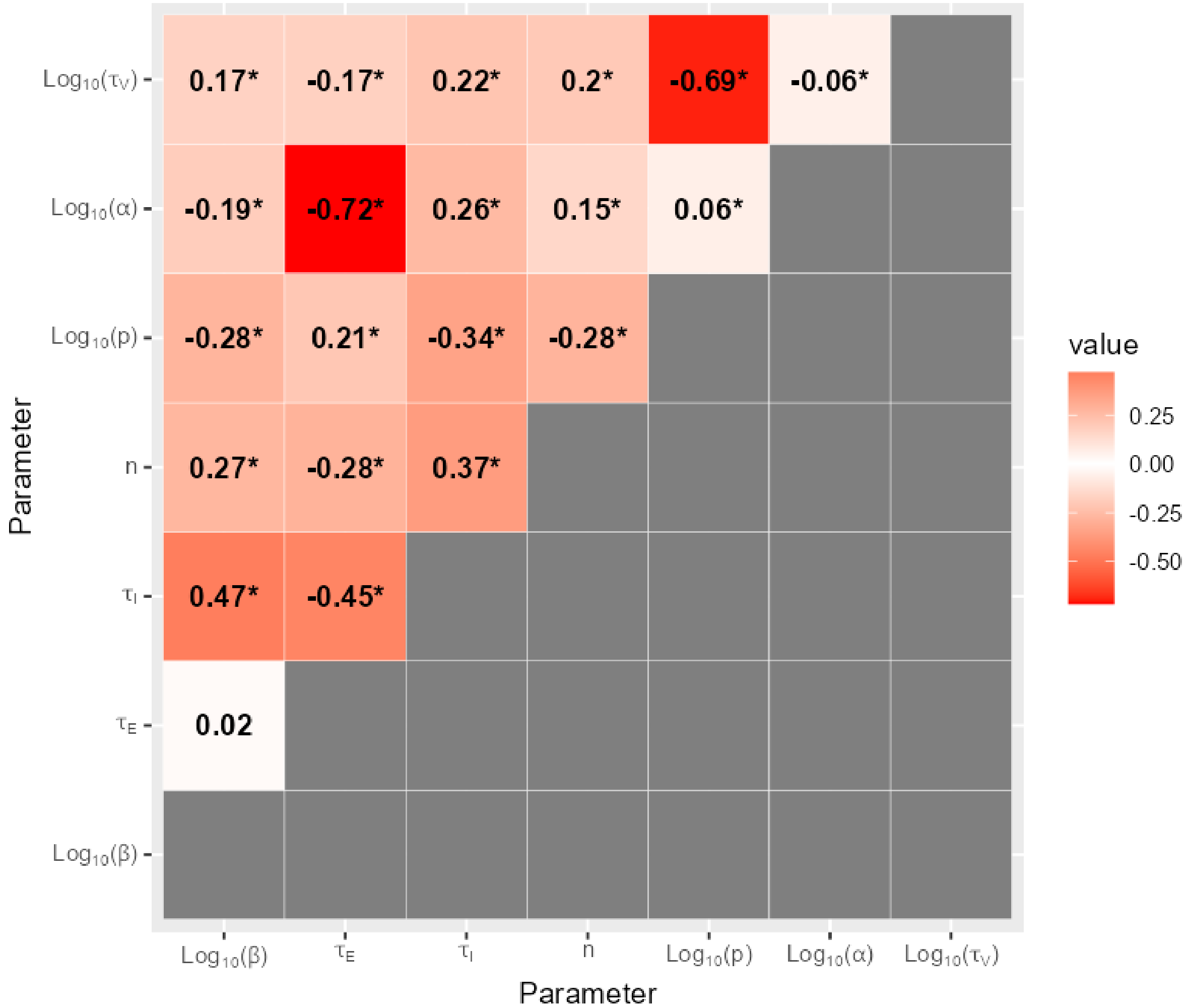

6.13 H5N8-2016 on CEF cells

Figure S41. The shifts in the distributions of model parameters during the ABC-SMC analysis of data from strain H5N8-2016 on CEF cells, when moving from the first particle population (containing accepted particles sampled from the prior distribution) to the posterior distribution via intermediate particle populations.

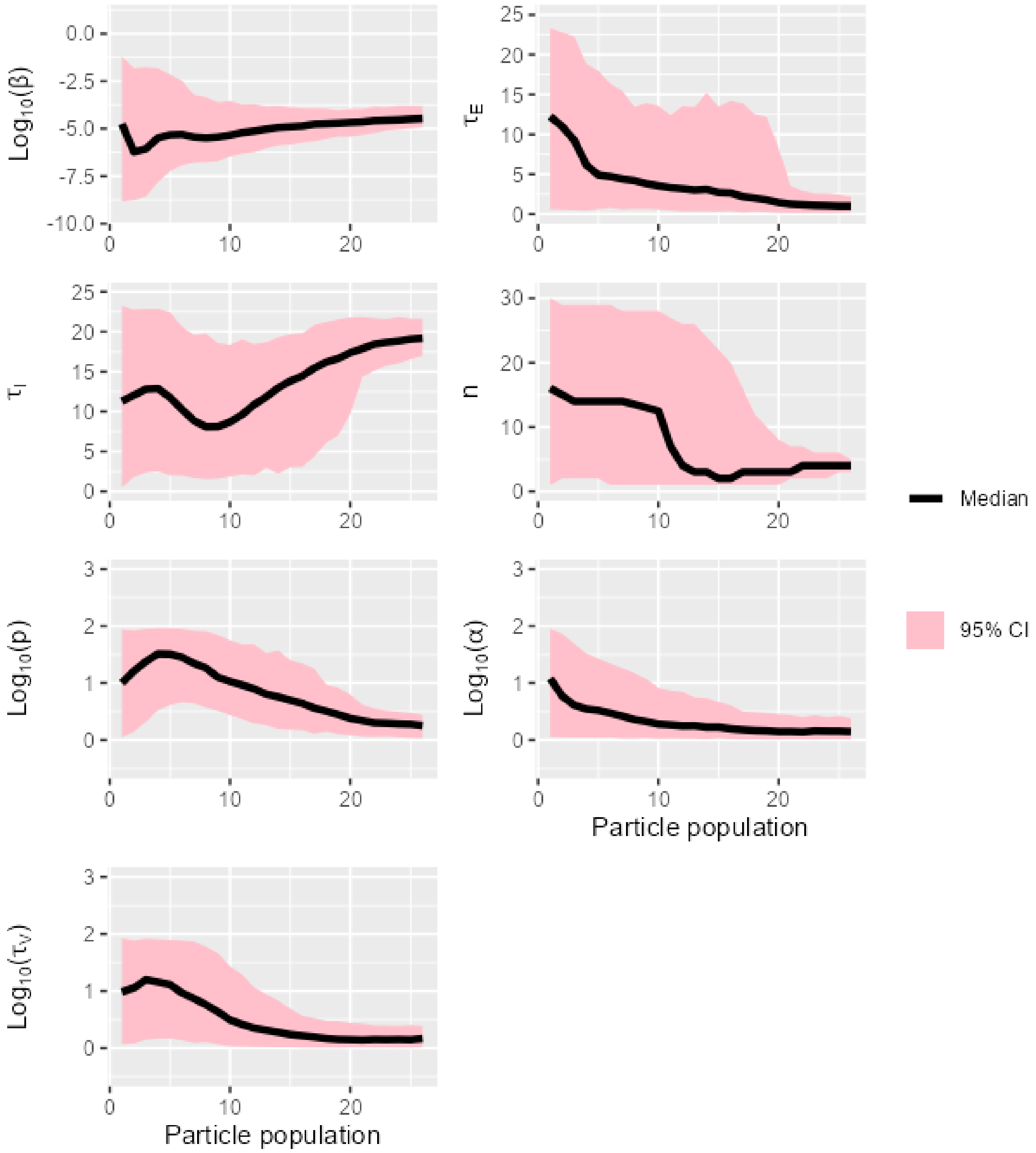

Figure S42. The overlap between prior and posterior distributions of model parameters for strain H5N8-2016 on CEF cells.

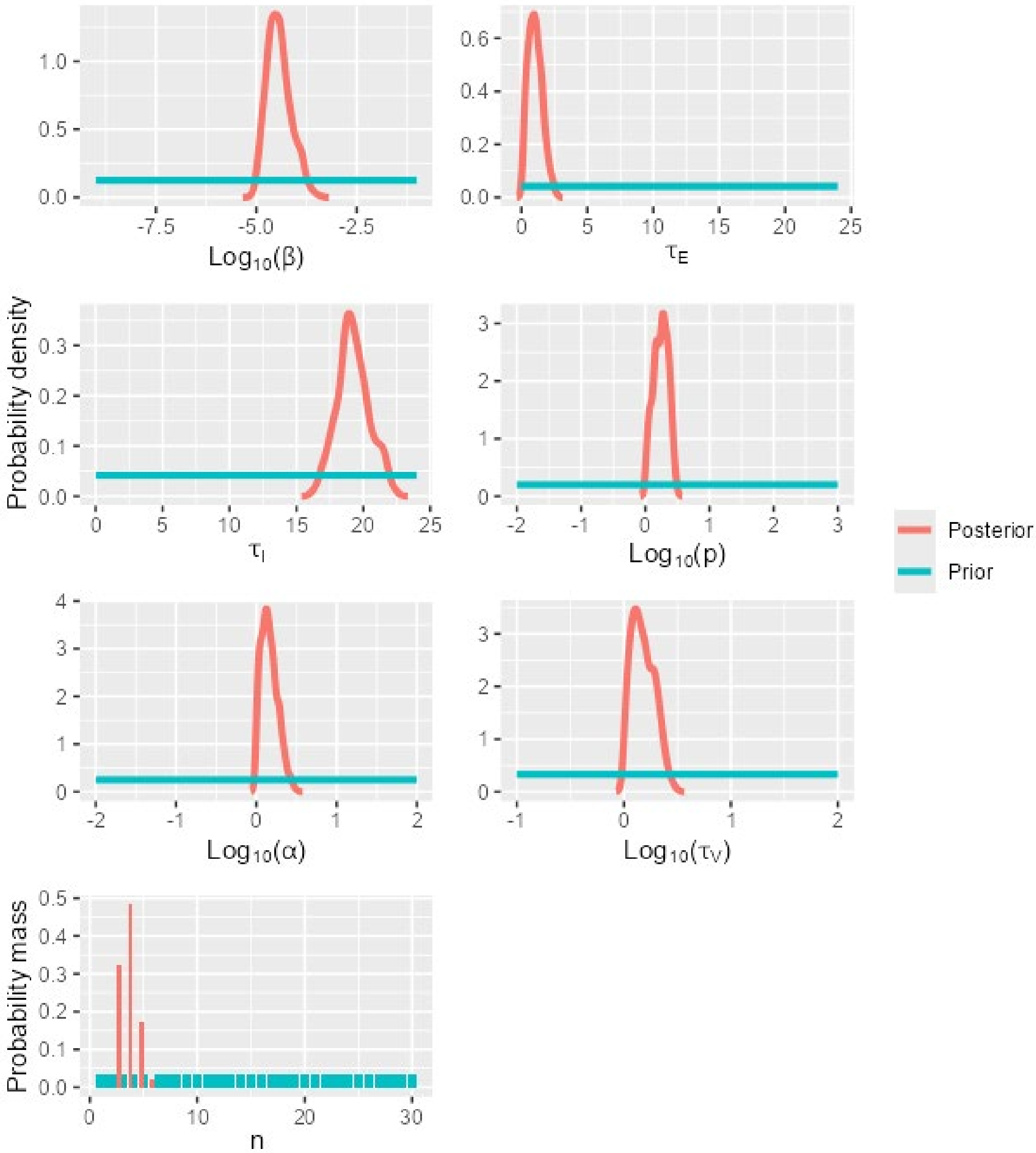

Figure S43. A matrix showing Kendall's rank correlation coefficient and the significance of the correlation (0.05 level, indicated by *) for pairs of model parameters calibrated using data for strain H5N8-2016 on CEF cells.

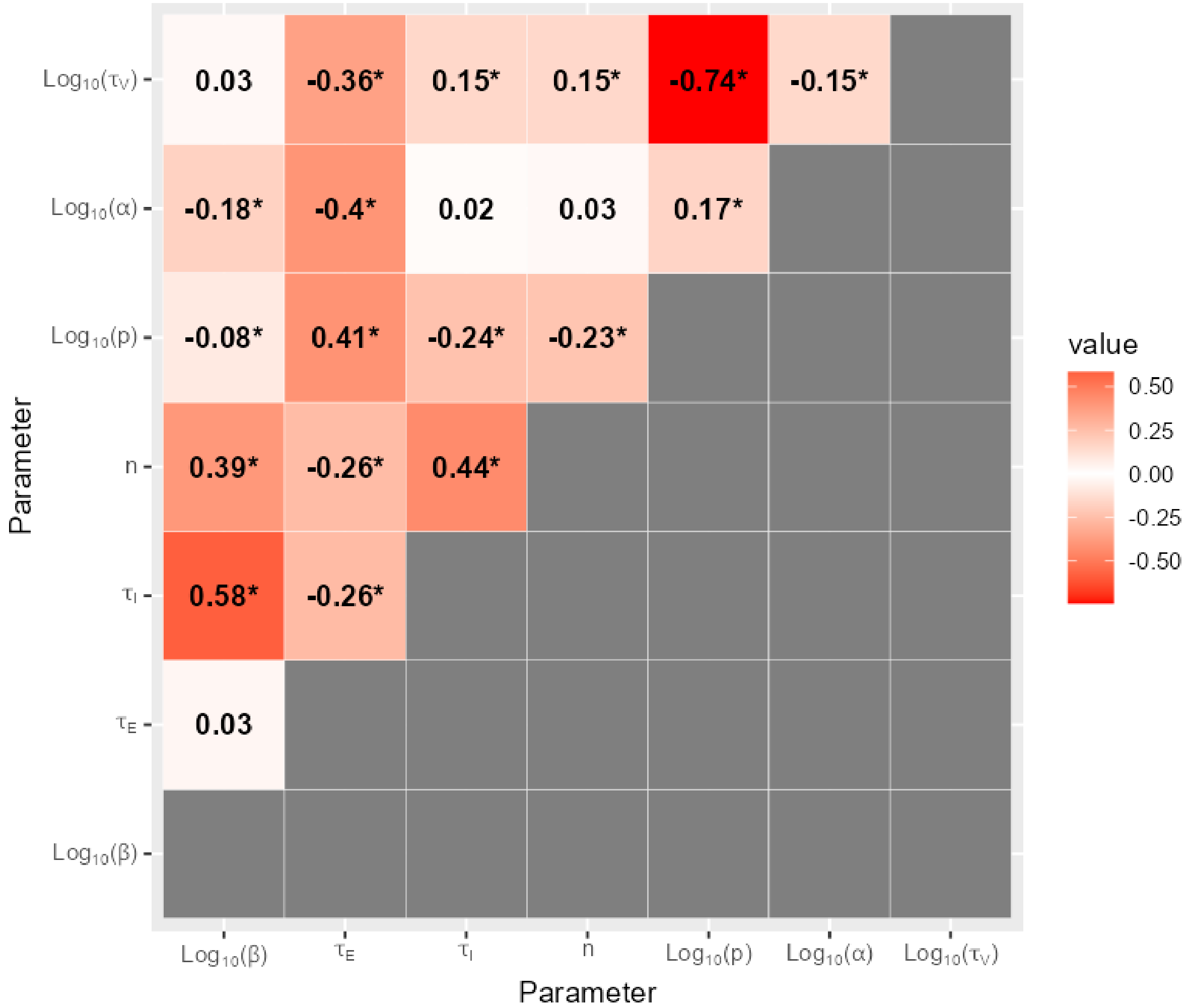

6.14 H5N8-2016 on DEF cells

Figure S44. The shifts in the distributions of model parameters during the ABC-SMC analysis of data from strain H5N8-2016 on DEF cells, when moving from the first particle population (containing accepted particles sampled from the prior distribution) to the posterior distribution via intermediate particle populations.

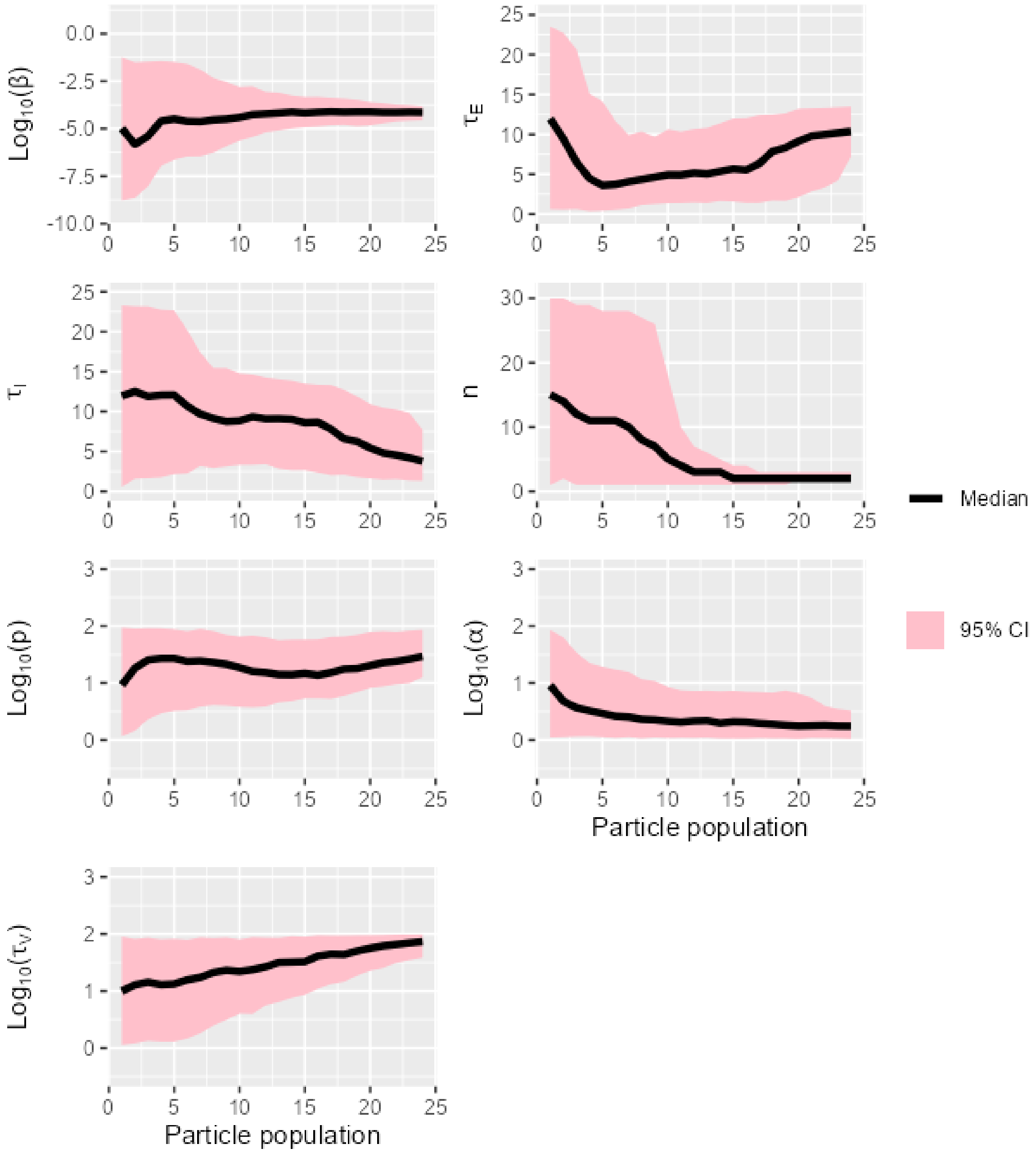

Figure S45. The overlap between prior and posterior distributions of model parameters for strain H5N8-2016 on DEF cells.

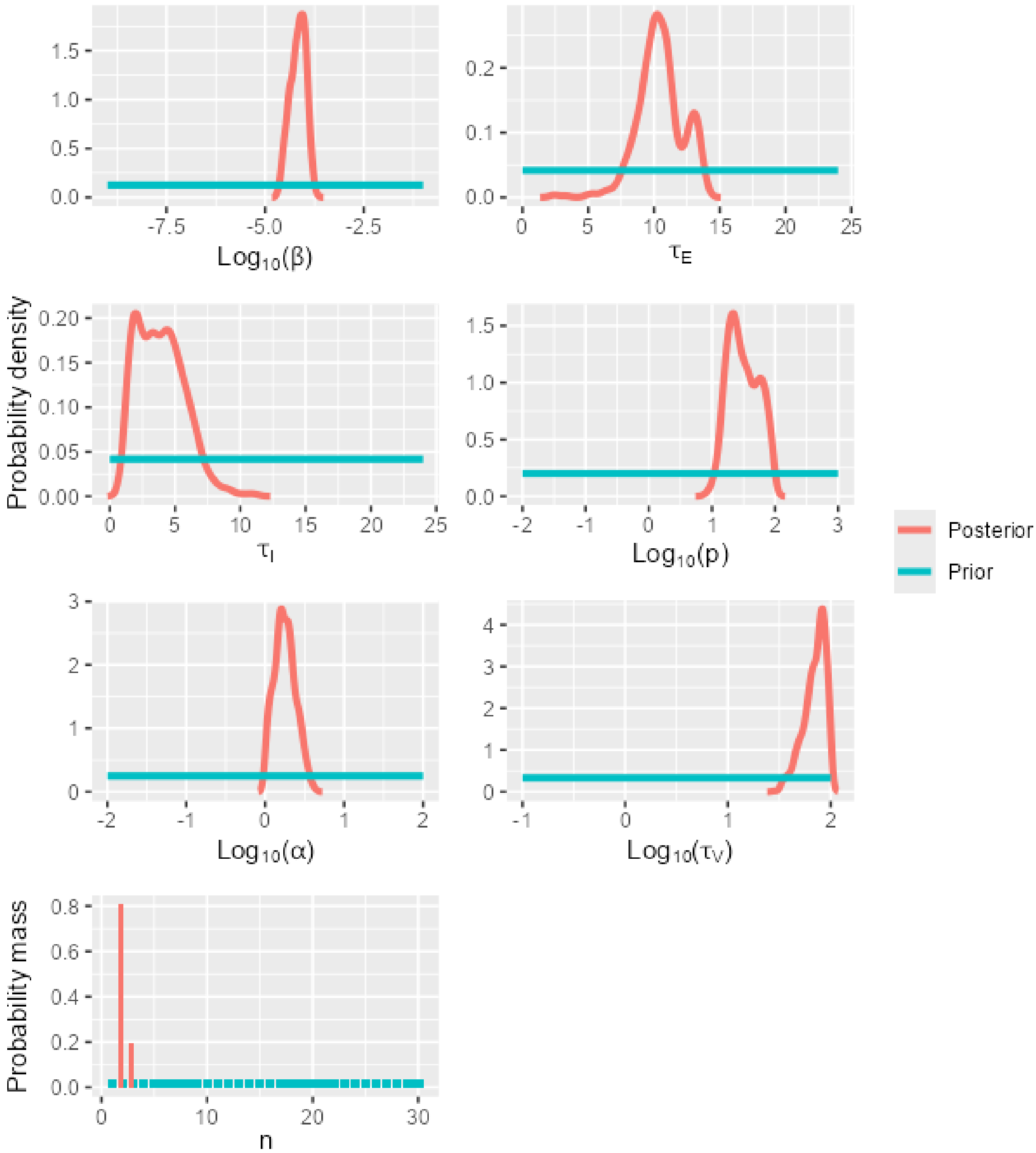

Figure S46. A matrix showing Kendall's rank correlation coefficient and the significance of the correlation (0.05 level, indicated by *) for pairs of model parameters calibrated using data for strain H5N8-2016 on DEF cells.

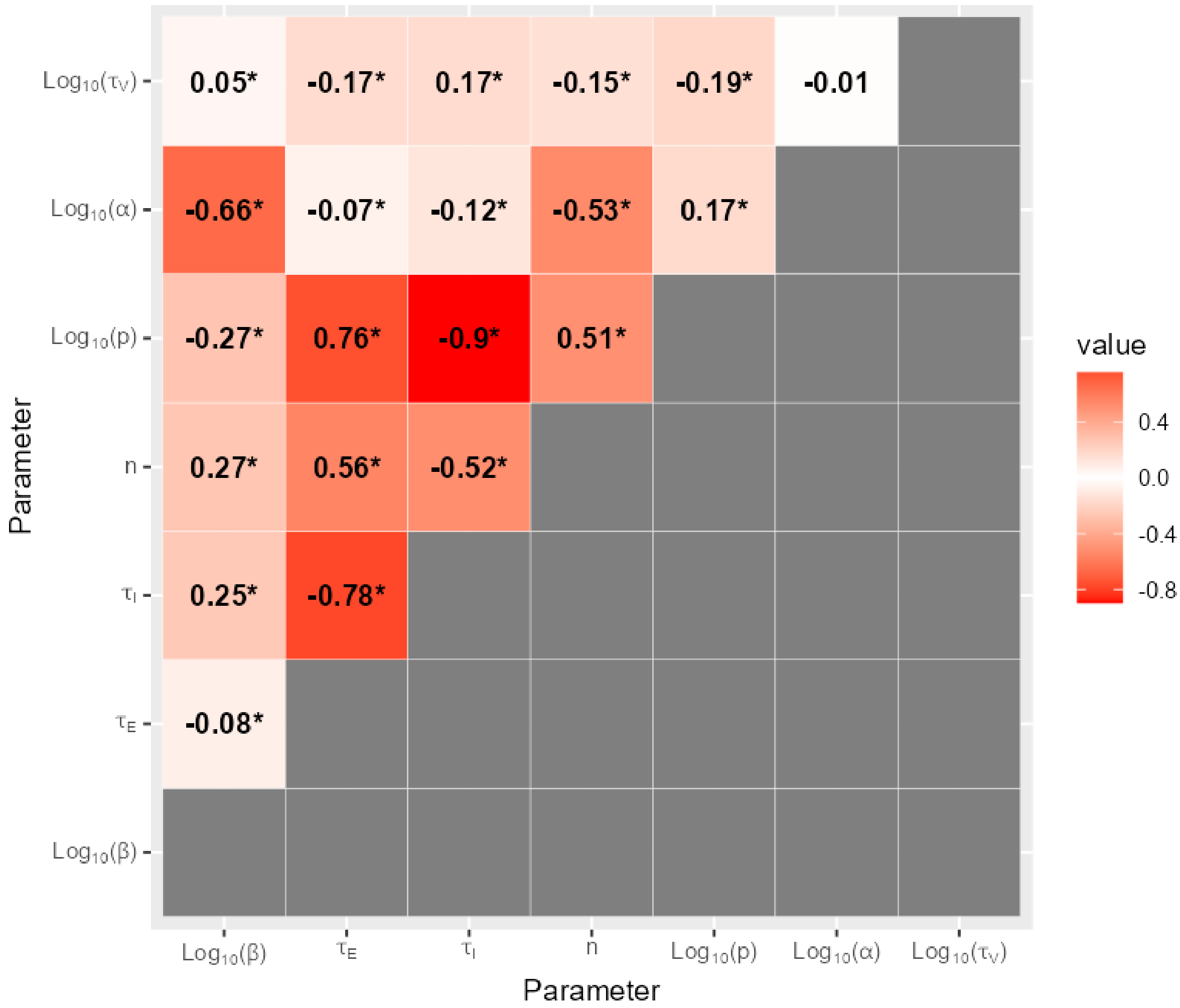

6.15 H5N8-2020 on CEF cells

Figure S47. The shifts in the distributions of model parameters during the ABC-SMC analysis of data from strain H5N8-2020 on CEF cells, when moving from the first particle population (containing accepted particles sampled from the prior distribution) to the posterior distribution via intermediate particle populations.

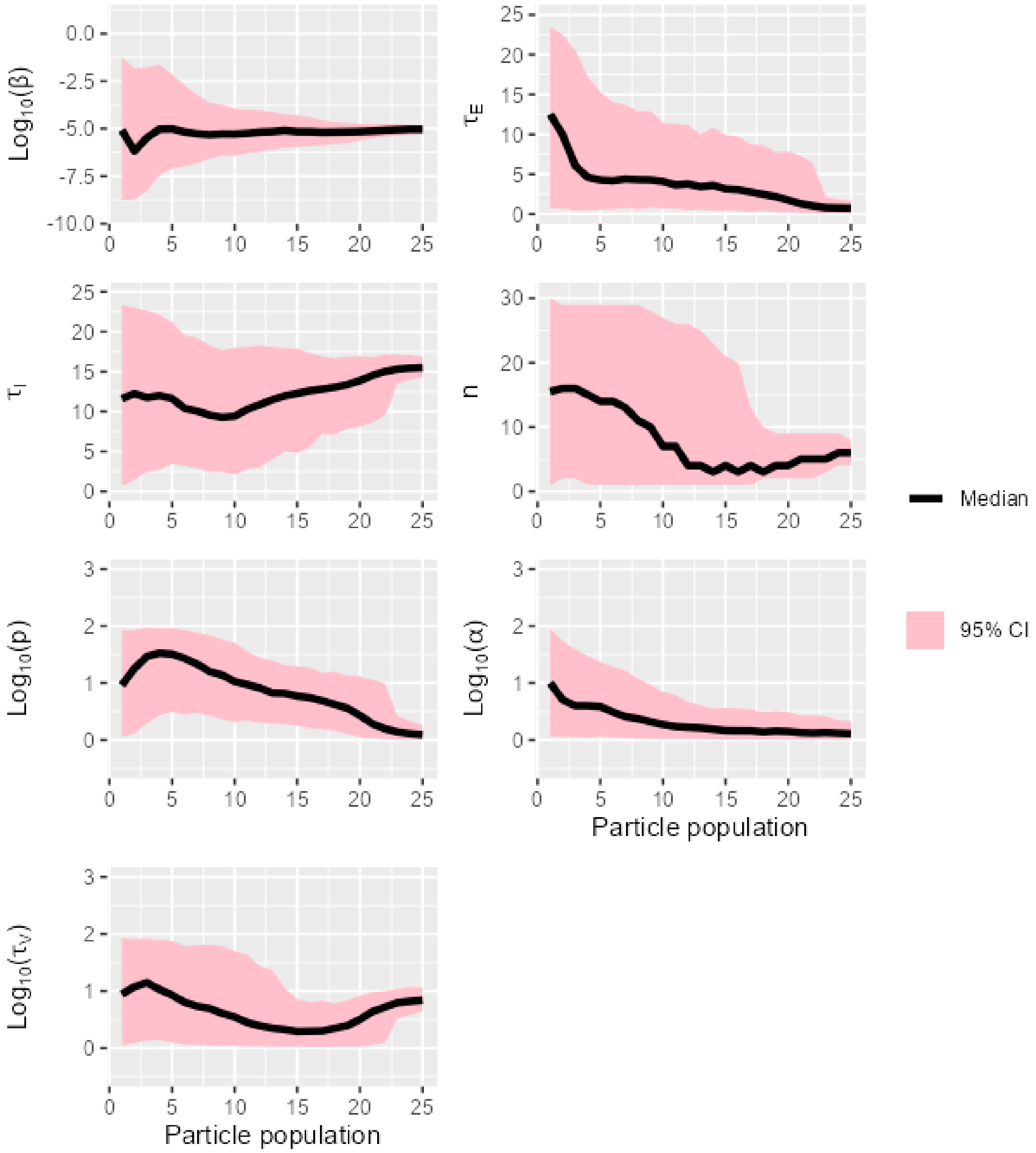

Figure S48. The overlap between prior and posterior distributions of model parameters for strain H5N8-2020 on CEF cells.

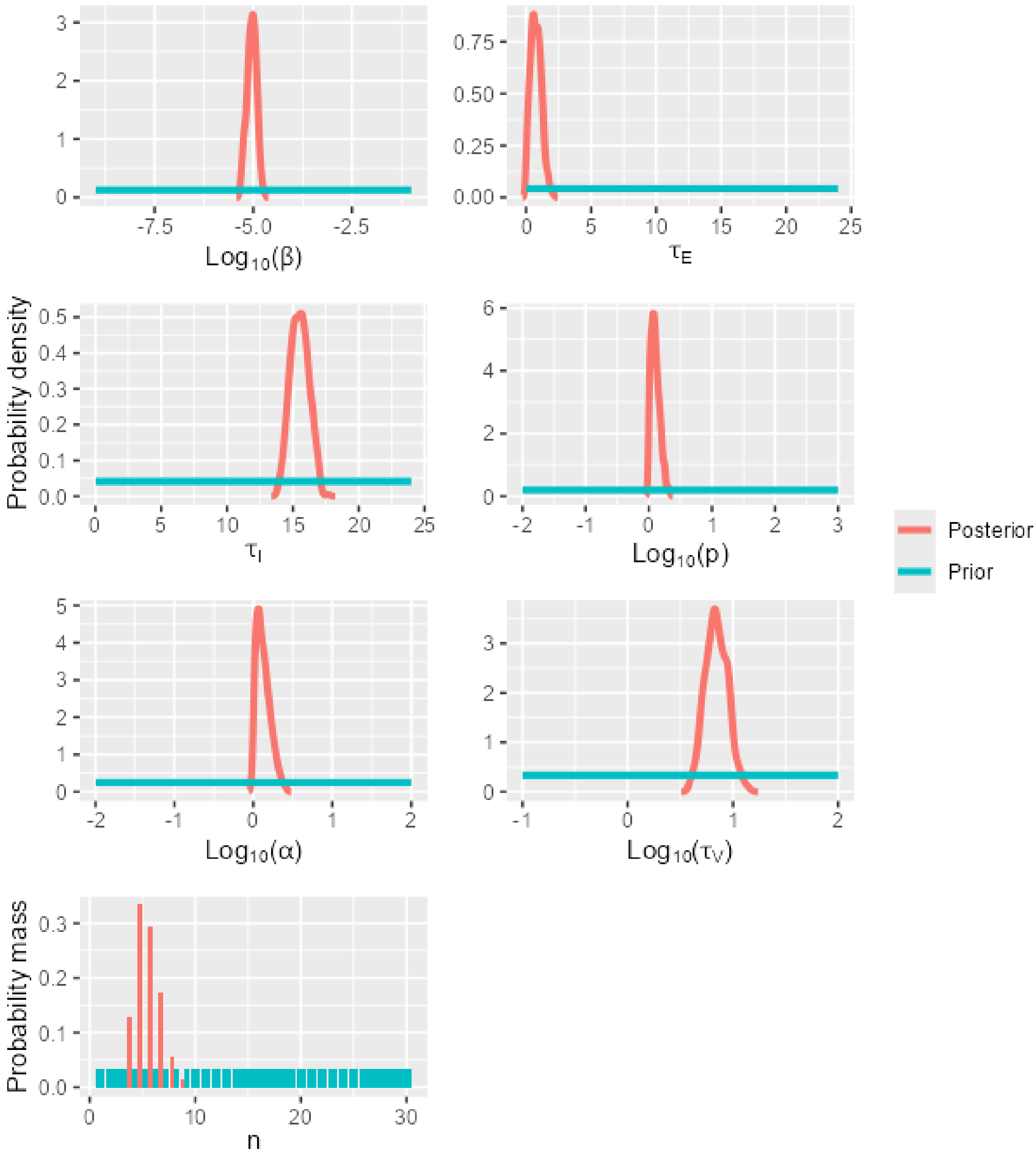

Figure S49. A matrix showing Kendall's rank correlation coefficient and the significance of the correlation (0.05 level, indicated by *) for pairs of model parameters calibrated using data for strain H5N8-2020 on CEF cells.

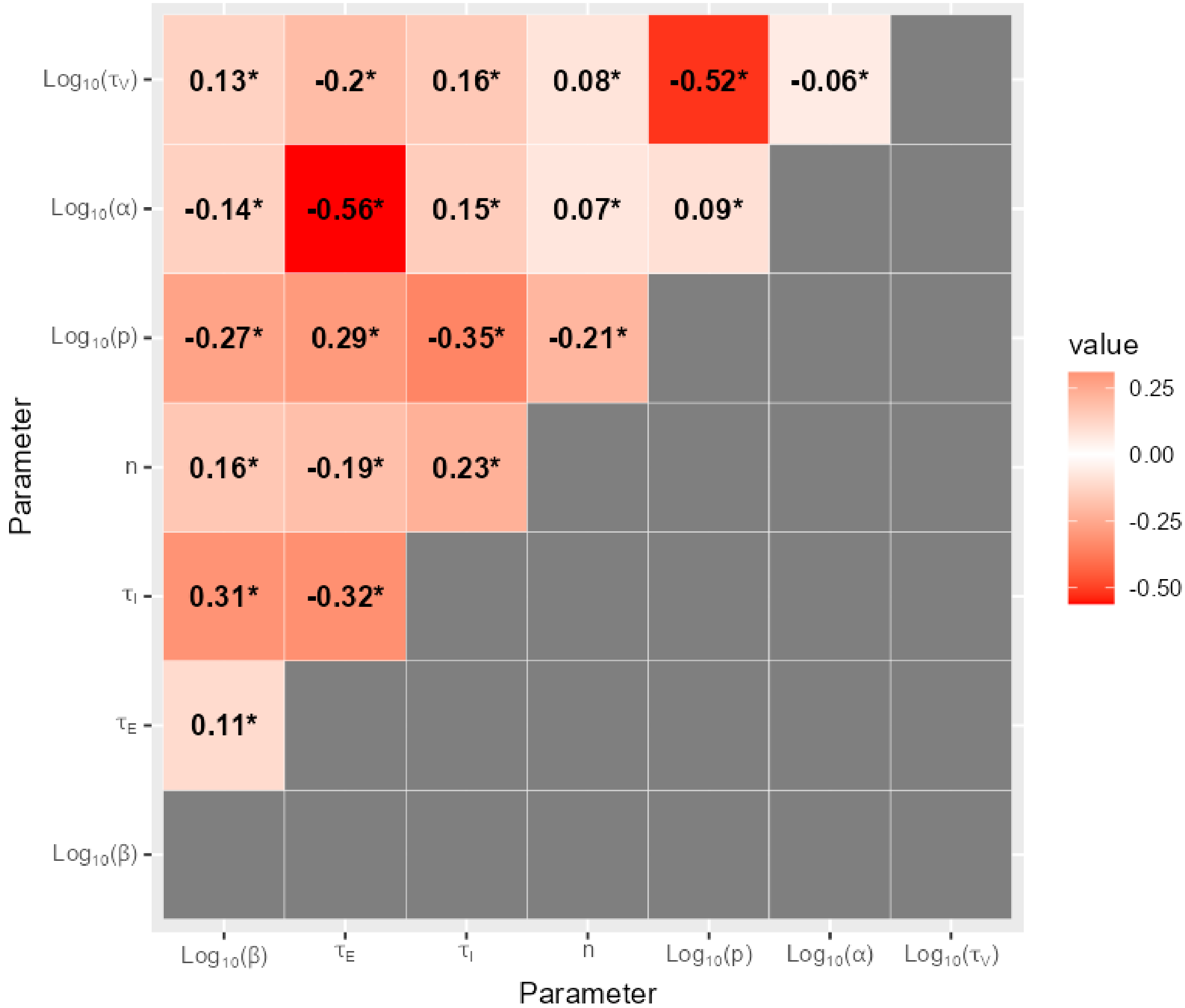

6.16 H5N8-2020 on DEF cells

Figure S50. The shifts in the distributions of model parameters during the ABC-SMC analysis of data from strain H5N8-2020 on DEF cells, when moving from the first particle population (containing accepted particles sampled from the prior distribution) to the posterior distribution via intermediate particle populations.

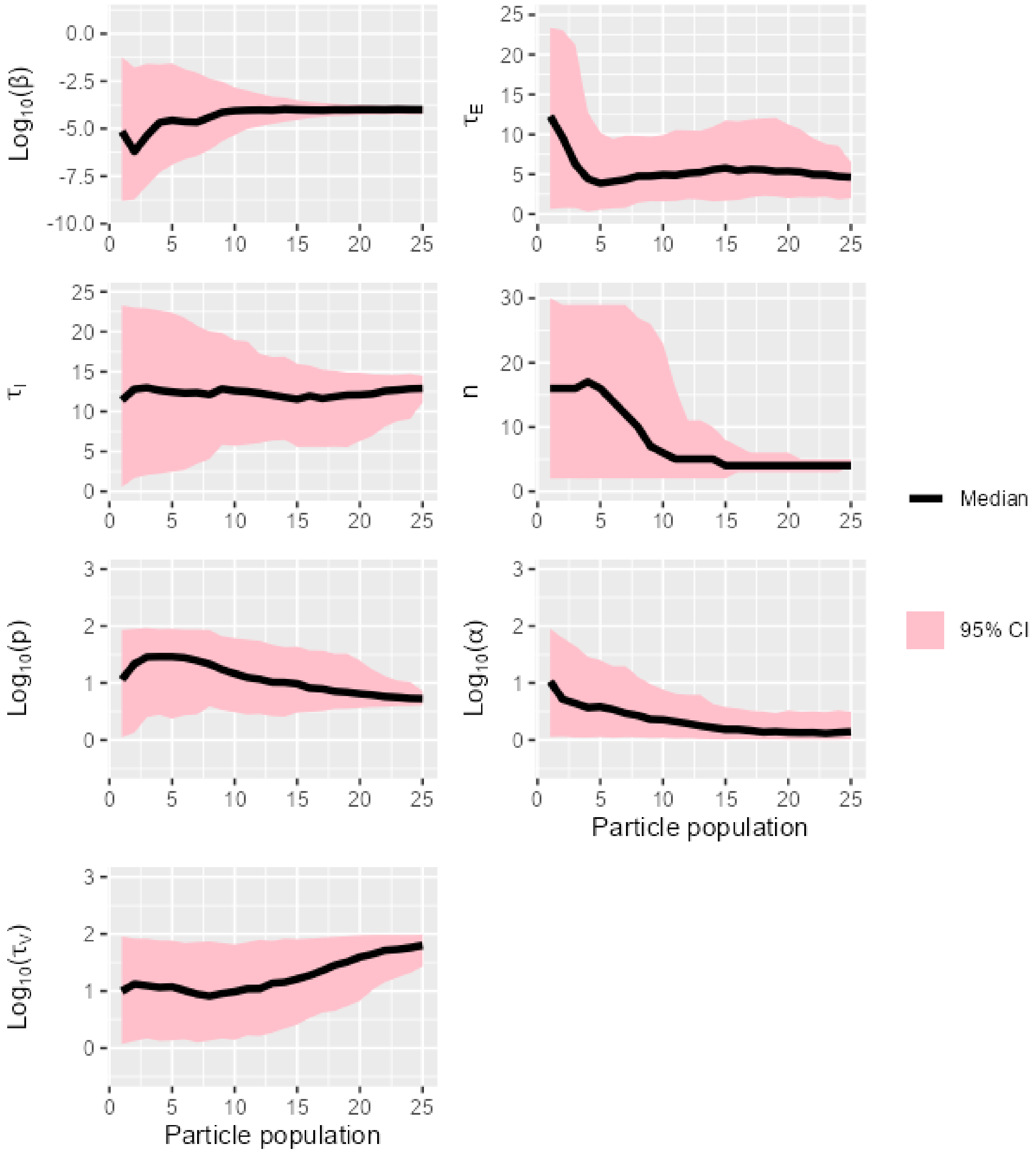

Figure S51. The overlap between prior and posterior distributions of model parameters for strain H5N8-2020 on DEF cells.

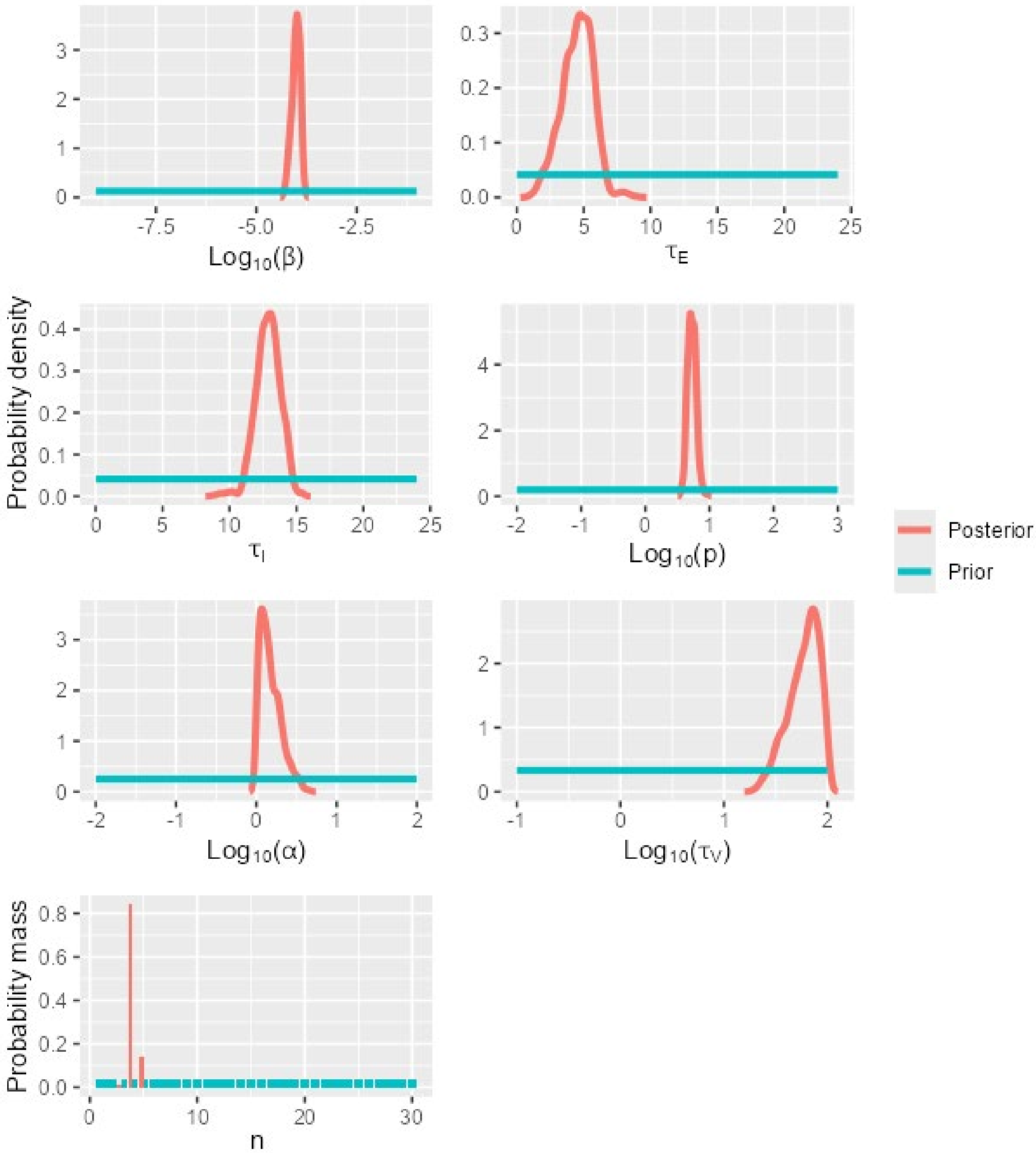

Figure S52. A matrix showing Kendall's rank correlation coefficient and the significance of the correlation (0.05 level, indicated by *) for pairs of model parameters calibrated using data for strain H5N8-2020 on DEF cells.

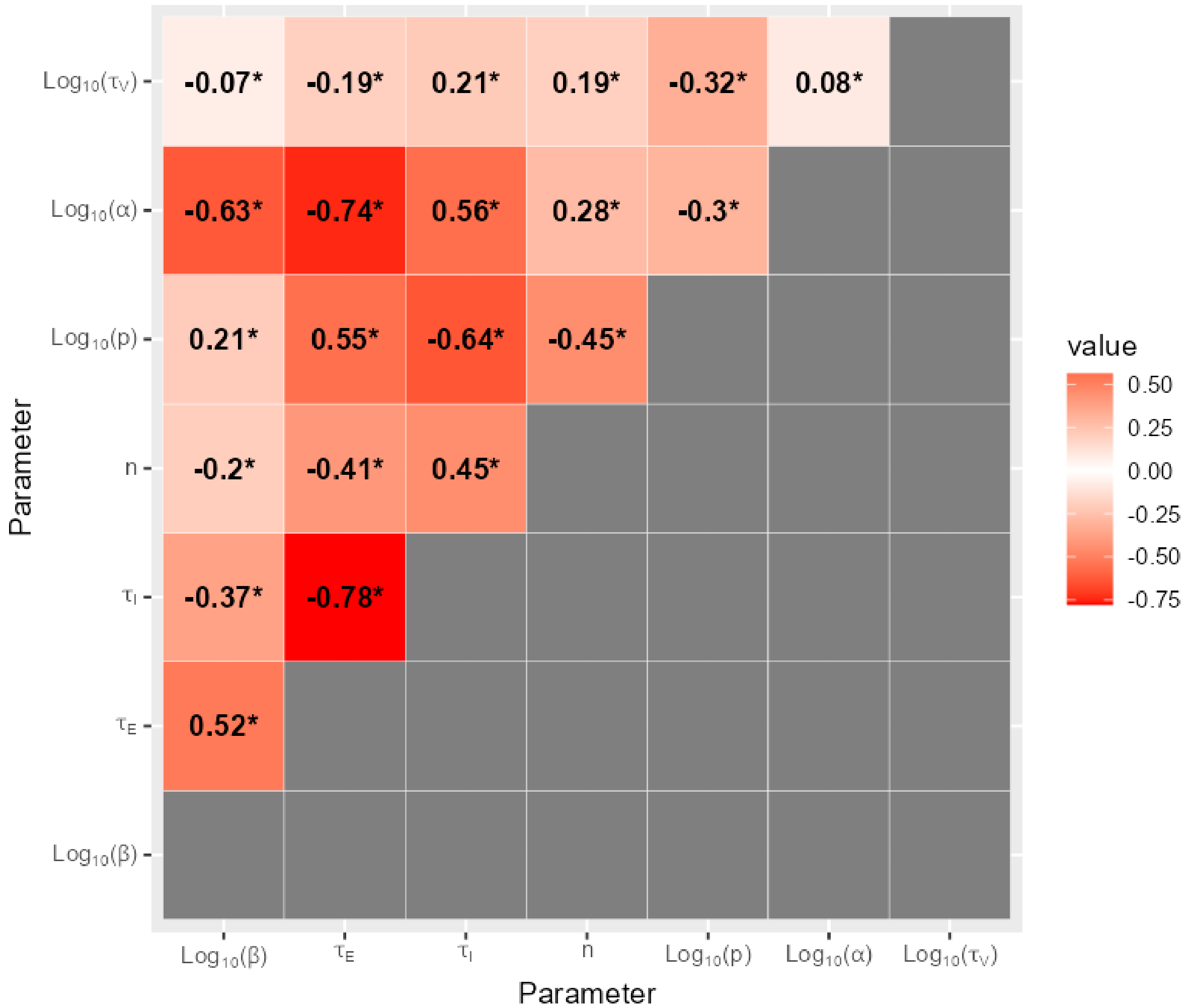

Supplement: Supplementary file 2 [file spectrum.00843-26-s0001.pdf]
